# Natural Products Derived Porous Carbons for CO_2_ Capture

**DOI:** 10.1002/advs.202304289

**Published:** 2023-10-31

**Authors:** Mobin Safarzadeh Khosrowshahi, Hossein Mashhadimoslem, Hadi Shayesteh, Gurwinder Singh, Elnaz Khakpour, Xinwei Guan, Mohammad Rahimi, Farid Maleki, Prashant Kumar, Ajayan Vinu

**Affiliations:** ^1^ Nanotechnology Department School of Advanced Technologies Iran University of Science and Technology (IUST) Narmak Tehran 16846 Iran; ^2^ Faculty of Chemical Engineering Iran University of Science and Technology (IUST) Narmak Tehran 16846 Iran; ^3^ Global Innovative Centre for Advanced Nanomaterials (GICAN) College of Engineering Science and Environment (CESE) The University of Newcastle University Drive Callaghan New South Wales 2308 Australia; ^4^ Department of Biosystems Engineering Faculty of Agriculture Ferdowsi University of Mashhad Mashhad 9177948974 Iran; ^5^ Department of Polymer Engineering and Color Technology Amirkabir University of Technology No. 424, Hafez St Tehran 15875‐4413 Iran

**Keywords:** adsorption, biomass, CO_2_ capture, machine learning, porous carbon, simulation, synthesis

## Abstract

As it is now established that global warming and climate change are a reality, international investments are pouring in and rightfully so for climate change mitigation. Carbon capture and separation (CCS) is therefore gaining paramount importance as it is considered one of the powerful solutions for global warming. Sorption on porous materials is a promising alternative to traditional carbon dioxide (CO_2_) capture technologies. Owing to their sustainable availability, economic viability, and important recyclability, natural products‐derived porous carbons have emerged as favorable and competitive materials for CO_2_ sorption. Furthermore, the fabrication of high‐quality value‐added functional porous carbon‐based materials using renewable precursors and waste materials is an environmentally friendly approach. This review provides crucial insights and analyses to enhance the understanding of the application of porous carbons in CO_2_ capture. Various methods for the synthesis of porous carbon, their structural characterization, and parameters that influence their sorption properties are discussed. The review also delves into the utilization of molecular dynamics (MD), Monte Carlo (MC), density functional theory (DFT), and machine learning techniques for simulating adsorption and validating experimental results. Lastly, the review provides future outlook and research directions for progressing the use of natural products‐derived porous carbons for CO_2_ capture.

## Graphical abstract

This review article highlights crucial insights and analyses to enhance the understanding of the application of porous carbons in CO_2_ capture. The review encompasses various aspects of porous carbons derived from natural precursors for CO_2_ capture such as precursors, synthesis, characterization, parameters affecting CO_2_ adsorption, and molecular simulation.

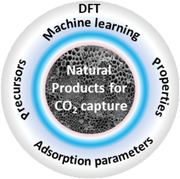

## 1 Introduction

**Figure 1:**
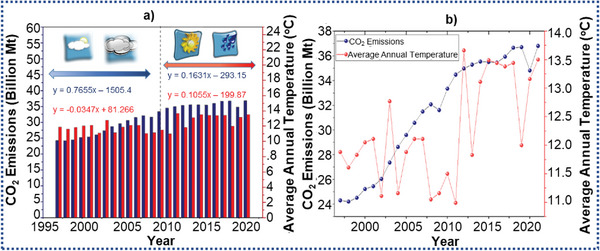
a,b) Graphs related to the increase in temperature and the amount of CO_2_ emissions in recent years (1997‐2022); CO_2_ emissions (blue color), global warming (red color).

Excessive emissions of CO_2_ result in an imbalanced rise in global temperature and hence global warming. The Inter‐governmental Panel on Climate Change (IPCC) and United Nations Framework Convention on Climate Change (UNFCCC) have released special reports on the continued rise of global temperature by around 1.5–2 °C.^[^ ^1^ ^]^ As displayed in **Figure** **1**a,b, the greenhouse effect causes a rise in the average surface temperature of the globe as a result of excessive greenhouse gas (GHG) emissions. It can also be seen in Figure 3 that there is a clear relation between the increment in temperature and the amount of CO_2_ emissions in recent years. The rise in the temperature due to CO_2_ emission causes natural calamities such as intense heatwaves, flash flooding, and elongated colder that occur across the world.^[^ ^2^ ^]^ In addition to CO_2_, nitrous oxide (N_2_O), methane (CH_4_), and chlorofluorocarbons (CFCs) are also GHGs that contribute to climate change. However, among these gases, atmospheric CO_2_ concentrations play a significant role in influencing climate change.^[^ ^3^ ^]^ CO_2_ emissions from the burning of fossil fuels, including petroleum, coal, and natural gas, account for 78% of total GHG emissions. CO_2_ emissions have risen alarmingly since the industrial revolution, according to the International Energy Agency (IEA). CO_2_ levels in the atmosphere are already larger than 400 ppm, which is 40% higher than in the mid‐1800s, with an average annual increase of 2 ppm in the previous 10 years, and are expected to exceed 570 ppm by 2100, putting our lives in jeopardy.^[^ ^4^ ^]^

Furthermore, CO_2_ emissions in the world grew by over 80% between 1970 and 2004. In 2022, 36.8 billion tons of CO_2_ were emitted into the atmosphere, resulting in an atmospheric CO_2_ concentration of >420 ppm in 2022, compared to 280 ppm in preindustrial times. According to recent National Aeronautics and Space Administration (NASA) research, such CO_2_ concentrations have already surpassed the point of irreversibility, meaning that it is no longer feasible to return to the pre‐industrial CO_2_ level.^[^ ^5^ ^]^

The huge rise in the CO_2_ level in the atmosphere has also resulted in significant consequences such as an increase in the frequency of extreme weather events, the melting of polar glaciers, and the extinction of species.^[^ ^6^ ^]^ Furthermore, the presence of N_2_ and CO_2_ contaminants in natural gas might lower its heating value and induce corrosion in equipment and pipelines.^[^ ^7^ ^]^ Therefore, it is critical to better understand the spatiotemporal patterns (input‐output from data driven‐statistics) and drivers of CO_2_ emissions to meet the emission reduction objective. For estimating future CO_2_ emissions and devising energy‐saving and emission‐reduction programs, data on the heating degree days and cooling degree days as well as the volume of CO_2_ emissions and historical CO_2_ emission levels is crucial. It should also be noted that population, economic growth, and industrial structures are other important factors that significantly contribute to CO_2_ emissions. In contrast, both energy intensity and energy consumption structure were found to have a negative impact on CO_2_ emission levels.^[^ ^8^ ^]^ As a result, lowering CO_2_ emissions is a top priority in the battle against global warming, and this will need the development of effective CO_2_ capture and sequestration technologies.

Industrialization, especially power plants, oil refineries, petrochemical, and cement industries, has generated considerable volumes of GHGs into the environment, potentially contributing to climate change and ocean acidification. CO_2_, as an acidic gas, accounts for 70% of the total GHGs emissions.^[^ ^9^ ^]^ Fossil fuel‐fired power stations are responsible for around 44% of CO_2_ emissions, with flue gas generally consisting of 85% nitrogen (N_2_) and 15% CO_2_. In order to conserve the environment and prevent imminent climate change, sustainable and eco‐friendly solutions must be developed to cut CO_2_ emissions.^[^ ^10^ ^]^

One of the most efficient ways to reduce emissions is to replace fossil fuels with renewable energy sources and improve energy efficiency.^[^ ^11^ ^]^ Renewables, on the other hand, suffer from unpredictability and volatility. Despite several attempts across the globe including the new push on renewable energy based on solar cells, clean energy storage and conversion systems, and hydrogen technologies, fossil fuels remain the most cost‐effective and offer a stable energy source but with a huge emission of CO_2_.^[^ ^12^ ^]^ Therefore, it is critical to develop technologies for the effective capture of CO_2_ molecules which is the major culprit for global warming. While this manuscript primarily focuses on the utilization of porous carbon as a material for CO_2_ capture, it is essential to keep in mind that the integration of both CO_2_ capture and conversion plays a pivotal role in successfully mitigating CO_2_ emissions and associated costs. The captured CO_2_ must possess a high level of purity to facilitate its effective utilization in downstream CO_2_ conversion technologies for the creation of marketable products. CO_2_, being an inert and highly stable molecule presents challenges for its conversion into other products.^[^ ^13^ ^]^ Among the array of CO_2_ conversion technologies, the most prominent ones are thermocatalytic,^[^ ^14^ ^]^ electrochemical,^[^ ^15^ ^]^ and photocatalytic^[^ ^16^ ^]^ processes. However, the former two methods tend to be costlier due to their energy‐intensive nature. On the other hand, photocatalytic conversion stands out as a highly promising avenue for realizing a nearly zero‐emission CO_2_ conversion technology. Through photocatalytic water splitting, the produced hydrogen can be employed in the reaction with CO_2_ to yield valuable chemicals such as methane (CH_4_) and methanol (CH_3_OH). However, the pursuit of discovering a cost‐effective catalyst that possesses high efficiency, selectivity, and yield remains a constant focus at the forefront of research.

**Figure 2:**
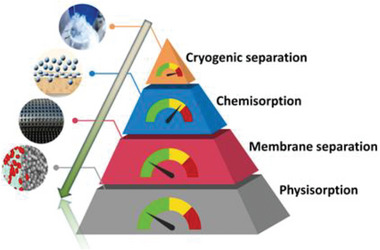
The different types of post‐combustion CO_2_ capture methods.

Post‐combustion, pre‐combustion, and oxy‐fuel combustion are the three primary technologies for CO_2_ capture that are employed in different types of operations. Post‐combustion capture (PCC) possesses the greatest potential for CO_2_ emission mitigation since it can be easily retrofitted in both existing and newly constructed power stations owing to compatibility with desulfurization and denitrification devices.^[^ ^17^ ^]^ Various sorption and separation technologies including chemisorption (amine scrubbing/ionic liquid absorption), physisorption, cryogenic separation, and membrane separation have been developed for the PCC, as shown in **Figure** **2**. Among these techniques, chemisorption using aqueous amines is one of the methods that has widely been used in industry to capture CO_2._^[^ ^18^ ^]^ However, a lot of energy is required to regenerate the adsorbents from the saturated chemical absorbents which are corrosive by nature.^[^ ^19^ ^]^ On the other hand, membrane‐based CO_2_ capture provides benefits such as low energy consumption and economic cost during the gas capture process, but it is insufficient for large feed flow rates, is often obstructed with dust, and has a lower CO_2_/N_2_ selectivity. Cryogenic separation is another unique separation process as it has the capability to generate liquid CO_2_ which can be good for easy transportation but the operation cost for this process is quite high due to the high energy regeneration and the energy needed for the low temperature. There are a lot of advantages to the physisorption of CO_2_ using solid adsorbents as it is a low‐cost process because it requires less energy and most importantly, the desorption process is quite easy owing to the weak bond between the adsorbent and the CO_2_ molecules. In light of these advantages of physisorption process and the aforementioned drawbacks for the chemisorption, and cryogenic and membrane separation, it is imperative to reconsider the CO_2_ capture approach through adsorption process using solid adsorbents.

**Figure 3:**
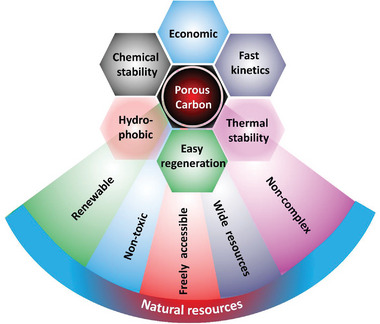
Illustration of the advantages of choosing natural precursors for synthesizing porous carbons that find use as CO_2_ adsorbents.

Because of the ease of maintenance, low energy demand, and great renewability, physisorption has widely been recognized as an effective technique for reversibly adsorbing CO_2_.^[^ ^20^ ^]^ Porous materials such as zeolite, halloysite,^[^ ^21^ ^]^ kaolinite, mesoporous carbon nitrides,^[^ ^22^ ^]^ metal‐organic frameworks (MOFs), metal‐organic polyhedra (MOPs), graphene oxide, porous organic polymers (POPs), and natural products‐derived porous carbons have recently been synthesized as sorbents for CO_2_ adsorption.^[^ ^18^, ^23^ ^]^ The simplicity of their synthesis, customizable pore structure, low acid/base reactivity, hydrophobicity, low energy intensity, and easy renewability have collectively garnered significant interest in CO_2_ physisorption utilizing these porous materials. Materials such as zeolites, clays, MOFs, MOPs, and POPs show good promise for CO_2_ adsorption at low pressure, however, their stability under humid environments is a critical issue. The mesoporous carbon nitrides are good candidates for CO_2_ adsorption at high pressures, however, their microporosity can be enhanced through suitable manipulation to lift their CO_2_ adsorption ability at lower pressures as well. Amongst all porous materials listed above, non‐toxic and economical natural products (biomass/solid fossil fuel)‐derived porous carbons with unique structural features and chemical stability are regarded to be the most promising CO_2_ sorbents, which can be generated by pyrolysis from a wide range of diverse C‐rich precursors derived from the natural products.^[^ ^23^, ^24^ ^]^ Considering the mentioned advantages, the integration of porous structures with natural precursors leads to the construction of porous materials with unique properties.^[^ ^25^ ^]^ **Figure** **3** shows a schematic of the benefits of natural precursors as a carbon resource and the porous carbons derived from them as adsorbents for CO_2_ capture. Since the majority of biomass/solid fossil fuels are from agricultural wastes, forestry harvests, and various agro‐bio industries, efficient utilization of these resources may become necessary as they have the potential to mitigate environmental problems.^[^ ^26^ ^]^

Several porous carbons derived from natural products such as coal, tar, coffee, algae, celery, celtuce leaves, rice husk, anthracites, eucalyptus wood, etc. can be prepared by carbonization and activation for CO_2_ capture.^[^ ^27^ ^]^ Conventional methods such as chemical and physical activation, microwave, hydrothermal, and sol‐gel are extensively used to activate/carbonize natural waste into porous carbons in a one or two steps process. Besides, new methods such as self‐activation, template carbonization, and plasma have also emerged, which are more advanced and more advantageous compared to traditional methods.^[^ ^28^ ^]^ The nature of the precursor (properties of carbon‐rich sources and heterogeneity), carbonization conditions, activation agents, and the pre‐activation process are important parameters that influence the physicochemical qualities of the final porous carbons.^[^ ^29^ ^]^ However, it is important to note that adsorbents that are acceptable for PCC must fulfill certain criteria such as high CO_2_ adsorption capacity at low CO_2_ partial pressures (0.15 atm–25 °C), high separation of CO_2_ over N_2_ (S_CO2/N2_), moderate adsorption heat (Q_st_), and rapid adsorption kinetics.^[^ ^30^ ^]^ Porous carbons possessing both meso‐ and macroporous structures are highly regarded due to their ability to facilitate rapid transport of gas molecules. On the other hand, porous materials featuring narrow micropores (with diameters less than 2 nm), especially ultramicropores (with diameters under 0.7 nm), play a crucial role in augmenting the interaction between CO_2_ molecules and the surface of the adsorbent. Consequently, these ultramicropores are identified as the primary sites for adsorption. This underscores the significance of the interconnected meso/macropores and the microporous framework in ensuring a substantial adsorption capacity.^[^ ^3^ ^]^ Moreover, the adsorption properties of the porous carbon are related to surface chemistry such as heteroatoms (e.g., oxygen, nitrogen, boron, sulfur, and phosphorus) and chemical properties (surface functional groups, acidity, and alkalinity) which can influence the charge distribution of the carbon surface.^[^ ^31^ ^]^ As CO_2_ is an acidic gas with a high quadrupole moment, adding basic nitrogen functionalities to the carbon skeleton can improve the affinity and interactions between CO_2_ gas molecules and sorbent.^[^ ^32^ ^]^

While porous carbons primarily facilitate CO_2_ adsorption through physisorption at moderate temperatures, the challenge of enhancing their capacity to adsorb significant quantities of CO_2_ at elevated temperatures can effectively be tackled by introducing basic functional groups, such as amines, onto their surfaces. In such cases, the nature of adsorption changes to chemisorption because of strong interactions between CO_2_ molecules and surface‐grafted ‐NH_2_ groups. Nonetheless, the exact involvement and contributions of each variable in the textural characteristics of CO_2_ adsorption remain unknown. To determine the link between CO_2_ adsorption of porous carbons and relevant textural features, as well as the optimization of the synthesis process, the deep neural network is developed as a generative model.^[^ ^33^ ^]^ The developed neural network is also used as an explicit model to predict the CO_2_ sorption capacity of unknown porous carbons. Many machine learning techniques (Linear regression, support vector machines (SVMs), tree‐based, k‐nearest neighbors, and artificial neural networks (ANNs)) are capable of detecting complicated non‐linear correlations between many influencing parameters and correlated variables.^[^ ^1^, ^34^ ^]^ Furthermore, various simulation/modeling of the porous carbons and molecular simulation such as Monte Carlo (MC), molecular dynamic (MD), and density functional theory (DFT) simulation are developed to predict the adsorptive capability of optimal porous carbons with different surface chemistry at high pressures and temperatures.^[^ ^35^ ^]^ Eventually, adsorption isotherms are valuable in studying the mechanism of adsorption. The thermodynamic data derived from adsorption isotherms is critical for developing an adsorption process (such as pressure swing and temperature swing) for CO_2_ separation from fuel gases and determining adsorption efficiency.^[^ ^36^ ^]^

For the last few decades, there has been a lot of work on the synthesis of advanced porous carbon materials with different functionalities and their use for carbon capture applications. There are also a lot of reports on machine learning and molecular dynamics that have been widely used to understand the adsorption process and further to estimate the adsorption amount over the natural product‐derived porous adsorbents. Even with these new developments, there is a dearth of reviews covering all aspects of natural products‐derived porous carbon. In this review, we summarize the synthesis of various porous carbons from different types of natural precursors and outline the conventional and novel techniques of synthesizing/pyrolyzing them to obtain porous carbon for CO_2_ adsorption. Then, we identify the elements that influence adsorptive capabilities of the materials, such as morphology, porosity, and functional groups, and classified them into two categories: intrinsic and non‐intrinsic characteristics. Besides, we introduce simulation methods (Machine learning and molecular dynamics) that are used to estimate the amount of adsorption and to find the optimal adsorbent. Finally, we examine the various adsorption methods and show the different fitted isotherms. The assessment criteria for actual application are then discussed, including adsorption heat, diffusion kinetics, and thermodynamic performance.

## 2 Precursors

Adsorption using porous solid‐based adsorbents is one of the key solutions for tackling CO_2_ emissions. Among the porous adsorbents, porous carbons derived from natural biomass have been given much attraction owing to their excellent textural parameters, low cost, and the easy availability of a large amount of low‐cost raw materials. However, the choice of the initial precursor is a critical aspect of making these porous carbons with highly developed texture as every naturally derived precursor or synthetic raw material has different constituents (**Table** **1**).

**Table 1:** Elements present in natural precursors.

Natural precursor	Elements present	Reference
Coal	Carbon, hydrogen, oxygen, nitrogen, sulfur, and trace amounts of various other elements and inorganic impurities	[37]
Almond shell	Carbon, oxygen, nitrogen, and silicon	[38]
Onion skin	Carbon, oxygen, hydrogen, sulfur, potassium, calcium, phosphorous, sodium and magnesium	[39]
Rice straw	Carbon, nitrogen, silicon oxygen, and hydrogen	[40]
Wheat straw	Carbon, hydrogen, nitrogen, and sulfur	[41]
Chitosan	Carbon, hydrogen, nitrogen, and sulfur	[42]
Hemp fibres	Carbon and oxygen	[43]
Coconut shell	Carbon, oxygen, and hydrogen	[44]
Loofah sponge	Carbon, oxygen, and hydrogen	[45]
Eucalyptus leaves	Carbon, oxygen, and trace amounts of sodium, magnesium, aluminum, silicon, calcium, and potassium	[46]

**Figure 4:**
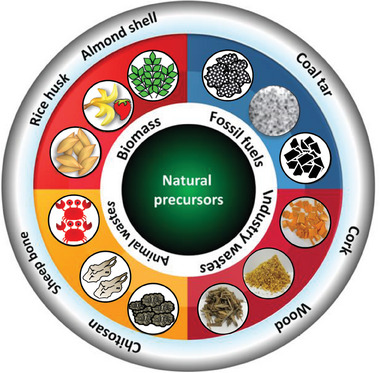
Various categories of natural carbon‐containing precursor types.

Furthermore, employing inexpensive, freely accessible, and renewable precursors could greatly lower the entire cost of CO_2_ capture from an economical perspective.^[^ ^47^ ^]^ Natural and synthetic precursors can both be used to synthesize porous carbon, however, natural precursors are more commonly used due to their wider resources, easy availability, and environment‐friendly.^[^ ^48^ ^]^ Synthetic raw materials exhibit a structure with a specific chemical composition and suffer from drawbacks such as limited resources, being an unsustainable byproduct, high cost, the complexity of the material composition process, and the requirement for special pre‐treatments.^[^ ^49^ ^]^ On the other hand, renewable natural precursors are generally divided into categories such as biomass, agricultural wastes, solid fossil fuels, municipal‐industrial wastes, and animal wastes, as depicted in **Figure** **4**.^[^ ^50^ ^]^

Optimal use of natural waste not only solves environmental concerns caused by surplus wastes released into nature, such as contamination of water, soil, and air, but it also lowers the cost of the synthesis of porous carbon. It also generates revenue through the sale of naturally derived porous carbons to various industries and reduces ancillary costs such as landfilling or incineration of wastes.^[^ ^51^ ^]^ The most important feature of these precursors is the abundance of carbon in their structure which can be etched during activation/pyrolysis to achieve a porous structure. Furthermore, raw biomass contains nitrogen and other intrinsic heteroatoms that will be anchored inside or surrounding the graphitic structure to provide a variety of functionalities to porous carbons.^[^ ^52^ ^]^ However, owing to the unknown structures of many precursors, the surface chemistry and pore sizes may be unpredictable. The role of these precursors not only controls the final structure and morphology of the porous carbons but also offers a lot of surface functionalities and heteroatoms such as N, O, and S onto the carbon framework.^[^ ^53^ ^]^ A wide range of natural precursors including nutshells (Almond shell, foxnut, and Terminalia Arjuna nuts), coals, fruit‐nutrient wastes (Rice husk, popcorn, olive stones, pomelo peel, celery, and celtuce leaves), wood‐flower residues (Arundo donax, willow catkins, elm leaves), and animal‐human wastes (Human hair, sheep bones, cigarette butts) which have different chemical structures and heteroatoms have been utilized for conversion into porous carbons with different morphological features, porosities and surface functionalities.^[^ ^54^ ^]^

### 2.1 Solid Fossil Fuels

Solid fossil fuels are rich in carbon and are available in large quantities and of low cost. Due to their affordability and high carbon content, coal stands out as the predominant C‐rich precursor among solid fossil fuels for the production of porous carbons with a high carbon yield. Coal, mainly constituted of polycyclic aromatic hydrocarbons, is a natural precursor for soft carbon, which has planar carbon layers and a short inter‐layer gap.^[^ ^55^ ^]^ The fundamental disadvantage of employing coal as a precursor is that the naturally occurring impurity minerals in coal cannot always be eliminated from the carbonaceous product, and these remaining components do not contribute to the formation of porous structures.^[^ ^56^ ^]^ The introduction of heteroatoms such as nitrogen either via in‐situ or ex‐situ operations will extend the distance between the carbon planes.^[^ ^57^ ^]^

Petroleum coke is another waste product of the heavy oil upgrading process that, due to its low price and high carbon concentration, has potential as a carbon precursor for producing porous carbons. In addition to the major component of carbon, petroleum coke also contains volatile compounds such as heavy hydrocarbons. Because volatiles evaporate during heating, the composition of petroleum coke after carbonization can change depending on the temperature of carbonization.^[^ ^4^, ^58^ ^]^ At the moment, coal‐based precursors are one of the major resources for commercial production of porous carbons, but these raw materials alone cannot meet the growing demand for porous carbon across numerous industries. As a result, other natural precursors should also be investigated, which can provide low‐cost and cost‐effective options to fulfill the predicted demand.^[^ ^59^ ^]^

### 2.2 Biomass

**Figure 5:**
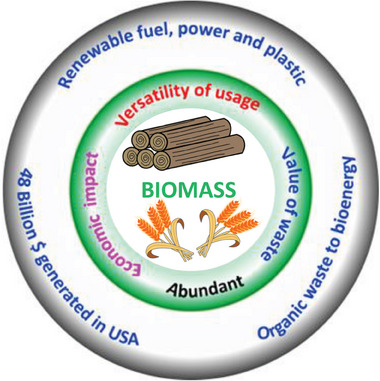
General schematic of the advantages of using biomass.

Biomass resources refer to all materials obtained from plants and animals and are a top priority among natural precursors owing to low cost, abundance, sustainability, and environmental friendliness (**Figure** **5**).^[^ ^49^, ^60^ ^]^ They are readily available and exhibit a natural source of organic carbon that can not only be utilized for the synthesis of porous carbons^[^ ^61^ ^]^ but also as an organic feedstock to produce heat, bio‐based commodities, energy, fuels, and other value‐added byproducts.^[^ ^62^ ^]^ Biomass typically has a heterogeneous structure with a complex chemical content, both of which have a significant impact on the microstructure development during synthesis steps.^[^ ^9^, ^63^ ^]^ In general, biomass is divided into three types: raw biomass (Lignocelluloses, harvests, and plants), waste biomass (Industrial solid waste and wastewater), and agricultural wastes.^[^ ^64^ ^]^ From a chemical point of view, biomass is primarily composed of carbon, oxygen, hydrogen, and nitrogen. The structure of biomass is not confined to these components only but also to some other elements like sulfur, calcium, and magnesium which are present in rather trace amounts.^[^ ^65^ ^]^ Cellulose (C_6_H_10_O_5_)_n_, hemicellulose (C_5_H_8_O_4_)_n_, and lignin (C_9_H_10_O_3_ (OCH_3_)_0.9‐1.7_ or C_18_H_13_N_3_Na_2_O_8_S_2_) are the three principal structural components observed in lignocellulosic biomass with approximately 30–55%, 20–40%, and 2–15% contents, respectively, plus a small fraction of extractives such as ash, flavonoids, waxes, proteins, and chlorophylls.^[^ ^66^ ^]^ It should be mentioned that the most abundant renewable natural biopolymer on the planet is cellulose. It may be found in a wide range of biological systems, including plants, animals, and microorganisms. Poly (β_1, 4_ ‐linked glucose or β−1 → 4‐D‐glucopyranose), which is made up entirely of linearly organized anhydroglucose units, is known as cellulose. The existence of intramolecular and intermolecular hydrogen bonding between OH groups causes the chains to be strong, linear, equal, and crystalline.^[^ ^67^ ^]^

Hemicellulose, present in secondary cell walls, is the second most prevalent ingredient in most biomass‐based compounds in terms of percentage composition. Hemicelluloses are a group of complex biopolymers that have a β‐(1→4) backbone of neutral sugars like glucose and mannose and are made up of multiple heteropolymerized saccharides, similar to cellulose. Due to their randomly amorphous state and low molecular weight, hemicellulose is very easy to decompose.^[^ ^68^ ^]^ Lignin is a high‐molecular‐weight complex organic polymer generated by the integration of three phenyl‐propanoid units. Lignin molecules have a strong polarity due to the presence of functional groups such as carboxyl groups on their surfaces. Lignin has been found as the primary component in lignocellulosic biomass responsible for the CO_2_ adsorption process among the three fractions of lignocellulosic materials.^[^ ^59^, ^69^ ^]^ The usual functional groups based on IR spectra reveal that lignin may be rich in methoxyl‐O‐CH_3_, C‐O‐C stretching, and C═C stretching (aromatic ring). Although cellulose had the maximum IR absorbance of OH and C–O compounds, hemicellulose had more C═O compounds.^[^ ^70^ ^]^ The most important aspect of these three main components is the variability in their content in different precursors. This is further dependent on various factors such as plant species, resources, climatic conditions, and the age of plants. Therefore, the best precursor can be selected by examining all the conditions to obtain porous carbons with desired porosity.^[^ ^71^ ^]^

## 3 Synthesis

**Figure 6:**
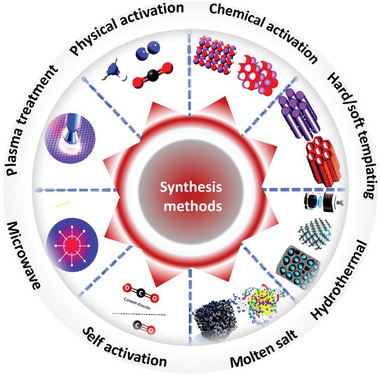
A variety of porous carbon synthesis methods, from traditional to advanced methods.

Porous carbon synthesis entails the pyrolysis of various types of raw natural precursors using various techniques and their functionalization with diverse activators. Porous carbon can be synthesized in a variety of ways (**Figure** **6**), which are categorized into two classifications: traditional and advanced. Physical and chemical activation, hydrothermal, hard‐soft templates, and direct pyrolysis are all examples of traditional approaches.^[^ ^72^ ^]^ Advanced approaches include technologies like self‐activation, microwaves, and plasma. Various porous carbons with different textural parameters can also be prepared by varying the nature of the precursors, heating conditions, activating reagents, activation steps or the heating environment.^[^ ^21^, ^22^, ^25^, ^29^, ^73^ ^]^

The carbonization cum activation procedure is the most extensively utilized method for the synthesis of porous carbons. This process requires the carbonization of the precursor for the formation of non‐porous structure and then activation with activators (e.g., potassium (KOH), zinc chloride (ZnCl_2_), or sodium hydroxide (NaOH)) to develop micro and mesoporous structures.^[^ ^74^ ^]^ During the carbonization, non‐carbon components such as H and O are eliminated from the precursors in gaseous forms, and the free atoms of elementary carbon are clustered into organized crystallographic form, known as elementary graphite crystallites. The crystallites' reciprocal arrangement is irregular, resulting in open interstices between them. As a result of the deposition of tarry compounds, the open interstices existing in the char get filled or at least partially blocked by disordered “amorphous” carbon during carbonization. However, the adsorption capability of this carbonized precursor is quite low. By eliminating tarry products using activation systems, the sizes of the pores generated during the carbonization process are increased, and additional pores are created, resulting in the production of a well‐developed and easily accessible pore structure with a high interior specific surface area (S_BET_).^[^ ^75^ ^]^ These processes may provide uniform hierarchical texture, but they generally necessitate lengthy operations, hazardous corrosive chemicals, and a huge amount of alkalinity, limiting their industrial applicability.

Since porous carbons with inherent functional groups show low or moderate amounts of gas sorption, there are complementary approaches for increasing functional groups and effective elements via doping to enhance their gas sorption capability.^[^ ^76^ ^]^ Heteroatoms can be doped into porous carbons via either in‐situ or ex‐situ operations. The ex‐situ procedure is a preferred option for surface functionalization whereas in‐situ doping results in a more homogeneous and stable heteroatom distribution and is a preferred method for heteroatom doping. Nonetheless, currently, available heteroatom precursors typically need costly monomer activators, convoluted chemical synthesis pathways, and hazardous experimental procedures.^[^ ^77^ ^]^ However, doping of amorphous mesoporous carbons or carbon precursors with heteroatoms results in lower S_BET_ than their undoped porous carbon counterparts. Nitrogen is one of the most popular elements for heteroatom doping in porous carbon because of its comparable atomic sizes. Urea, ammonia, amines, melamine, and other nitrogen sources are used in the nitrogen doping process. For instance, ethylenediamine tetraacetic acid (EDTA) salt has recently been shown to be useful as a nitrogen‐rich carbon precursor. These N‐doped porous carbons with high S_BET_ have been made quickly and easily using one‐step pyrolysis. Because of the low carbon concentration in the salts and the abrupt expansion during pyrolysis, direct pyrolysis of organic salts generally results in a low carbonization yield.^[^ ^78^ ^]^

Sulfur is the second most important element in the doping process, which usually penetrates the synthesized porous carbon structure independently or integrated with the nitrogen element. However, the presence of sulfur in the porous structure requires the provision of expensive sulfur precursors and complex equipment. Given the drawbacks of each of these technologies, selecting an appropriate strategy such as a low‐cost and simple synthesis path is highly critical which may lead to the industrial production of heteroatom‐doped porous carbon. In the following, we will investigate each of these synthesis methods and compare the adsorption results on the final porous carbon product. For example, the sulfur/carbon combinations can be obtained at 150 °C at which the melted sublimed sulfur diffuses into the pores of porous carbons prepared in a sealed chamber. At 300 °C, the sulfur coating on the outer surface of porous carbon can be evaporated.^[^ ^79^ ^]^

### 3.1 Physical and Chemical Activation

**Figure 7:**
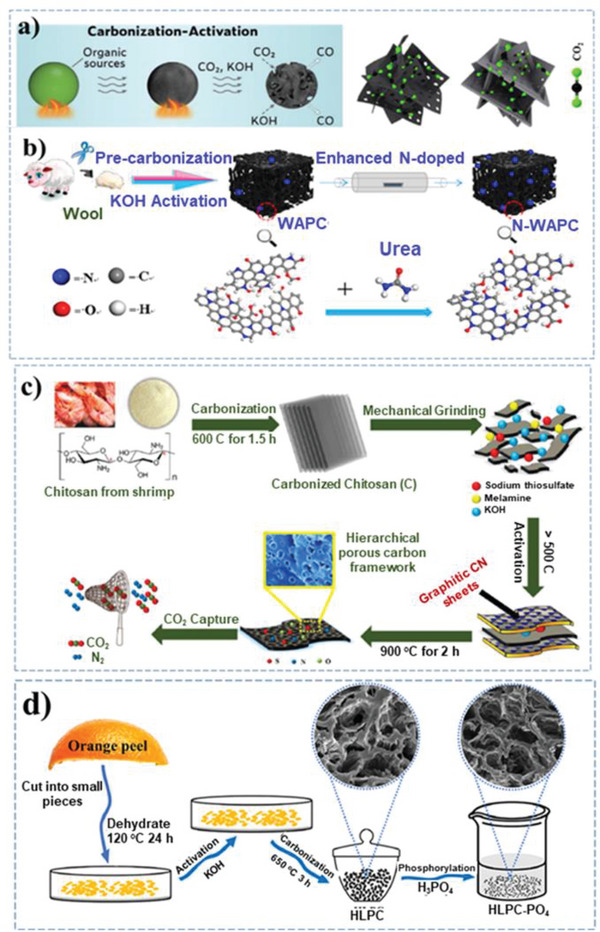
Various methods of using KOH chemical reagent to achieve porous carbon. a) schematic of physical and chemical activation; b) By pre‐carbonization, mechanical grinding, and adding urea, Reproduced with permission^[^ ^86^ ^]^ Copyright 2020 Wiley; c) By adding Melamine and Sodium thiosulfate, Reproduced with permission.^[^ ^87^ ^]^ Copyright 2021, Elsevier; and d) Using pre‐activation+phosphorylation, Reproduced with permission.^[^ ^88^ ^]^ Copyright 2021, Elsevier.

Physical and chemical activations are the most common ways of producing porous carbons. The schematic of these methods, from the beginning of the synthesis to the adsorption of CO_2_ on the porosity resulting from the synthesis, is shown in **Figure** **7**a. Chemical activation uses acidic, alkaline, and salt materials whereas physical activation methods use different types of gases including steam and carbon dioxide. Carbonization of the precursor in an inert environment followed by the activation of the resultant char with the activating agents such as steam, CO_2_, N_2_, or air are examples of physical activation.^[^ ^80^ ^]^ In an inert environment and at moderate temperatures (300‐600 °C), the carbonization stage consists of a pyrolysis method in which volatile chemicals are emitted owing to multiple complicated, competing, and successive reactions leading to the accomplishment of carbon content. The precursor is then heat‐treated at high temperatures (750–1200 °C) in the presence of oxidizing agents in the second phase of the physical activation process.^[^ ^81^ ^]^ The reactions occurring during activation include the following:

(1) $$C+H_{2}O\to \mathrm{CO}+H_{2}$$

(2) $$C+{\mathrm{CO}}_{2}\to 2\mathrm{CO}$$

For instance, Ogungbenro et al. reported that the porous carbon derived from the seeds by activation under CO_2_ atmosphere in the temperature range of 600–900  °C can be used as potential adsorbents for CO_2_ uptake.^[^ ^82^ ^]^ However, physical activation suffers from several disadvantages such as the low porous carbon yield, high temperature of activation, high activation time, and poor porosity.

**Figure 8:**
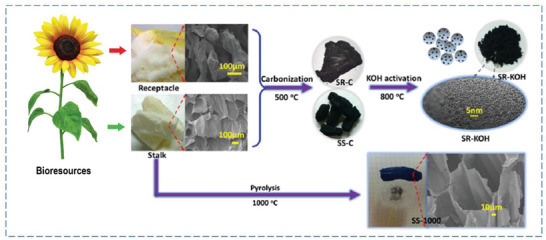
Spongy flesh from receptacle and stalk of sunflowers as precursors to synthesize porous carbons with and without KOH activation in argon atmosphere, Reproduced with permission^[^ ^50b^ ^]^ Copyright 2019, Elsevier.

In recent years, due to its great effectiveness, chemical activation utilizing chemicals (acid, base, salt) such as KOH, NaOH, ZnCl_2_, phosphoric acid (H_3_PO_4_), formamide, potassium carbonate (K_2_CO_3_), and sodium carbonate (Na_2_CO_3_) has been extensively developed to impregnate C‐rich precursor. Porous carbon produced by chemical activation typically has a S_BET_ of 800–3100 m^2^.g^−1^ and is mostly composed of ultra micropores, micropores, and narrow mesopores (0.5–4 nm).^[^ ^83^ ^]^ During the chemical activation process, the activating agents are either added to the biomass precursors or reacted with the carbonized biomass. This process significantly reduces the volatiles and further inhibits particle shrinkage, resulting in a high yield of porous carbon after the removal of the activating agent.^[^ ^84^ ^]^ Many porous carbons with different textural properties and functionalization for CO_2_ sorption can be prepared with the activation by KOH as shown in Figure 7b–d. As can be seen in Figure 7, porous carbon can be obtained by using operations such as pre‐carbonization, mechanical grinding, or integration of KOH with other activators such as urea (Figure 7b), melamine and sodium thiosulfate (Figure 7c), and H_3_PO_4_ (Figure 7d) or can be used exclusively (**Figure** **8**). The use of other activators or nitrogen‐rich reagents (melamine/urea) not only leads to the improvement of the porosity but also allows dope useful elements onto the carbon framework to adsorb more CO_2_.

Through the activation with KOH using different KOH/C ratios (1‐5), mostly micropores are formed upon the removal of chemical components from the char and the emission of gaseous products.^[^ ^85^ ^]^ At first, primitive pores are formed up to 500 °C and are developed further in the activation stage (600 °C–900 °C). KOH activation occurs through various steps. Below 600 °C,

the following reaction occurs between KOH and carbon source, forming potassium carbonate (etching agent), potassium, and hydrogen gas as described in Equation (3).

(3) $$2C+6\mathrm{KOH}\to 2K_{2}{\mathrm{CO}}_{3}+2K+3H_{2}$$

The formed K then reacts with KOH to form K_2_O which is another etching agent. The reaction occurs between K_2_O and CO_2_ below 400 °C also forms potassium carbonate.

(4) $$K_{2}O+{\mathrm{CO}}_{2}\to K_{2}{\mathrm{CO}}_{3}$$

At or above 700 °C, the formed etching agents then react with carbon to produce the carbon monoxide and the porosity (Equations 5 and 6).^[^ ^86a^ ^]^

(5) $$K_{2}{\mathrm{CO}}_{3}+2C\to 2K+3\mathrm{CO}$$

(6) $$K_{2}O+C\to 2K+\mathrm{CO}$$

Following this mechanism, many porous carbons have been derived. For example, as shown in Figure 7d, Sun et al. activated the orange peel by KOH as a precursor for the synthesis of charcoal and then developed a phosphorylated honeycomb porous carbon using H_3_PO_4_. The S_BET_ of porous carbon and porous carbon‐PO_4_ was 432.3 m^2^ g^−1^ and 378.5 m^2^ g^−1^, respectively. It seems that the use of reagents for the doping of elements has led to a decrease in the S_BET._^[^ ^88^ ^]^ According to Figure 8, Sun et al. developed porous carbon from spongy flesh and stalk with and without KOH activation. The final product possessed S_BET_ up to 3072 m^2^ g^−1^, and light density (0.033 g cm^−3^). It seems that in spite of achieving a high S_BET_ during the exclusive use of KOH, the CO_2_ adsorption capacity of the material at 273 k was only 4.52 mmol g^−1^.^[^ ^50b^ ^]^ This may be attributed to the lack of functional elements in the materials. Even though some porous carbons employing KOH‐based chemical activation have S_BET_ of over 3500 m^2^ g^–1^, the main disadvantage of KOH is its high causticity, which causes safety problems and device damage at high temperatures.^[^ ^89^ ^]^

In some cases, the integration of activation methods with other methods or other activators leads to improved product quality. Hao et al. reported the fabrication of an interconnected carbon nanosheet with abundant micro/meso holes from ginkgo leaves using the hydrothermal treatment and KOH activation. Due to the intense interactions between the rich biological components of ginkgo leaves including proteins and sulfur acids during the preparation procedure, a very high doping of S (8.245 wt%) and N has been attained.^[^ ^90^ ^]^ The presence of N and S in the carbon in a large quantity significantly improved the energy storage performance of the materials in both supercapacitors (364 F g^−1^ at 0.5 A g^−1^) and sodium ion battery (200 mA h g^−1^ at 0.2 A g^−1^). Besides, Zhang et al. developed a porous carbon from a black locust that has a S_BET_ of 2511 m^2^.g^−1^ and high CO_2_ sorption of 7.19 mmol g^−1^ at 0 °C under 1 bar after chemical activation with KOH and surface modification with ammonia solution.^[^ ^32a^ ^]^ In addition, the prepared materials showed excellent selectivity for CO_2_/N_2_ and fast adsorption kinetics due to the presence of basic nitrogen‐containing functionalities in the porous carbons with high specific surface area. H_3_PO_4_ has lately become the preferred reagent for chemical activation owing to its less corrosiveness and the avoidance of excessive washing and offers porous carbons with both micropores and mesopores.^[^ ^91^ ^]^ H_3_PO_4_ can play a role as an activator near 550 °C, selectively oxidizing carbon slowly and producing narrow pores. On the other hand, ZnCl_2_ is easily evaporated and degraded at 450 °C, leading the carbon skeleton to lose its protection and thereby rapidly burn out. As ZnCl_2_ has a lower starting reaction temperature than H_3_PO_4_, its activation kinetics is faster.^[^ ^92^ ^]^ Nonetheless, these conventional reagents are extremely corrosive and carry hidden hazards (e.g., flame and explosion), making the hazardous process to the environment and risking the safety of workers in industrial facilities.

Owing to their great pore tailoring performance and low corrosiveness, potassium salts such as KHCO_3_ (potassium bicarbonate) and K_2_C_2_O_4_ (potassium oxalate) as reagents have drawn increased interest in solving these concerns.^[^ ^93^ ^]^ The characteristics of the potassium salt can influence the pyrolysis (together with carbonization) of biomass, as well as the physico‐chemical properties of porous carbon.^[^ ^94^ ^]^ When various potassium oxy‐salts were utilized, the porosity and shape of glucose‐derived porous carbons differed significantly. Deng et al. described a simple leavening process using KHCO_3_ as a reagent to produce hierarchical porous carbon with numerous macropores from various natural precursors and discovered that pyrolysis releases a substantial quantity of H_2_O and CO_2_.^[^ ^95^ ^]^ Although KHCO_3_ is successful for most natural precursors, lignin‐rich precursors have limited pore tailoring due to restricted contact at the char‐reagent interface.^[^ ^96^ ^]^ Also, sodium amide (NaNH_2_) is another interesting activating agent and has recently been discovered to act as both chemical activation and N‐doping agents in a single‐step synthesis at relatively moderate temperatures. Because of its high basicity and nucleophilicity, NaNH_2_ can effectively activate the C‐rich precursor at temperatures between 400 and 500 °C, compared to other reagents. Lower activation temperatures can successfully prevent equipment corrosion while also allowing for energy savings and micropore creation. However, it is not a very practical chemical reagent because it is highly flammable when it is in contact with water.^[^ ^97^ ^]^;

In another research, Geng et al. presented a novel synthesis of N‐doped microporous carbon derived from corncob using an ammonia gas (NH_3_)‐assisted activation process in which NH_3_ serves as both the activating reagent and the N source. To begin with, corncob was heated to 400 °C under N_2_ atmosphere to obtain char. Then, the N_2_ was replaced with NH_3_, and the char was heated at 400–800 °C under NH_3_. The first step involves activation and the second step involves doping.^[^ ^98^ ^]^ Through this process, both activation and N‐doping can be obtained without the addition of any conventional chemical activating agent.

Chemical activation provides benefits of economic cost, ease of operation, short reaction time, lower synthesis temperature, and excellent yields compared to physical activation.^[^ ^99^ ^]^ Chemically activated porous carbon also has a lot of pores and a wide pore size distribution (0.5‐10 nm). Nevertheless, chemical reagents have pollution issues originating from acid pickling (to remove pollutants generated during the activation process).^[^ ^100^ ^]^ In addition to the above methods, the C‐rich precursor or char is penetrated with reagents and heated in a chamber with an oxidant flow in the physico‐chemical activation process, which uses both physical and chemical processes. This approach is frequently utilized when the reagent used in the activation process cannot be adequately removed by acid‐pickling and would otherwise cause pore blockage. However, apart from the high‐temperature requirements, the need for a two‐step processing, prolonged process time, and a compromised yield of porous carbon make this method costly.^[^ ^101^ ^]^

### 3.2 Hydrothermal Carbonization (HTC)

**Figure 9:**
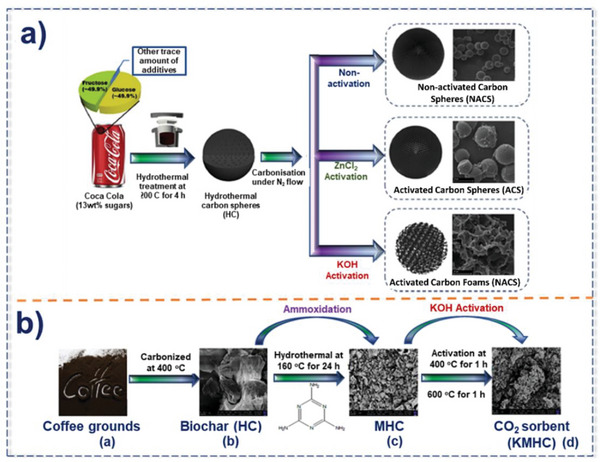
Various methods of porous carbon synthesis using HTC, a) Using ZnCl_2_ and KOH after hydrothermal. Reproduced with permission^[^ ^102^ ^]^ Copyright 2017, Elsevier, and b) Using ammoxidation and KOH, Reproduced with permission^[^ ^103^ ^]^ Copyright 2018, Elsevier.

HTC is another method for the synthesis of precursors via chemical reactions in a sealed and heated solution (150‐250 °C) and high pressure in stainless steel autoclave as shown in **Figure** **9**a,b. To improve surface functionality, nitrogen and sulfur precursors can be incorporated through the HTC process. Therefore, the final product (Hydro‐char) can then be used in two ways: (1) direct carbonization without using any reagents or (2) activation by reagents (KOH, ZnCl_2_) followed by calcination to create functional surface –COOH groups, which could then be ion‐exchanged with K to generate –COOK groups.^[^ ^12^, ^61^ ^]^ In (1), the prepared sample has a low S_BET_ and insignificant functional groups.

**Figure 10:**
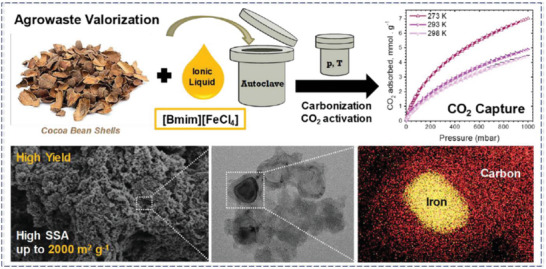
Cocoa bean shells derived porous carbon with high S_BET_ (2000 m^2^. g^−1^) synthesized by [Bmim] [FeCl_4_] and CO_2_ activation. As shown, the synthesized porous carbon has a remarkable CO_2_ capture as high as 4.4 mmol g^−1^ at 25 °C under 1 bar, Reproduced with permission^[^ ^105^ ^]^ Copyright 2020, Royal Society of Chemistry.

The HTC can be carried out in various solvents such as ethanol, and dilute acids.^[^ ^47^ ^]^ By HTC of biomass, Sevilla et al. created porous carbons with high surface area and observed a CO_2_ adsorption of 4.8 mmol g^−1^ at 1 bar and 25 °C.^[^ ^24^, ^104^ ^]^ Recently, Cibien et al. reported the ionothermal carbonization (ITC) incorporated with HTC method for an agro‐waste precursor, cocoa bean shells, used as a model in [Bmim] [FeCl_4_] as depicted in **Figure** **10**. The coordination of [FeCl_4_]^−^ to the O atoms of precursor and ionochars stabilize oxygenated groups of porous carbon and provide enhanced yields which favor the generation of microporous structure.^[^ ^105^ ^]^

### 3.3 Molten Salt Synthesis (MSS)

**Figure 11:**
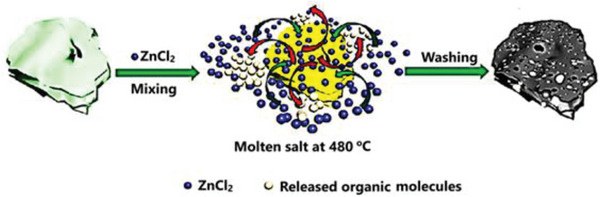
Molten salt synthesis at 480 °C using ZnCl_2_ Reproduced with permission^[^ ^80^ ^]^ Copyright 2011 Royal Society of Chemistry.

The molten salt synthesis (MSS), also known as the salt templating method, is based on the use of a low melting point salt or a mixture of salts as the molten medium for the targeted synthesis as displayed in **Figure** **11**. The chlorides, sulfates, carbonates, and hydroxides such as LiCl‐ZnCl_2_, NaCl‐ZnCl_2_, and KCl‐ZnCl_2_ are the most prevalent salts used for MSS due to their low‐cost and high availability. The salt templating technique offers a useful flux environment with the solubility and diffusivity needed for solid‐phase reactions, lowering the synthesis temperature significantly.^[^ ^80^, ^106^ ^]^ At high temperatures, C‐rich precursors were condensed and structured in the presence of molten salt. A solvent can alternatively be used to dissolve the carbon precursor and the salt. The solvent evaporates during condensation, which can be accomplished by freeze‐drying or heating, and the salt clusters or heating results serve as templates for porosity formation.^[^ ^107^ ^]^ The template evaporation and pore formation in the skeleton are caused by the pyrolysis of the condensed products. The compounds of the heating process can be used as a chemical agent to generate new porosities. These compounds react with the carbon‐containing precursor and cause porosity in the carbon structure through an etching process. With correct salt choice and process parameters such as pyrolysis duration and temperature, as well as the type and ratio of the salt and the carbon precursor, it is easy to adjust the pore size distribution.^[^ ^108^ ^]^

### 3.4 Self‐Activation

**Figure 12:**
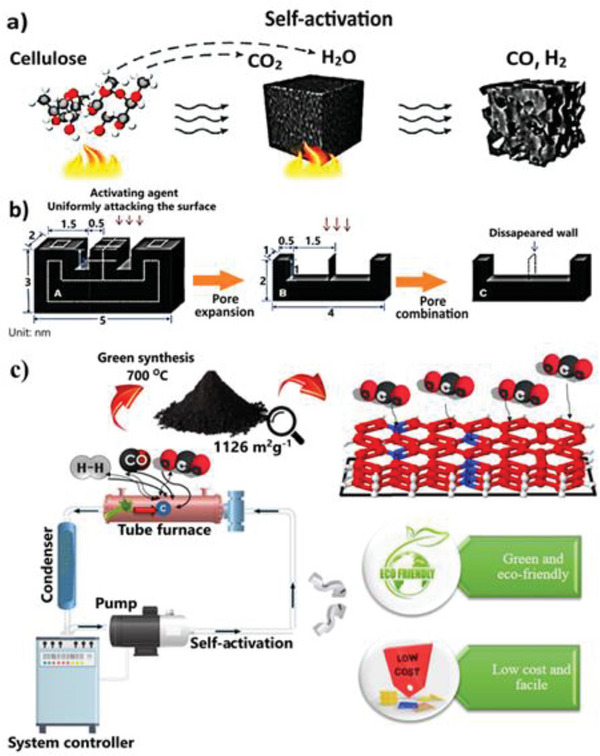
Schematic of self‐activation method. a) General schematic of traditional self‐activation method under inert gases or through self catalytic‐metal. Reproduced with permission^[^ ^86a^ ^]^ Copyright 2020 Wiley; b) Mechanism of self‐activation method includes pore expansion and pore combination Reproduced with permission^[^ ^112^ ^]^ Copyright 2015 Royal Society of Chemistry; c) New green self‐activation method using a pump and gas flows in a closed cycle Reproduced with permission^[^ ^114^ ^]^ Copyright 2022 Elsevier.

One of the most effective ways of producing porous carbon is self‐activation of starting precursors. Organic salts have been the subject of most studies for their self‐activation. The self‐activation approach is, in fact, a subset of the direct carbonization method. In certain situations, before the procedure, the materials are treated with reagents to dope heteroatoms such as sulfur and nitrogen into the structure.^[^ ^28^ ^]^ As a result, the phrase “self‐activation” does not accurately describe this synthesis approach. This approach is classified as 1) self‐activation in the presence of inert gases (**Figure** **12**a) and 2) green self‐activation according to Figure 12c. However, the first type is also known as direct pyrolysis, which occurs in a tube furnace at 600–1100 °C for 1–3 h in flowing N_2_/Ar or with metal as a catalyst (template).^[^ ^109^ ^]^ In the first category, some interest has been shown in using gases generated during the pyrolysis process or non‐additional metals in biomass to activate the carbon source. Unlike the previously described traditional activation methods, no extra activating chemicals are required during the self‐activating process, making this approach more energy‐efficient and ecologically friendly.^[^ ^110^ ^]^ The primary pyrolysis gases such as H_2_O, CO_2_, and CH_4_/C_2_H_6_ are released during the activation process. The gases such as CO_2_ and H_2_O, act as activating agents and make up a major fraction of gases, evolved at 300 °C as observed in GC/MS results.^[^ ^111^ ^]^ The initial phase in pore creation occurred between 300 and 500 °C when a considerable amount of pyrolysis gases escaped from channels created by precursors. The second stage occurred at a high temperature of 700–1000 °C, during which high pressure in the sealed reactor was created. Gasification interactions with carbon pushed pyrolysis gases into the channels under auto‐generated pressure, leading to the creation of micropores (Figure 12b).^[^ ^112^ ^]^ However, a large amount of the gases is emitted into the environment during this process and therefore, improved methods are required.

Using a one‐step self‐activating system, Banna et al. developed a green, low‐cost, and simple technique for producing green porous carbon with mesopore dominance from a walnut shell, celery, and lemon waste as precursors (Figure 12c).^[^ ^113^ ^]^ The suggested method is based on a closed‐loop carbonization system that utilizes the reagent gases breakdown from biomass. The gases created during the pyrolysis of the feeding biomass are cycled in a closed‐loop channel with the help of an air pump located in the system. The activation process is carried out with the assistance of these gases. Consequently, no further reagent is needed. Along the course of the gas, a condenser collects and converts a portion of the exhaust gases to liquid. It is possible to accomplish the general process of self‐activation, which is manifested in the following Equations ((7), (8), (9), (10), (11)):^[^ ^114^ ^]^

(7) $$H_{2}O_{\left(l\right)}\to H_{2}O_{\left(g\right)}$$

(8) $$C_{\left(s\right)}+H_{2}O_{\left(g\right)}\to {\mathrm{CO}}_{\left(g\right)}+H_{2\left(g\right)}$$

(9) $$2C_{\left(s\right)}+O_{2\left(g\right)}\to 2{\mathrm{CO}}_{\left(g\right)}$$

(10) $${\mathrm{CO}}_{2\left(g\right)}+H_{2\left(g\right)}\to {\mathrm{CO}}_{\left(g\right)}+H_{2}O_{\left(g\right)}$$

(11) $$C_{\left(s\right)}+{\mathrm{CO}}_{2\left(g\right)}\to 2{\mathrm{CO}}_{\left(g\right)}$$

At low temperatures, moisture is progressively evaporated and transformed to vapor. Then, the activation occurs slowly at temperatures exceeding 700 °C, and the gases created during the pyrolysis process with continuous rotation in the circulatory system play an important part in the self‐activation process. The typical Gibbs free energy change of the Boudouard reaction (Reaction 9) becomes negative as the activation temperature rises above ≥700 °C. As a result, CO_2_ and steam (H_2_O) created by cellulose degradation gases may react with carbon atoms and permeate into the core of the carbon substance, increasing porosity.^[^ ^115^ ^]^

### 3.5 Hard‐Soft Templating

Slow mass transport and poor pore accessibility afflict conventional porous carbon materials with poorly organized pore structure. Furthermore, carbon compounds generated by direct carbonization of various C‐rich sources do not often contain a mesoporous architecture. As a result, pore‐forming agents must be introduced throughout the carbonization process to develop mesopores. Also, the capability to regulate the porosity distribution and order (periodically ordered mesoporous carbon structure (OMC)) during pyrolysis is the most significant property of a suitable synthesis for high gas sorption.^[^ ^116^ ^]^

**Figure 13:**
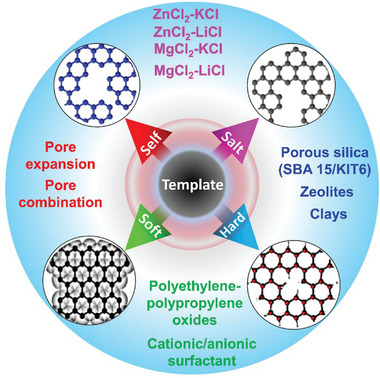
Schematic illustration of the porous carbon synthesis methods using different types of templates.

Therefore, new approaches such as hard and soft templating to overcome the mentioned problems faced by self and salt templating have been developed (**Figure** **13**). In these methods, C‐rich precursors are polymerized using various hard or soft templates such as porous silica, zeolites, and clays (hard templating) or amphiphilic blocks such as polyethylene‐polypropylene oxides, cationic or anionic surfactants (soft templating). Following polymerization, the templates are removed from the structure by chemical treatment (hot alkaline solution or very toxic hydrofluoric acid) or calcination (carbonization).^[^ ^117^ ^]^

The key benefits of templating methods include the ability to tune pore size and structure, as well as the ability to synthesize OMCs. However, the time‐consuming and expensive processes that result from the requirement of employing, typically, expensive templates, as well as eliminating the templates after the synthesis operations, are disadvantages of these approaches. There have been reports on the synthesis of OMCs with a variety of characteristics, including shape, pore symmetry, and pore size.^[^ ^118^ ^]^ Though templates, mainly mesoporous or microporous silicas, with different structures and morphologies, can provide reasonably exact control over the outcome, various caustic and hazardous chemicals (e.g., HF, NH_4_HF) used in the manufacturing process impose a significant environmental impact.^[^ ^119^ ^]^ Furthermore, the post‐disposal of silica templates makes it time‐consuming, wasteful of components, yet cost‐effective. In the case of soft templating process, amphiphilic surfactants are mainly used as templates. These templates can be removed by simple pyrolysis. Therefore, soft‐templating, also known as direct synthesis, has become a more popular method of producing OMCs using various raw materials. Although natural wastes and their derivatives are abundant, nontoxic, and low‐cost natural renewable resources with large‐scale manufacturing potential, the controlled synthesis of OMCs from natural wastes due to a lack of knowledge of the process is still in its early stages. Despite recent efforts, natural precursors are still limited to a small number of carbon sources with specified properties. For example, low polymerization temperature and great bonding affinity for soft templates with carbon sources are the major disadvantages for the creation of a wide range of OMCs.^[^ ^120^ ^]^

### 3.6 Microwave Heating

**Figure 14:**
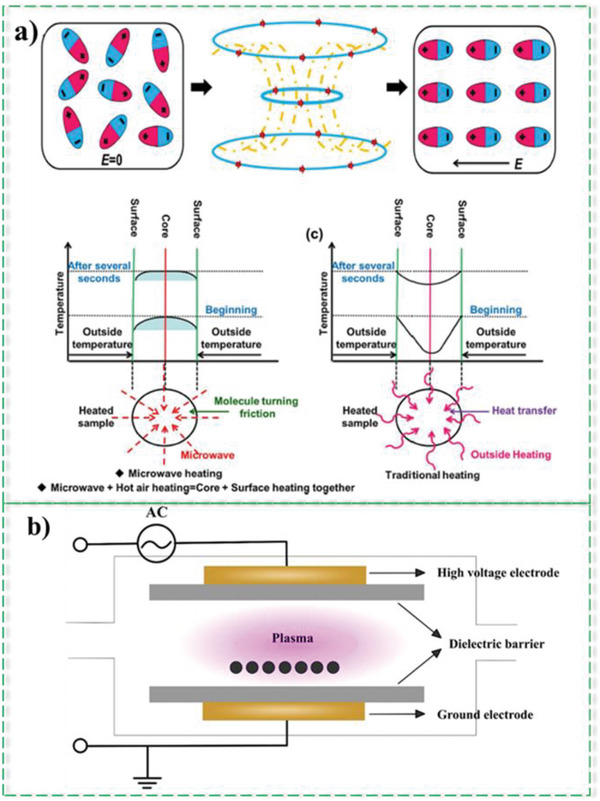
a) General schematic of the microwave method: Investigating the differences between the type of heat transfer in traditional methods and the microwave method, Reproduced under the term of the Creative Commons CC BY license.^[^ ^126^ ^]^ Copyright 2015, The Authors, published by SpringerNature. b) Schematic of plasma method for modifying porous carbon surface.

CO_2_ and steam are two activating agents often used in physical activation. Although these are environmentally benign, the activation process has some deficiencies.^[^ ^121^ ^]^ Traditional heating techniques heat the sample from the outside, which causes surface heating and cannot guarantee uniform heating of samples of various shapes and dimensions. Thus, a temperature gradient would be created in the inner and outer parts of the sample, which hinders the gas release process.^[^ ^122^ ^]^ As a result, the activation process requires more time and higher temperatures, resulting in less amount of the product and higher energy consumption. Recently, microwave heating has been considerably explored as a potential alternative to conventional activation methods, as shown in **Figure** **14**a. Microwave is electromagnetic radiation whose wavelength and frequency depending on its applications, vary from 1 mm to 1 m, and 0.3 to 300 GHz, respectively. MW heating is a type of dielectric heating produced at specific frequencies.^[^ ^123^ ^]^ In this method, the energy is not transferred by convention or conduction, but the heat is generated in a different way. By applying a high‐frequency voltage, the electric field component of MW, which has a specific direction, makes polar molecules rotate, and the permanent dipole molecules orient in the opposite direction of the electric field. The dipole rotation and ionic conduction produce heat inside the particles.^[^ ^124^ ^]^ As a result, in comparison with conventional methods, microwave heating provides high‐rated heating that significantly decreases the activation time and increases energy efficiency. It results in more carbon yield, enhanced AC quality, and lower production of waste and hazardous materials. These characteristics make MW heating an environmentally friendly technique. Other advantages of this method are that the precursor and the heating source are not directly in contact, the equipment required for the procedure is smaller, and there is more control over the process.^[^ ^125^ ^]^ Greater induced polarity increases MW influence, which provides MW heating with characteristics such as uniformity, selectivity, and being volumetric.^[^ ^123^ ^]^ In one of the reports, Ania et al.^[^ ^124b^ ^]^ demonstrated that the heat treatment of the activated carbon with MW and thermal heating has a huge influence on the final porous structure of the materials. When MW was used, the regeneration time was significantly reduced without much affecting the microporous structure. In another interesting report, Yang et al.^[^ ^124a^ ^]^ demonstrated the preparation of porous carbon from coconut shell using physical activating agents such as CO_2_ and steam using microwave heating. MW heating significantly reduced the activation time to achieve the porous carbon with much higher high specific surface areas than those of the samples prepared with normal thermal heating, revealing the unique power of MW heating.

### 3.7 Plasma Treatment

During the past two decades, plasma has been extensively researched and applied in various fields. Plasma treatment is an energy‐saving and eco‐friendly method and has been proven to be a novel approach for modifying the morphology and surface chemistry of materials conveniently.

It can create enormously active species such as atomic excited states, ions, and electrons that could mediate surface chemistry. Heteroatom‐doped carbon materials with tunable pore size distribution can be achieved at low temperatures by plasma.^[^ ^127^ ^]^ Recently, the plasma irradiation method has gained increased attention for surface modification of porous carbons to provide specific applications. In comparison with conventional modification methods, plasma irradiation causes much less damage to the textural properties of activated carbon.^[^ ^128^ ^]^ According to Figure 14b, irradiation of plasma in different gas environments leads to the introduction of different functional groups on the surface of activated carbon through oxidation, reduction, or inactive reactions. Since there is no need for any chemical solution in plasma treatment, it is considered to be a simpler method compared to chemical treatments.^[^ ^129^ ^]^

Non‐thermal plasma treatment can react with porous carbon and add active species onto its surface. Wu et al. prepared O/N co‐doped porous carbon for CO_2_ adsorption by HTC of natural precursors and non‐thermal plasma treatment. A dielectric barrier discharge reactor with an alternating current of 220 V was used to produce plasma irradiation. The activated carbon was treated under non‐thermal plasma in the air environment. The DFT calculations demonstrate that oxygen and nitrogen functional groups could rapidly be introduced to porous carbon through air plasma treatment and plasma does not cause a considerable change in the textural characterizations. The prepared heteroatom‐doped porous carbon has a larger specific surface area and microporous structure and showed a high adsorption rate that resulted in 37.42 mg.g^−1^ CO_2_ capture.^[^ ^130^ ^]^

### 3.8 Joule Heating

The Joule heating method relies on the principle of resistive heating and can be used to convert carbon‐rich materials into specific single crystalline (with negligible defects) morphologies such as carbon nanotubes and graphene. The passage of electric current through carbon gives rise to Joule heating due to the resistance which results in controlled thermal decomposition of the carbon and produces the desired specific structure. In the initial research endeavours, Iijima pioneered the creation of graphitic carbon needle‐like structures through the utilization of the arc discharge evaporation method.^[^ ^131^ ^]^ These micro‐needles were synthesized at the negative end of a carbon electrode within a vessel filled with argon gas. Hu et al. employed Joule heating to covert a graphene oxide/lignin film into highly conductive crystalline carbon.^[^ ^132^ ^]^ Temperatures nearing 2500 K were employed to eliminate defects and catalyze the graphitization process, resulting in the creation of graphitic carbon with a short‐range ordered structure. With a notably enhanced conductivity (4550 S cm^−1^), this form of graphitic carbon holds significant potential for applications in various energy‐related fields and beyond. The same research team took the concept of Joule heating a step further by applying it to the creation of carbon‐coated nickel nanoparticles on a matrix of reduced graphene oxide, which were subsequently harnessed as a catalyst for H_2_O_2_ fuel.^[^ ^133^ ^]^ To achieve this, they subjected the materials to temperatures reaching 2400 K, yielding nickel nanoparticles measuring 75 nm in diameter, each adorned with a 4–5 nm carbon coating. Remarkably, these catalysts exhibited a striking 150‐fold improvement in electro‐oxidation performance within a H_2_O_2_ fuel cell, in stark contrast to counterparts that lacked the Joule heating treatment. Hard carbons, such as the ones derived from biomass, lack structural uniformity and conventional methods such as pyrolysis often produce a broad range of pore sizes in such materials. Using the joule heating method, such amorphous carbons can be modified to display a uniformity in structure and porosity which suits several potential applications. However, the utilization of the Joule heating method for producing porous carbon‐based materials for CO_2_ capture has scarcely been documented.

## 4 Material Characterization

In terms of materials science, characterization refers to the comprehensive and all‐encompassing process of probing and measuring a material's structure and characteristics. Without it, no scientific understanding of engineering materials could be established. It is an essential procedure in the field of materials research. It is required to analyze the physical and chemical properties of porous carbons using several analyses due to the existence of various types of porosity and functional groups on its surface. For example, analyses such as thermogravimetry‐differential thermal analysis are suitable not only for understanding the behavior of the final material but also for the primary precursors. Thermogravimetry‐differential thermal analysis (DTA/TG), transmission electron microscopy (TEM/HRTEM), Raman, powder X‐ray diffraction (XRD), field emission scanning electron microscopy (FESEM), CHNSO, and N_2_ adsorption‐desorption analysis were used to describe the morphology and framework, as well as the chemical composition of the resulting porous carbons materials.^[^ ^134^ ^]^

### 4.1 DTA/TG

The TG and DTA are performed using a thermogravimetric analyzer in N_2_/O_2_/Ar atmospheres within a temperature range of 25 to 1200 °C. TG and DTA are used to explore the pyrolysis behavior of biomass and the generated gaseous products as well as the effect of temperature on N/P doping on the porous carbon materials and their stability. The weight loss of the biomass at different temperatures can be obtained from TG analysis whereas DTA can be used for understanding the reaction whether it is endothermic or exothermic, as well as energy sorption throughout the thermal conversion.^[^ ^135^ ^]^ The weight loss that occurs below 200 °C is mostly related to the loss of water content from the biomass. The majority of the weight loss that occurs between 250 and 700 °C is due to the pyrolysis of hemicellulose and cellulose pyrolysis as well as the elimination of gaseous volatile such as CO and CO_2_ from the glucopyranose rings. Among the biomass components, the decomposition of lignin is very difficult and it starts to decompose across the entire temperature range of 25 to 1000 °C.^[^ ^136^ ^]^ Unlike polysaccharide polymers, lignin is hydrophobic with the ether and ß‐1,4‐glycosidic linkages in the molecular structure and resists water penetration. To create a reactive porous carbon, these molecular connections and linkages must be disrupted thermally or chemically.^[^ ^137^ ^]^ As all organic polymers degrade at high temperatures, ash together with the inorganic components is formed at the end, providing information about the details of the inorganic residues in the biomass.^[^ ^138^ ^]^ The loss of water is shown by the endothermic peak (downward slope in the chart), while the degradation of cellulose is indicated by the normally endothermic peaks (ascending slope) at high temperatures.^[^ ^139^ ^]^

**Figure 15:**
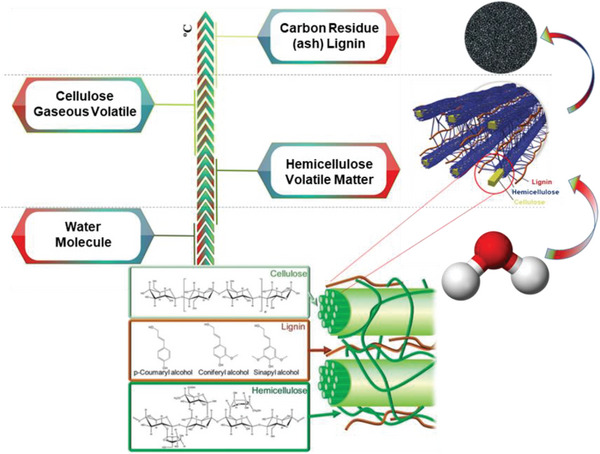
Overview of various components decomposition during pyrolysis: TG analysis has been performed for lignocellulosic biomass‐derived porous carbon, Reproduced with permission^[^ ^140^ ^]^ Copyright 2012 Royal Society of Chemistry.

**Figure 16:**
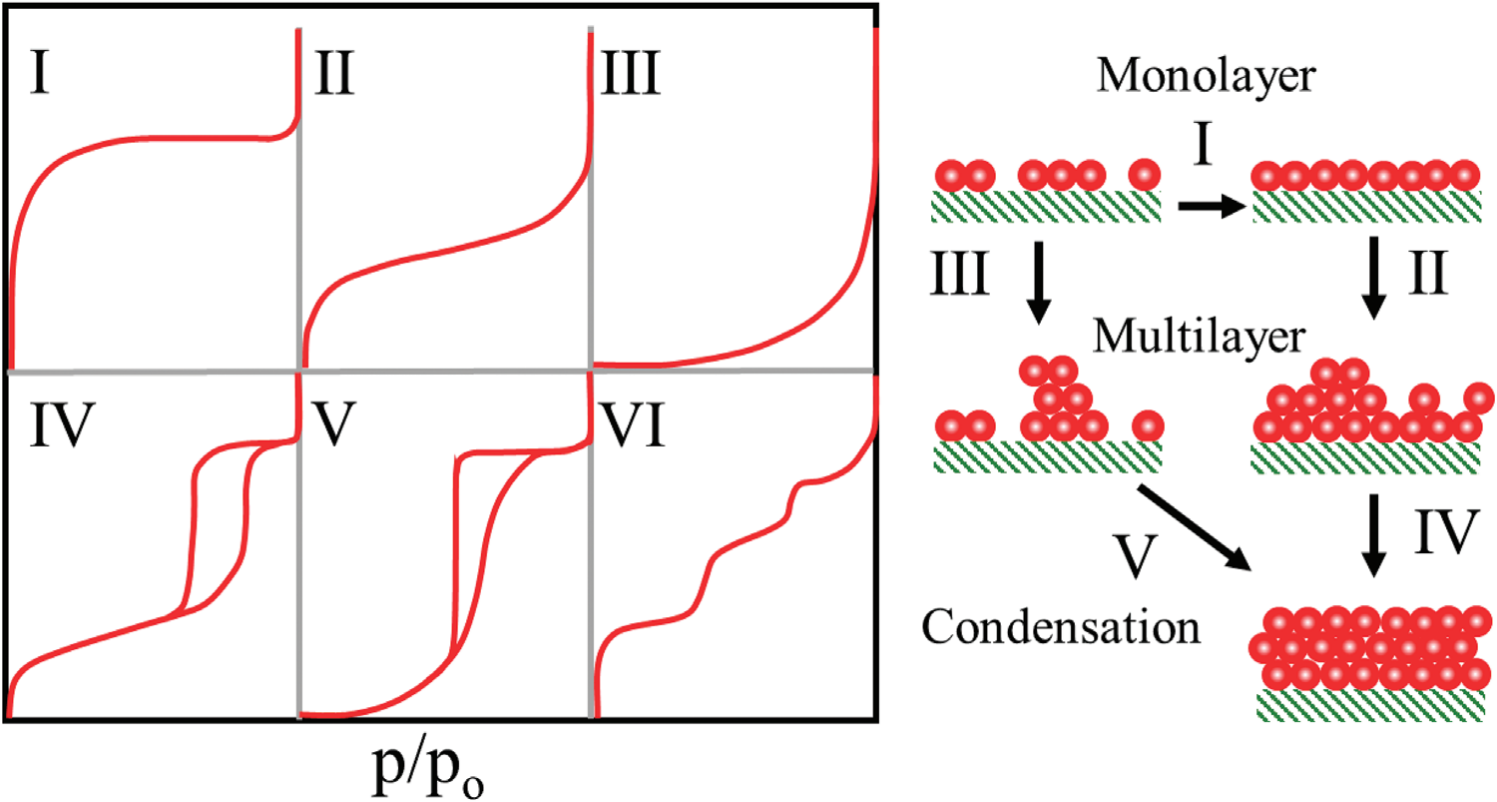
Types of adsorption isotherms based on the International Union of Pure and Applied Chemistry (IUPAC) classification, Reproduced under the term of ther Creative Commons Attribution 4.0 International License.^[^ ^161^ ^]^ Copyright 2018, The Authors, published by Scientific Research Publishing Inc.

From the TG analysis, the existence of oxygenated surface groups, which are responsible for hydrogen bonding involving water and oxygen molecules, can be determined as it causes mass losses in the first steps up to 200 °C for porous carbons as well as biomass in the TG tests. The surface groups generated during the activation phase, as well as the carbon skeleton degradation, may be allocated to the decomposition product of the second and third mass loss processes. In the second stage, less thermally stable groups such as carboxylic acids and lactones decompose with the evolution of CO_2_, whereas other surface groups such as pyrone, ethers, and phenol structures are expected to decompose at higher temperatures (third step).^[^ ^141^ ^]^ In some natural precursors, hemicellulose, cellulose, and lignin are absent. For example, animal bone is principally composed of collagen and hydroxyapatite (HA, Ca_x_ (PO_4_, CO_3_)_y_ (OH)) and is considered as one kind of natural organic/inorganic composite. For this sample, there is a substantial weight loss between 200 and 450 °C corresponding to the decomposition of collagen whereas the weight loss above 500 °C is related to HA (Figure 16).^[^ ^142^ ^]^ Some of the examples of the decomposition of the biomass as the analysis temperature increases are shown in **Figure** **15**.

### 4.2 Fourier Transform Infra‐Red (FT‐IR) spectroscopy

FT‐IR is an important tool to characterise the surface functional groups in porous carbons. These functional groups can play a significant role in interactions with the CO_2_ molecule, especially at low pressure. For example, the surface functional groups such as carbonyl (‐C═O), hydroxyl (‐OH), carboxyl (‐COOH), and amine (‐NH_2_) in porous carbons are easily detectable using FT‐IR and such groups can enhance their CO_2_ adsorption. Ghaemi et al. employed simulation studies to illustrate that the inclusion of carboxylic groups on the surface of porous carbons can generate Lewis basic sites. These sites play a pivotal role in augmenting electrostatic interactions with CO_2_ molecules.^[^ ^143^ ^]^ Through their FTIR analysis of the porous carbon, diverse functional groups were detected on the surface, including those corresponding to OH, C‐H, C═C, ‐C═O, and C‐O moieties.

FT‐IR spectroscopy can also be employed to track alterations in functional groups following chemical modifications of porous carbons. For instance, pristine activated carbon subjected to modification with iron chloride (FeCl_3_) exhibits the emergence of several functional groups that were initially absent.^[^ ^144^ ^]^ The FT‐IR spectrum reveals prominent peaks attributed to OH, C═C, C═O, C‐O, C‐H, and FeOOH groups introduced onto the activated carbon surface through the utilization of FeCl_3_. More sophisticated methodologies like in‐situ FT‐IR can be employed to probe the intricacies of the CO_2_ adsorption mechanism on a diverse range of materials.^[^ ^145^ ^]^ This can also offer valuable insights into the CO_2_ adsorption sites within porous carbons. In essence, FT‐IR serves as a crucial tool to unveil the surface chemistry of porous carbons.

### 4.3 XRD/RAMAN

The non‐destructive techniques of XRD and Raman spectroscopy are employed to explore the phases and crystal structure information of porous carbon.^[^ ^146^ ^]^ The XRD pattern provides important information such as the position of diffraction peaks, unit cell characteristics, and interlayer‐d spacing.^[^ ^147^ ^]^ Low angle XRD patterns are recorded in the 0.5–10 ° range, whereas wide‐angle patterns are acquired in the 10–80 ° range.^[^ ^148^ ^]^ Minor graphitic/turbostratic carbon domains and amorphous carbons can cause two characteristic peaks at 2θ = 22–24 ° and 2θ = 42–44 °, which are ascribed to the planes of (002) and (100), respectively (JCPDS X‐ray Powder Diffraction Database No. 75–1621).^[^ ^149^ ^]^ These two peaks do not necessarily coincide, and porous carbon can only exhibit one of them. The broadness of the peak and reduction in the intensity of the peak in the 22–24 ° range imply poor long‐range crystalline order, while the narrow peak represents enhanced crystalline order of the graphitic nature. Low degrees of graphitization encourage the formation of large amounts of porosity.^[^ ^150^ ^]^ The (100) diffraction peak stays constant when the carbonization temperature is raised, however, the amplitude of the (002) peak may drop which indicates a reduction in crystallite size and an increase in the disorder of the microstructures.^[^ ^58^ ^]^ Increasing the pyrolysis temperature affects the organization of graphene sheets in the porous carbons, causing a peak shift in for (002) plane to smaller angular positions, with a simultaneous intensity enhancement of this peak. This change suggests a decrease in the crystallite stacking height and an increase in the distance (d_002_) between the aromatic layers (Lc). The graphene structure becomes more disorderly when these values are reduced.^[^ ^151^ ^]^ It's worth noting that raising the temperature doesn't always make the structure more crystalline (1000‐1200 °C). Sometimes, the structure collapses at high temperature as pore necks are broken with the development of slit pores, and the formation of a turbostratic carbon skeleton. Bragg's equation and Scherrer's formula are used to compute the interlayer spacing of the aromatic planes d (002) and the crystallite stacking height (Lc). The Scherrer equation for the line broadening of the peak can be used to determine the crystallite size (d) of porous carbons:

(12) $$d=\frac{k\lambda }{\beta \cos\theta }$$

where the X‐ray wavelength is λ; the FWHM width of the diffraction peak is β; the diffraction angle is θ; and the k is constant. Bragg's Law is the fundamental connection between incoming X‐ray wavelength, incidence angle, and distance between crystal lattice planes of atoms represented as:^[^ ^152^ ^]^

(13) nλ=2dsinθ

where θ is the incidence angle, d is the interlayer spacing, and λ is the X‐ray wavelength. The formula N = Lc/d (002) was used to calculate the average number of graphene planes in the crystallites. The calculated interlayer spacing d (002) for a graphitic carbon is 0.33‐0.35 nm; hence, higher d (002) values are taken as a sign of a weaker degree of organization.^[^ ^153^ ^]^

The Raman spectra of porous carbons generally reveal two distinct bands, D‐band (1300‐1400 cm^−1^) and G‐band (1500‐1600 cm^−1^), which are associated with disordered carbon structures and crystalline graphitic carbon, respectively. The G band, which derives from the E2g stretching mode of sp^2^ carbons in the graphitic layered skeleton, displays the processed materials' graphitic character. The A_1g_ symmetry of carbons enriched with non‐planar structural distortions and crystal defects related to the D band. Furthermore, the symmetric 2D band (placed at 2650–2750 cm^−1^) has already been recognized as a significant characteristic for estimating graphene layer numbers based on its shape/deconvoluted peaks and location.^[^ ^154^ ^]^ Since the intensity ratio of the G and D bands (I_G_/I_D_) can be utilized to indicate the graphitic degree of carbon, and the I_G_/I_D_ ratio of porous carbon is observed to be higher than that of heteroatom‐doped porous carbon, it is established that nitrogen induces defects and disorder into carbon materials.^[^ ^115^, ^155^ ^]^

The following equation is used to calculate the interdefect nanocrystallite size:

(14) $$L_{d}\left(\mathrm{nm}\right)=\left(2.4*10^{-10}\right)*\lambda^{4}{\left(\frac{I_{D}}{I_{G}}\right)}^{-1}$$

The distance between defects is represented by L_d_, which is inversely proportional to relative intensities (I_D_/I_G_). Furthermore, the defect density can be calculated using:

(15) $$n_{d}\left({\mathrm{cm}}^{-2}\right)=\frac{\left(1.8*10^{22}\right)}{\lambda^{4}}*\left(\frac{I_{D}}{I_{G}}\right)$$

The excitation wavelength is denoted by λ in this equation (for green laser it is 514 nm). With higher I_D_/I_G_ ratios, the defect density (n_d_) rises while the distance between defects (L_d_) decreases.^[^ ^156^ ^]^

### 4.4 BET/BJH

To estimate the porosity characteristics of the carbons, a volumetric adsorption analyzer is used to assess the adsorption/desorption isotherms for N_2_ at 77 K.^[^ ^157^ ^]^ Adsorption isotherms are divided into six types, porous carbons being generally type I or IV isotherm models.^[^ ^158^ ^]^ These isotherms are shown in **Figure** **16**. The intensity of the interface between the sample surface and the sorbent, as well as the presence or absence of pores, are used to classify sorption isotherms. However, some samples do not quite match the type I or IV isotherm categorization. Adsorption isotherms may be combined in such samples. For porous specimens with a large specific surface area, N_2_ sorption may generate a mixture of type I and II or type I and IV isotherms.^[^ ^159^ ^]^ Some isotherms have no hysteresis loops and are horizontal throughout a large pressure range (The sharp upswing at a low relative pressure (p/p_0_ < 0.1)), suggesting that they are type I isotherms (IUPAC classification).^[^ ^160^ ^]^ As a result, the prepared porous carbons are microporous, with PSD curves below 2 nm. Exemplary adsorption‐desorption isotherms with a mix of type I and type IV can be seen in several samples generated at various temperatures or activated using different activating agents. The existence of multiple mesoporous and macroporous structures in the samples is indicated by the H1‐type hysteresis loop at a high relative pressure (p/p_0_ > 0.5). The V_meso_ of porous carbons rises as the activation duration is increased. As a result, their isotherms shift from type I to type IV.^[^ ^110^ ^]^

Since the porous structure of non‐porous carbons is nearly exclusively made up of ultra‐micropores, these materials have a very low N_2_ sorption capability. The diffusion rate of N_2_ molecules into ultra‐micropores is exceedingly sluggish at cryogenic temperatures. As a result, the S_BET_ values calculated from the N_2_ sorption isotherm for non‐porous carbon (biochars‐ash) dropped dramatically (0‐10 m^2^.g^−1^).^[^ ^162^ ^]^ The carbon porosity improves more as the pyrolysis temperature rises, as evidenced by the significantly increased N_2_ sorption. The etching process of carbon causes the S_BET_ and V_t_ of porous carbons to grow fast with the pyrolysis temperature.^[^ ^163^ ^]^ The etching action of the activating agent on the mesopore walls, which leads to the proliferation of more large‐size micropores, might be attributed to the formation of the micropore framework. However, raising the carbonization temperature (up to 1200 °C) may result in a significant decrease in porosity volume due to pore collapse caused by excessive activation and decreased resistance of porous walls. In addition, surface modification and doping of elements lead to a decrease in S_BET_, which can be attributed to the occupation of porosity by these elements or the formation of pore structural defects. When the biomass precursors decompose, volatiles are released, leaving char, which then undergoes an extensive gasification process when the temperature rises over 700 °C. The reaction of the carbon with CO_2_ gas produced by carbonization or used as an activator is known as the Boudouard reaction (CO_2_ + C ⇌ CO, endothermic, 175.52 KJ.mol^−1^). The constant of equilibrium reduces dramatically as the activation temperature rises. This means that this reaction is substantially faster in favor of CO formation and solid carbon consumption. The activation time is proportional to the time it takes for the carbon matrix and CO_2_ to react. Large amounts of carbon might react with CO_2_ to devour the solid material, resulting in more pores, as the activation period increases. Nevertheless, at very high temperatures (1000‐1200 °C), the equilibrium constant is very low, which can justify the reduction of the S_BET_.^[^ ^164^ ^]^

#### 4.4.1 Isotherms Modeling

In general, the adsorption phenomenon represents the balance between the amount of adsorbate and the adsorbate residue at the interface of the two gas‐solid phases, which are described at a constant temperature and pH.^[^ ^165^ ^]^ In other words, adsorption is the thermodynamic equilibrium at the interface of two phases.^[^ ^166^ ^]^ By obtaining the physicochemical parameters of the thermodynamic equilibrium of adsorption, valuable information can be obtained, including the mechanism governing the adsorption process and the adsorbent surface properties, which play an essential role in the design, modeling, and implementation of adsorption processes.^[^ ^167^ ^]^

Numerous experimental and modeling studies have been performed to investigate the adsorption mechanism of CO_2_ on adsorbents, which in general can be considered as four important steps: i) gas‐phase contact with the adsorbent surface, ii) penetration and diffusion in adsorbent pores, iii) interaction of CO_2_ with active adsorbent sites, and iv) the creation of a product on the adsorbent surface and its existing pores.^[^ ^168^ ^]^ In order to evaluate the adsorption processes and describe the adsorbent behavior, experimental and mathematical isotherm models have been developed in recent years, and matching these equations with experimental data can provide helpful information. The adsorption isotherms mentioned with the definition of their parameters are summarized in **Table** **2**. For each isotherm model, C_e_ and q_e_ are equilibrium adsorption capacity and equilibrium concentration, respectively. Many studies have been performed on the use of activated carbon derived from natural adsorbents to capture CO_2_. Yang et al. prepared porous activated carbon by hydrothermal route from tree leaves to study the adsorption isotherms of CO_2_. Langmuir and Freundlich's isotherms were used for all prepared porous carbons, and the correlation coefficient (R^2^) showed that Langmuir was the best isotherm to describe the adsorption process.^[^ ^169^ ^]^ In another research, He and coworkers studied the ability of N‐doped activated carbon produced from rice husk for CO_2_ capture. This study used three commonly used isotherm models, namely Langmuir, Freundlich, and Temkin, to fit the CO_2_ adsorption data. Experimental evidence showed that the adsorption behavior of as‐prepared activated carbon could be well described by the Freundlich model.^[^ ^170^ ^]^ Singh and Kumar modeled CO_2_ adsorption on the commercially activated carbon prepared from lignite granular content. According to the results, the DA isotherm model agrees better with the experimental results than the Longmuir isotherm model.^[^ ^36^ ^]^ In another research, Kim et al. activated ground‐based microporous carbons taken from spent coffee using K_2_CO_3_ and was utilized to adsorb CO_2_. The CO_2_ adsorption results at three different temperatures of 0, 25, and 50 °C agreed well with the Langmuir adsorption isotherm model.^[^ ^171^ ^]^ Parshetti et al. also prepared low‐cost carbonaceous adsorbents from a lignocellulosic feedstock. The adsorption data was fitted to Langmuir, Freundlich, and Temkin isotherm models. Adsorption isotherm modeling indicated that the Freundlich equilibrium model best matched the experimental data.^[^ ^172^ ^]^ Shao et al. used activated carbons derived from poplar wood as adsorbents to capture CO_2_. They found out that the Langmuir and Freundlich models had *R* ^2^ > 0.99, among which the Freundlich model indicated better agreement than Langmuir.^[^ ^173^ ^]^

**Table 2:** Adsorption isotherm models.

Isotherm model	Nonlinear form	Linear form	Model parameters	Reference
BET	$q_{e}=\frac{q_{s}C_{BET}C_{e}}{\left(C_{s}-C_{e}\right)\left[1+\left(C_{BET}-1\right)\left(\frac{C_{e}}{C_{s}}\right)\right]}$	$\frac{C_{e}}{q_{e}\left(C_{s}-C_{e}\right)}=\frac{1}{q_{s}C_{BET}}+\frac{\left(C_{BET}-1\right)}{q_{s}C_{BET}}\frac{C_{e}}{C_{s}}$	q_s_: Maximum adsorption capacity (mg g^−1^)	[174]
			C_s_: Monolayer saturation concentration (mg/L)	
			C_BET_: BET adsorption constant (L mg^−1^)	
DA	$q_{e}=q_{m,DA}\left[-{\left(\frac{\varepsilon_{DA}}{E_{DA}}\right)}^{n_{DA}}\right]$ $\varepsilon_{DA}=RT\ln\left(\frac{C_{s,DA}}{C_{e}}\right)$	$lnq_{e}=lnq_{m,DA}-n_{DA}ln\frac{\varepsilon_{DA}}{E_{DA}}$	q_m,DA_: Maximum adsorption capacity (mg g^−1^)	[175]
			n_DA_: DA isotherm heterogeneity parameter	
			C_s,DA_: Maximum solubility of the adsorbate (mg/L)	
			E_DA_: Characteristic energy of the system (kJ mol^−1^)	
DR	*q_e_* = (*q*_*m*,*DR*_)exp ( − *k_ad_* ε^2^) $\varepsilon =RT(1+\frac{1}{C_{e}})$	ln (*q_e_*) = ln (*q_s_*) − *k_ad_* ε^2^	q_m,DR_: Maximum adsorption capacity (mg g^−1^)	[176]
			k_ad_: DR isotherm constant (mol^2^/kJ^2^)	
Freundlich	$q_{e}=K_{F}C_{e}^{\frac{1}{n}}$	$\log q_{e}=\log K_{F}+\frac{1}{n}logC_{e}$	K_F_: Fitting constant ((mg g^−1^)(L mg^−1^)^1/n^)	[177]
			n: Freundlich isotherm constant	
Halsey	$q_{e}={\left(\frac{K_{H}}{C_{e}}\right)}^{\frac{1}{n_{H}}}$	$\ln q_{e}=\left(\frac{1}{n_{H}}\right)\ln K_{H}-\left(\frac{1}{n_{H}}\right)\ln C_{e}$	K_H_: Halsey isotherm constant	[178]
			n_H_: Halsey isotherm constant	
Henry	–	*q_e_* = *K_HN_C_e_*	K_HN_: Henry adsorption constant (L g^−1^)	[179]
Jovanovich	$q_{e}=q_{max}\left(1-e^{K_{J}C_{e}}\right)$	ln *q_e_* = *lnq_max_* − *K_J_C_e_*	q_max_: Maximum adsorption capacity (mg g^−1^)	[180]
			K_J_: Jovanovich constant (L g^−1^)	
Langmuir	$q_{e}=\frac{Q_{0}bC_{e}}{1+bC_{e}}$	$\frac{C_{e}}{q_{e}}=\frac{1}{bQ_{0}}+\frac{C_{e}}{Q_{0}}$	Q_0_: maximum adsorption capacity (mg g^−1^)	[181]
			b: Langmuir isotherm equilibrium constant (L mg^−1^)	
MET	$q_{e}=q_{s}{\left(\frac{k}{\ln(\frac{C_{s}}{C_{e}})}\right)}^{\frac{1}{3}}$	–	q_s_: Theoretical saturation capacity (mg g^−1^)	[182]
			k: MET isotherm constant	
			C_s_: Monolayer saturation concentration	
Radke‐Prausnitz	$q_{e}=\frac{a_{RP}r_{R}C_{e}^{\beta_{R}}}{a_{RP}+r_{R}C_{e}^{\beta_{R}-1}}$	–	a_RP_: Maximum adsorption capacity (mg g^−1^)	[183]
			r_R_: Radke‐Prausnitz isotherm equilibrium constant	
			β_R_: Radke‐Prausnitz isotherm exponent	
Sips	$q_{e}=\frac{q_{m,s}K_{s}C_{e}^{\beta_{S}}}{1+K_{s}C_{e}^{\beta_{S}}}$	$\beta_{S}\ln\left(C_{e}\right)=-\ln\left(\frac{K_{S}}{q_{e}}\right)+\ln\left(a_{s}\right)$	Ks: Sips isotherm constant (L g^−1^)	[184]
			βs: Sips isotherm exponent	
			q_m,s_: Maximum adsorption capacity (mg g^−1^)	
Temkin	$q_{e}=\frac{RT}{b_{T}}\ln A_{T}C_{e}$	$q_{e}=\frac{RT}{b_{T}}\ln A_{T}+\left(\frac{RT}{b_{T}}\right)\ln C_{e}$	b_T_: Temkin isotherm constant (J mol^−1^)	[185]
			A_T_: Temkin isotherm constant (L g^−1^)	
Toth	$q_{e}=\frac{q_{m,TO}C_{e}}{{\left(a_{T}+C_{e}\right)}^{1/t}}$	$\ln\left(\frac{q_{e}}{q_{m,TO}}\right)=\ln\left(C_{e}\right)-\frac{1}{t}\ln\left(a_{T}+C_{e}\right)$	q_m,TO_: Maximum adsorption capacity (mg g^−1^)	[186]
			a_T_: Toth isotherm constant (L g^−1^)	
			t: Toth dimensionless parameter	

### 4.5 FESEM/HRTEM

**Figure 17:**
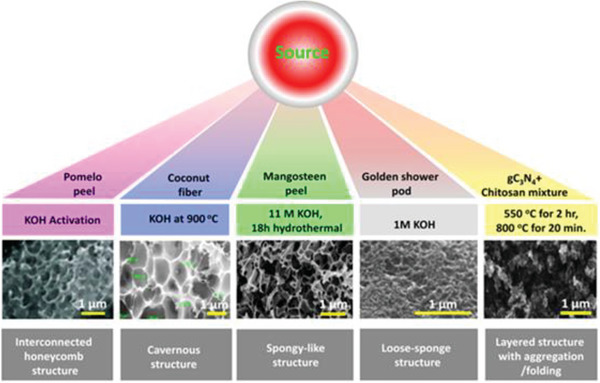
Different types of morphologies for porous carbons obtained using FESEM. From left to right) Reproduced with permission.^[^ ^187a^ ^]^ Copyright 2018, Elsevier;^[^ ^187b^ ^]^ Copyright 2021, American Chemical Society;^[^ ^187c^ ^]^ Copyright 2018 Elsevier; reproduced under the term of the Creative Commons CC BY license^[^ ^187d^ ^]^ Copyright 2020, The Authors, published by MDPI; reproduced with permission.^[^ ^187e^ ^]^ Copyright 2019, ACS.

FESEM and HRTEM micrographs are used to assess the morphology and microstructure of the porous carbons. Honeycomb, cauliflower, cave‐like, sphericalm and other morphologies are common among synthesized structures as shown in **Figure** **17**.^[^ ^187^ ^]^

**Figure 18:**
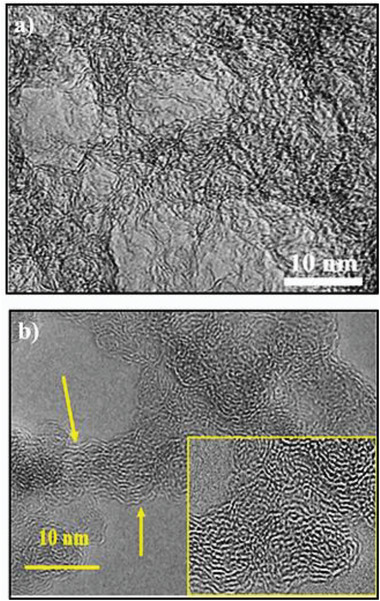
a) HRTEM micrographs of graphitized structure, Reproduced with permission^[^ ^190^ ^]^ Copyright 2018 Elsevier, and b) wormhole‐like pore structure Reproduced with permission^[^ ^191^ ^]^ Copyright 2020 Elsevier.

The existence of compact aggregates of graphitic and microporous carbon resulting from FESEM indicates that the surface of porous carbon usually is heterogeneous. Inside the amorphous carbon matrix, these morphologies generally consist of isolated aggregates and distributed aggregates. Furthermore, the high cellulose fiber contents on the surface of produced porous carbons can generally be detected from FESEM images. In some cases, the precursor or non‐porous carbon typically has a solid bulky shape with a smooth surface and no pores. The solid bulky particles transform into a spongy‐like 3D structure with numerous irregular cottony pores during carbonization.^[^ ^142^, ^188^ ^]^ These unique features of the porous carbons can be obtained by the HRTEM.^[^ ^189^ ^]^ Several tiny graphite stripes of the porous carbons can be seen in the HRTEM images, indicating a higher degree of graphitization in the waste cellulose‐derived porous carbon prepared using zincoxen (**Figure** **18**a).^[^ ^190^ ^]^ Conversely, some synthesized porous carbons are amorphous, with many wormhole‐like pore structures^[^ ^191^ ^]^ with the graphite sheet spacing of 0.33‐0.35 nm (Figure 18b), as seen by the HRTEM images.^[^ ^78^, ^188^ ^]^

## 5 CO_2_ Capture

**Figure 19:**
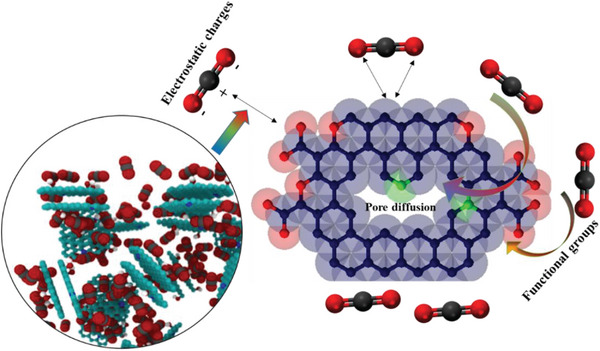
The general mechanism of physical adsorption of CO_2_ through diffusion (induction) in interconnected porosity or through functional groups.

Solid adsorbents, as opposed to liquid sorbents (Chemical absorption), are frequently used for CO_2_ capture. Owing to their low cost, eco‐friendliness, ease of manufacture, ideal thermal‐chemical stability, accessibility, hydrophobicity of surface, sustainability, and customizable pore shape, porous carbon‐based materials are of special interest.^[^ ^192^ ^]^ Depending on the type of sorbent‐sorbate interactions, the adsorption process is generally divided into physical adsorption (Physisorption) and chemical adsorption (Chemisorption).^[^ ^193^ ^]^ Physisorption (as depicted in **Figure** **19**) is also called van der Waals adsorption, because there is no chemical bond between the adsorbent and the adsorbate in this type of sorption, and only weak van der Waals bonds (Ion‐quadrupole interaction) are formed between the gas molecules and the porous carbon surface.^[^ ^194^ ^]^ The forces that hold gas molecules on the porous carbon surface are easily eliminated by applying heat or reducing pressure. Therefore, each of these parameters can be used in the regeneration and increment of adsorption cycles of adsorbents.^[^ ^195^ ^]^

Conversely, chemisorption involves the creation of strong covalent bonds between the adsorbent and the adsorbate molecules, and the transfer of electrons occurs between them (mainly using C‐rich material supported by amine groups). Thus, adsorption enthalpy is much higher for chemisorption than that of physical adsorption.^[^ ^196^ ^]^ Due to the high bond energy between the adsorbent and the adsorbate in this process, it is expected that the chemisorption is not easily reversible, unlike the physisorption. Therefore, more energy is needed in the regeneration and renewability of the porous carbons.^[^ ^197^ ^]^

Both physisorption and chemisorption mechanisms on the heterogeneous surface can be distinguished by thermodynamic parameters, including the Gibbs free energy changes (Δ*G*°), enthalpy changes (Δ*H*°), and changes in entropy (Δ*S*°). Adsorption generally occurs physically on the surface of porous carbon, which makes it easier to regenerate the adsorbent with minimal energy requirements. Parameters such as standard enthalpy (the adsorption of energy in the form of heat) and isosteric heat (*Q*_st_) for physisorption are usually < 20 kJ mol^−1^, while chemisorption is in the range of 40–200 kJ mol^−1.[^ ^198^ ^]^ Pore structure and surface chemical characteristics are two important parameters that influence the CO_2_ adsorption behavior of a porous material. The first one affects the contact forces between gas molecules and the carbon surface, while the second factor controls the active sites available for admitting CO_2_ molecules.^[^ ^56^, ^199^ ^]^ According to the Dubinin equation (D‐R) on porous carbons, pore‐filling is the major CO_2_ sorption mechanism, which means gas molecules are trapped in the narrow micropores by surface physisorption.^[^ ^200^ ^]^

(16) $$V=V_{0}\exp\left[-{\left(\frac{A}{\beta E_{0}}\right)}^{2}\right]$$

In the D‐R equation, V is the filled volume, V_0_ is the micropore volume, A = RTln (p/p_0_), E_0_ is the characteristic energy coefficient, and ß is the affinity coefficient with a value of 0.35.^[^ ^201^ ^]^ The quadrupole nature of the CO_2_ molecules has been proposed as a beneficial property for producing a surface contact (pole/ion and pole/pole interactions) with porous carbons via the dispersion and induction processes. When molecules reach the sorbent surface, their free energy decreases, causing the gas molecules to attract to the sorbent surface's electronic surroundings. The interactions between the molecules and the solid surface, as well as the resulting reduction in entropy, result in an increase in the amount of CO_2_ molecules at the sorbent surface.^[^ ^195a^ ^]^ To sum up, it can be concluded that gas molecules initially tend to diffuse into the pores, interact between the quadruple of molecules, and the ionic‐polar sites of the sorbent surface and then after reaching equilibrium (adsorbent saturation), multilayer adsorption (surface diffusion) begins.^[^ ^202^ ^]^

The isosteric heat of adsorption (Q_st_) is always considered when designing and operating a gas sorption process to determine the temperature change in the sorption process. In addition, the Q_st_ is a measure of the capacity of the sorbent for regeneration. The Q_st_ may also explore the energy heterogeneity of a sorbent's surface*.^[^* ^203^ ^]^ The Clausius–Clapeyron equation could be used to calculate the single component isosteric heat of sorption as a function of surface loading:

(17) $$\frac{-Q_{st}}{R}={\left(\frac{\partial \ln p}{\partial T^{-1}}\right)}_{n}$$

where Q_st_ (kJ.mol^−1^) represents the isosteric heat of sorption, T (K) represents the temperature, P (kPa) represents the pressure, R represents the gas constant (8.314 J.mol^−1^.K^−1^), and q (mmol g^−1^) represents the adsorbed quantity.^[^ ^198a^ ^]^

Based on the assumption that the isosteric heat of sorption is independent of the T, integration of aforementioned equation gives:

(18) $$\ln P=\frac{Q_{st}}{RT}+\mathrm{constant}$$

Using equilibrium isotherm data, the isosteric heats of sorption can be estimated using the slopes of linear plots of ln P versus 1/T.^[^ ^204^ ^]^

### 5.1 CO_2_ adsorption technologies

Adsorption process technologies are made up of two primary processes: adsorption of gas and regeneration of adsorbent. The adsorption process involves passing a gas mixture over the adsorbent bed under appropriate operating circumstances for the adsorption of the gas molecules, followed by the regeneration step, which involves separating the trapped gas molecules from the adsorbent and recycling the adsorbent. As a result, the adsorption process is intimately linked to the desorption process used for the adsorbent regeneration process. Pressure Swing Adsorption (PSA),^[^ ^205^ ^]^ Temperature Swing Adsorption (TSA),^[^ ^206^ ^]^ Vacuum Swing Adsorption (VSA),^[^ ^207^ ^]^ and Electrical Swing Adsorption (ESA)^[^ ^208^ ^]^ technologies are some of the cyclical adsorption techniques utilized in gas adsorption. PSA and TSA are commonly utilized in CO_2_ separation. PSA attracts CO_2_ molecules to the surface of the adsorbent by growing pressure, then releases the CO_2_ molecules in the evacuation phase by lowering pressure. VSA is a new technique that captures CO_2_ at pressures close to those found in the atmosphere.^[^ ^35b^ ^]^ TSA, on the other hand, heats the adsorbent with steam to absorb CO_2_ and minimize heat for the desorption process. Because adsorption from the gas phase is an exothermic process, increasing the temperature causes the equilibrium to shift to lower applied loads, resulting in desorption. PSA and VSA have several benefits against TSA, including extremely quick adsorption, high efficiency, the ability to employ PSA for bulk adsorption, eco‐friendly, regeneration of adsorbent, and minimal operational costs.^[^ ^209^ ^]^ It is vital to note that the gas adsorption process could occur when adsorbent elements have varied adsorption capacities. Moreover, a favorable adsorbent might as well have desirable characteristics such as high selectivity for CO_2_, highest adsorption capability, and regenerability, as well as high potential adsorption kinetics. Rajagopalan et al.^[^ ^210^ ^]^ addressed the limitations associated with adsorbent selectivity, specifically focusing on its connection solely to adsorbent characteristics rather than its effectiveness at the process level. Their research aimed to investigate post‐combustion CO_2_ adsorption and its implications. The selectivity of potential adsorbents for CO_2_/N_2_ separating using a PSA method has been studied. Merely having a high selectivity level is unlikely to be sufficient when selecting the optimal adsorbent for CO_2_ capture. While the selectivity of the adsorbent materials may be excellent, if the adsorbent possesses a relatively low capacity, the overall process is likely to become considerably expensive. This research shows that in complicated, dynamic adsorption processes, (PSA and VSA processes for CO_2_ capture), the practical efficiency of a given solid adsorbent must be evaluated in the process plant using simulation and optimization. Since the primary objective of each gas separation (CO_2_ capture unit) is to fulfill the design objectives at the lowest cost, the next step in defining realistic assessment criteria for solid adsorbent screening is to link existing adsorption process modeling platforms to process‐economic analysis. As a result, the first objective should be to develop adsorbents having high adsorptive capacity and selectivity, followed by optimizing the adsorption process using the mentioned adsorbent, and finally the interaction among technical and economic analysis and adsorption modeling.^[^ ^211^ ^]^ A comparison of the three CO_2_ capture techniques including PSA, VSA, and TSA is shown below in **Table** **3**.^[^ ^212^ ^]^

**Table 3:** Summary of various parameters of PSA, VSA, and TSA.^[^ ^212^ ^]^

Technique	Principle	Adsorption conditions	Desorption conditions	Advantages	Disadvantages
PSA	Relies on pressure variation to regenerate the sorbent	High pressure is used to adsorb CO_2_	Lower pressure close to atmospheric pressure is used to desorb CO_2_	Most used technology at the industrial scale for gas stream separation due to its ease of use and viability	May not be economical to compress CO_2_ along with N_2_ in real flue gas conditions. Sorbent selectivity is low at high pressures.
VSA	Relies on pressure variation to regenerate the sorbent	The CO_2_ adsorption occurs at atmospheric pressure	The CO_2_ desorption is performed under a vacuum		Low CO_2_ purity and low CO_2_ recovery
TSA	Relies on temperature variation to regenerate the sorbent	The CO_2_ adsorption is performed at low temperatures for maximising adsorption	The CO_2_ desorption requires heating in a range of 150–200 °C	Relatively pure CO_2_ can be obtained as compared to other techniques	The high heat required for sorbent regeneration makes the process expensive

### 5.2 Parameters Affecting CO_2_ Adsorption

**Figure 20:**
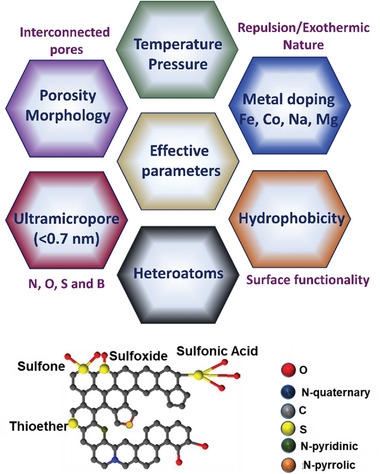
Overview of the factors affecting the sorption of CO_2_ on natural wastes‐derived porous carbons.

The adsorptive capability of porous carbon depends on several parameters. Some parameters directly and others indirectly affect the adsorption quantity. The meaning of indirect parameters is that some synthesis conditions such as a change in pyrolysis temperature, the flow rate of gases during synthesis, acid pickling, and retention time of the sample at the final pyrolysis temperature affect the high specific surface area which eventually determines the total adsorption capacity. However, some articles have reported that the high specific surface area and the total volume of porosity obtained from changing the synthesis conditions do not have much effect on gas adsorption, but the type of porosity resulting from the synthesis method can play an effective role in trapping gas molecules.^[^ ^1^, ^5^ ^]^ Two determinants influence CO_2_ adsorption: intrinsic factors and external parameters. The order of porosity, the volume of micropores and ultramicropores, the presence of heteroatoms (intrinsic and doped), hydrogen bonds, the type of nitrogen, the ratio of O/N, surface, and functional groups are all intrinsic variables. On the other hand, external factors such as moisture, temperature, and pressure of adsorption affect the final adsorption capacity of the adsorbents.^[^ ^50^, ^213^ ^]^ In the next section and in **Figure** **20**, all these parameters will be described in detail. In addition, the characteristics of the synthesized biomass‐derived porous carbons and their adsorption capacity are summarized in **Table** **4**.

**Table 4:** Summary of synthesis methods and CO_2_ adsorptive capability on optimal biomass‐derived porous carbons.

Biomass	Method	Reagent	Condition	V_micro_/S_BET_ [cm^3^ g^−1^/m^2^ g^−1^]	N/O/S [%]	Adsorption [mmol g^−1^] 0 °C/25 °C 1 bar	Selectivity (CO_2_/N_2_) (IAST) 25 °C	Isosteric Heat [kj mol^−1^]	Reference
Date sheets	Chemical activation	KOH	800 °C Ar flow‐2 h Mass ratio KOH/Sample = 4	0.834/2367	–	6.4/4.36	–	15.62‐17.6	[86b]
Slash pine wood	Chemical activation	KOH	853 K KOH/Sample = 4	–/906	–	4.93/–	–	24.9‐39.7	[214]
Shrimp shell‐derived chitosan	Chemical activation	KOH/Melamine	KOH/ Melamine/ Sample = 3/1/1 Carbonized at 600 ◦C‐ carbonized again (second step) at 900 ◦C	1.26/2095	∼4.1/13.4/–	10.5/6.41	42.3	20‐34	[87]
Corncob	Chemical activation	ammonia (NH_3_)	800 °C under N_2_ flow‐3h	–/1154	11/17/–	4.4/2.81	82	30.5‐55	[98]
Chitosan	Molten Salt Template	LiCl‐ZnCl_2_	molten salt/chitosan mass ratio (3/1)−1000 °C	0.56/2025	5.1/–/–	7.9‐5.6	–	24‐37	[107]
Rambutan peel	Hydrothermal	KOH	water‐soaking pre‐treatment/850 °C for 120 min KOH/Sample = 2	0.613/1487	–	–/5.4	–	–	[215]
Tree leaves (camellia)	Hydrothermal	KOH	240 °C‐5 h KOH/Sample = 3	0.7/1823.77	2.15/7.32/–	–/0.9	–	–	[169]
Corn stover	Hydrothermal	KOH	800 °C‐2 h KOH/Sample = 2	0.86/2442	0.24/–/–	7.14/–	15.5	–	[216]
Waste tobacco stem	Chemical activation	KOH	700 °C at 3 °C min^−1^ for 1 h under N_2_ KOH/Sample = 2	0.78/1922	–/8.07/–	7.98/4.84	–	–	[217]
Shrimp‐shells	Chemical activation	KOH	700 °C under N_2_ KOH/Sample = 2	0.66/1759.5	1.93/–/–	6.82/3.77	47	–	[218]
Gelatin and starch	Dry chemical activation	KOH	700 °C under N_2_ KOH/Sample = 4 Gelatin/starch 1:1	–/1636	3.22/22.57/–	7.49/3.84	51	27.6‐62.9	[219]
Lotus stalks	Chemical activation	KOH	melamine – 550 °C under N_2_ KOH/Sample = 2	–/1941	3.65/22.91/–	6.59/4.25	42	20‐28	[220]
D‐glucose	Hydrothermal	K_2_CO_3_	urea treatment at 350 °C in air 650 °C‐ K_2_CO_3_/Sample = 4	–/1824	7.74/–/–	6.23/3.92	44	28‐40	[221]
Algae	Direct carbonization	–	900 °C	–/450	–	–/6	–	–	[222]
Filter paper	Physical activation	CO_2_	850 °C	0.42/1114	–	5.12/3.29	19	25‐25.5	[223]
Corn starch	Chemical activation	Thiourea K_2_C_2_O_4_	600 °C for 1.5 h Activation/carbonization at 800 °C for 2 h under N_2_	0.93/1747	–	12/8.4	62	30‐39.3	[201]
Palm sheath	Chemical activation	KOH	650 °C under N_2_ KOH/Sample = 2	0.35/840	–	5.28/3.48	32.7	–	[224]
Enteromorpha	Chemical activation	KOH	800 °C KOH/Sample = 1	–/60.2	–	–/0.52	–	–	[225]
Pomegranate peels	Chemical activation	KOH	700 °C under N_2_ KOH/Sample = 1	0.42/585	–	6.03/4.11	15.1	–	[226]
Palm Kernel Shell	Physical activation	CO_2_	850 °C	–/367.8	3.3/29.8/0.05	–/2.13	–	27‐28	[227]
Pine Cone Shell	Chemical activation	KOH	650 °C KOH/Sample = 2	–/3135	1.03/9.26/–	7.63/4.73	17	26	[50c]
Crab shell	Chemical activation	KOH	650 °C KOH/Sample = 2	0.32/1196	6.3/14.4/–	6/4.37	–	–	[228]
Poplar catkin	Chemical activation‐ self‐doping	ZnCl_2_	ZnCl_2_/Sample = 4 800 °C	0.47/1455.1	1.57/6.61/–	4.05/6.22	89	19‐32	[229]

#### 5.2.1 Porosity

Carbon frameworks with hierarchical porosity, comprised of mesopores (2‐50 nm) in combination with macropores (>50 nm) or micropores (2 nm), are particularly well suited for CO_2_ uptake. The porosity of various sizes blends high specific surface areas with interconnected pores to enhance molecule diffusion (shorter diffusion path) throughout the entire surface.^[^ ^118b^ ^]^ The most significant benefit of interconnecting porosities is that the blind pores are not filled by the accumulation of gas molecules, which leads to porosity having a broader space to adsorb more molecules. Creating a wider space leads to an increase in the contact surface of molecules with active sites for adsorption. Furthermore, the adsorption kinetics are greatly influenced by the paths generated in the communication channels between the pores.^[^ ^230^ ^]^ Notably, only the interconnected paths in the hierarchical structure do not dramatically increase the gas adsorption rate, but the order of porosity plays a more influential role in CO_2_ capture.^[^ ^231^ ^]^ Mostly, a complimentary parameter, such as the order of porosity, along with the hierarchy of the structure, is required to fairly evaluate the CO_2_ uptake ability of materials.

Thanks to their unique pore features, namely, an ordered porosity predominantly made up of uniform mesopores, ordered mesoporous carbons (OMCs‐2 dimensional hexagonal symmetry) generated via hard or soft templating techniques are useful for CO_2_ uptake.^[^ ^7^ ^]^ One of the most important surface features in this type of porous carbon is the homogeneous surface, which significantly enhances gas adsorption. Furthermore, a certain number of ordered mesoporous channels can purvey an appropriate place for gas molecule diffusion. The van der Waals force of interaction between the carbon structure and gas molecules is generated by the micropore covering the mesoporous walls, boosting the amount of gas adsorption.^[^ ^20a^ ^]^ Also, compared to the microporous skeleton or porous carbon with non‐ordered mesopores, the well‐ordered mesopores in micro‐mesoporous carbon can enhance CO_2_ transportation characteristics.^[^ ^232^ ^]^ D‐glucosamine is being studied as a nonintoxicating and long‐lasting C/N biomass feedstock for the templated synthesis of N‐containing CMK‐8 ordered mesoporous carbons (NOMCs) aimed at efficient CO_2_ capture. The sorption of CO_2_ on OMC is 0.97 mmol g^−1^ at 30 °C under 0.9 bar, but this quantity rises with the presence of N groups on NOMC (1.47 mmol g^−1^ at 30 °C under 0.9 bar).^[^ ^233^ ^]^ In another research, CO_2_ uptake was reported by Enrique et al. using hierarchical ordered micro‐mesoporous carbons obtained from coal tar. The optimal samples showed CO_2_ capture capacities of 2.38 mmol g^−1^ and 2.18 mmol g^−1^ at 25 °C and 90 vol% CO_2_ in N_2_, via different synthesis conditions.^[^ ^234^ ^]^ Although this sort of porous carbon can be hierarchical, it has fewer microporous counterparts, making gas adsorption difficult. As a result, to generate micropores, the synthesis procedure must be sophisticated, and tuned with the appropriate reaction conditions. In addition, the use of a template requires exorbitant costs, so achieving the order parameter for use on an industrial scale is not economically viable.^[^ ^235^ ^]^

#### 5.2.2 Micropore Size

The activation variables, such as the reagents and the activation temperature, can largely determine the final pore structures. By etching the carbon structure of porous carbon with various chemicals, vast numbers of narrow micropores, tiny mesopores, and large macropores can be created. The findings demonstrate that the macroporous (50 ≤ d ≤ 100 nm diameter) structure allows for fast gas movement, while micropores (≤ 2 nm diameter) are the critical adsorption sites, implying that the linked macropore and microporous architectures assure high sorption performance of porous materials.^[^ ^3^, ^326^ ^]^ The prospective fields of adjoining walls overlapped, and the interaction energy was dramatically increased as the ultramicropore (≤ 0.7 nm diameter) structure evolved, resulting in an excellent CO_2_ sorption capacity. In porous sorbents, a more narrow microporous volume indicates the availability of stronger sorption sites. The microporous properties of materials regulate variables that affect how much CO_2_ can be adsorbed at low pressures. Gas molecules have weak kinetic energy at low pressures and, as a result, have a strong inclination to slither into small pores in the micropore domains. The inner and outer surfaces of the micropores get filled with CO_2_ molecules in a monolayer pattern.^[^ ^2^ ^]^ Similarly, tiny mesopores (2–4 nm) have a significant impact on CO_2_ sorption at high pressure (e.g., 30 bar).^[^ ^9^ ^]^ Nevertheless, due to diffusional restrictions, these micro‐and mesopores may slow down sorption and desorption kinetics, and the capillary forces in the micropores might also reduce adsorption rates.^[^ ^237^ ^]^

#### 5.2.3 Heteroatoms

Surface modification and functional group changes appeared to be the most effective technique to boost adsorbent capacity after ultramicropores. The majority of the research has concentrated on amine functionalization, chemical/physical activation, and heteroatom doping of porous carbon materials. N, O, S, B, and their mixtures, are the most common nonmetallic doping heteroatoms in porous carbon materials.^[^ ^20^, ^73^, ^238^ ^]^ For nitrogen doping, its role in gas uptake can be examined from three perspectives: as an inert gas in the carbonization process, intrinsic N source originating from biomass and non‐intrinsic N source from additives, and type of nitrogen.^[^ ^192a^ ^]^ N atom has the same atomic radius as C atom and shares 5 valence electrons. As a result, the physico‐chemical characteristics of carbon materials, including electronic features, conductivity, basicity, and oxidation, can be modified by manipulating the doping quantity and doping process.^[^ ^187e^ ^]^ The presence of nitrogen as an inert gas in the carbonization process is the first indirect beneficial role of nitrogen. The S_BET_ and V_t_ of porous carbons have been discovered to be affected by the flow rate of protective gas employed during the activation process. Dawei et al. evaluated various flow rates of nitrogen (100 mL min^−1^ to 1040 mL min^−1^) and discovered that a moderate flow rate (800 mL min^−1^) resulted in a 1.86 mmol g^−1^ (0.15 bar, 25 °C) sorption.^[^ ^10^ ^]^ Since N has a higher electronegativity than C, N‐doping can improve the surface polarity, thus increasing hydrophilicity and surface basicity of carbons, which improves CO_2_ sorption capacity.^[^ ^57^, ^239^ ^]^ Moreover, N‐doping can modify the surface chemical characteristics of carbon compounds by introducing superficial adsorption sites owing to altering the atomic charge density and asymmetric spin density.^[^ ^240^ ^]^ The strong dipolar C═O connections give the CO_2_ molecule a significant electric quadrupole moment. Doping introduces polar groups onto the carbon structure, which can cause local polarization/charge separation. As a result, N‐containing species greatly enhance carbon surface polarity, causing an electrostatic‐field gradient around the carbon surface. The strong interaction between the CO_2_ molecule quadrupole moment and the high electrostatic potential of N‐doped carbons improves CO_2_ adsorption energy in the electrostatic field, culminating in CO_2_ sorption reinforcement.^[^ ^241^ ^]^ Using quantum chemical calculations and FT‐IR measurements, Wei et al. demonstrated that adding N to a carbon surface improved hydrogen‐bonding interactions between the carbon surface and gas molecules, which accounted for improved CO_2_ sorption.^[^ ^242^ ^]^ In addition to the mentioned parameters, the type of nitrogen formed by the synthesis temperature plays an important role in the sorbent ability. However, the function of nitrogen in adsorption appears to be riddled with inconsistencies. According to some research studies, pyridonic nitrogen contributes much more to CO_2_ capture than pyridinic and quaternary (graphitic) nitrogen. Wang et al., on the other hand, discovered that quaternary N played a larger role in sorption.^[^ ^243^ ^]^ In another work, Sevilla et al. assessed the CO_2_ capture capability of nitrogen‐free and nitrogen‐doped porous carbon and discovered that nitrogen functional groups have no discernible effect on CO_2_ capture.^[^ ^244^ ^]^ As a result, it seems that the role of nitrogen is still in much dispute.

It is vital to evaluate oxygen‐derived functional groups to investigate the role of oxygen in CO_2_ adsorption. The high prevalence of carboxylic acid (C(═O)OH) and hydroxyl (‐OH) functional groups, comprising over 90% of all oxygen‐containing functional groups, suggests that the total number of oxygen‐containing groups could have an impact on CO_2_ adsorption.^[^ ^245^ ^]^ These oxygenated groups are believed to create the negative charge on the surface, which, when combined with a large surface area, might be very effective for the sorption of weakly acidic CO_2_ molecules by providing more binding sites.^[^ ^94^ ^]^ It has also been discovered that oxidizing carbon surfaces with a mixture of sp^2^ and sp^3^ oxygen atoms is more thermodynamically advantageous than oxidizing carbon surfaces with either sp^2^ or sp^3^ oxygen atoms alone, resulting in enhanced basicity. Surface basicity plays a crucial role in facilitating the adsorption of acidic molecules, such as CO_2_. Conversely, an acidic surface would be disadvantageous for this purpose.^[^ ^246^ ^]^ The materials thermally treated at high pyrolysis temperatures generates more oxygen‐containing functional groups.^[^ ^192b^ ^]^ However, the role of oxygen groups, similar to nitrogen doping, remains a topic of debate. Yin et al. suggest that surface oxygen‐containing groups do not affect CO_2_ sorption.^[^ ^247^ ^]^ Conversely, studies by Hao et al. and Wang et al. indicate that oxygen‐containing compounds enhance CO_2_ uptake while diminishing CH_4_ sorption in coal.^[^ ^248^ ^]^ Evidently, these works demonstrate that multiple factors simultaneously influence the ultimate sorption performance, emphasizing the need for further extensive research to obtain a conclusive answer.

Another widely employed heteroatom doping on the porous carbon surface is with sulfur. In comparison to other conventional dopants, such as boron and nitrogen, sulfur is peculiar since this element is considerably larger than carbon atoms and introduces more distinct types of defects, which promotes surface redox reactions and allows for greater CO_2_ sorption. The polar interaction of CO_2_ with sulfur oxide (168.5 eV) and the acidic interaction of CO_2_ with neutral sulfur (163.7 and 165.0 eV) can help enhancing CO_2_ sorption by sulfur‐containing functional groups in the porous carbon structure. The following are some of the proposed unique properties of sulfur functionalities on carbon surfaces: 1) increased induced polarizability and interactions with O, 2) increased local reactivity caused by the lone pair of electrons of the S atom, and 3) change in the electronic configuration of the system by shifting the Fermi level (E_F_: At absolute zero, an electron can occupy the greatest energy level possible) towards conduction band.^[^ ^249^ ^]^ Due to the lone pair electron donation of the S‐atom, S‐doping can also improve local reactivity. Intense acid‐base interactions between CO_2_ and basic C‐S functionalities, as well as strong pole‐pole interactions due to large quadrupole moment of CO_2_ and the polar S‐groups, indicate that the S‐doping of porous carbon might play a dominating role in CO_2_ sorption.^[^ ^250^ ^]^ In the S‐doped porous carbons, Seema et al. found a linear connection between CO_2_ capacity and oxidized‐S amount.^[^ ^251^ ^]^ The doping investigation is not limited to single elements but also their combination for increasing the adsorption rate. For example, Ghazanfar et al. created N and S‐dual doped corn‐starch‐based porous carbons that exhibited remarkable CO_2_ sorption (12.03 mmol g^−1^ at 0 °C and 1 bar).^[^ ^201^ ^]^ It is worth noting that creating heteroatom‐doped porous carbons typically necessitates using expensive, toxic, and corrosive raw materials, as well as complicated, difficult, and time‐consuming synthesis techniques. Due to the inconsistencies in researching, the involvement of heteroatoms in CO_2_ absorption, non‐experimental approaches such as modeling and machine learning must be used to corroborate the findings.^[^ ^252^ ^]^

#### 5.2.4 Hydrophobicity

Since CO_2_ must be adsorbed from a moist stream of flue gases in an industrial setting, porous materials with inherent properties, such as high hydrophobicity (or even superhydrophobicity) and humid stability, are required.^[^ ^2^, ^31^ ^]^ Materials such as zeolites are good for CO_2_ adsorption, however their surface is hydrophilic. Even though hydrophobicity does not play a direct role in gas adsorption, the hydrophobic surface (due to the contact angles with water between 90–180°) prevents H_2_O molecules from colliding with the porosity surface.^[^ ^253^ ^]^ To this end, the surface functional groups improve CO_2_ interactions while also increasing hydrophobicity. Functionalization with amine groups is frequently used to generate non‐hydrophilic sites on the pore surface, providing better control over gas adsorption in humid environments. However, in addition to the mentioned advantages, surface chemistry modification also has disadvantages. The reduction in porosity caused by the surface modification with hydrophobic functional groups impairs their CO_2_ adsorption ability.^[^ ^61^ ^]^ Furthermore, high lignin content (cellulose has a moderate hydrophobicity, whereas hemicelluloses have a low hydrophobicity) and heating of the precursor before beginning the pyrolyze (the hydrophilic groups are removed by incrementing the temperature) result in a framework with a high hydrophobic angle.^[^ ^254^ ^]^ In this context, involving nitrogen doping as defects can boost wettability and hydrophilicity. N, as a barrier, appears to prevent further gas uptake in wet conditions.^[^ ^255^ ^]^ The hydrophilicity of the carbon surface is increased by the presence of oxygen‐containing functional groups that are frequently polar. Hydrogen bonds between water molecules and the oxygen atoms on the surface are responsible for hydrophilicity.^[^ ^32^, ^256^ ^]^ The presence of hydroxyl groups on the surface, as well as a large amount of oxygen (O‐C═O, O/C ratios), have been found to boost carbon hydrophilicity, introducing more polarity into the carbon structure.^[^ ^53^, ^85^, ^257^ ^]^ To sum up, the hydrophilicity of the structure arises due to the presence of functional groups and acts as a barrier to gas adsorption in humid conditions.

#### 5.2.5 Metal Doping

**Figure 21:**
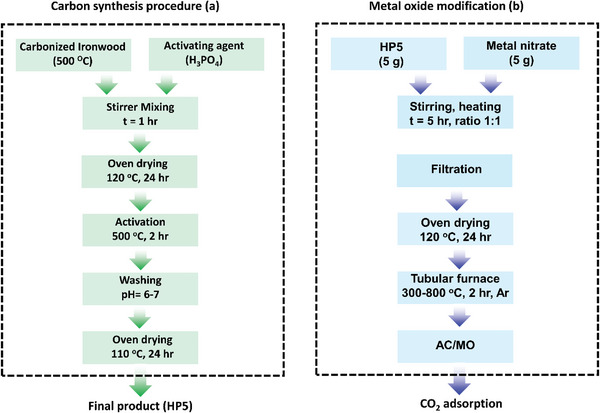
Schematic of porous carbon synthesis using metals nitrate Reproduced with permission^[^ ^259^ ^]^ Copyright 2018, Elsevier.

Nanocomposites, as state‐of‐the‐art materials, have a great potential to be applied as carbon‐capturing materials Nanocomposites are a solid multiphase material, in which one of the phases either has at least one dimension ranging between 1 to 100 nm (such as nanoparticles, carbon nanotubes, and nano‐fibers) or is a nano‐scaled structure (such as nanoporous carbon). Nanocomposites show improved and novel properties that are different from their building blocks. Their constituent components include polymers, ceramics, and metals. Metal/activated carbon (AC) nanocomposites, due to improved reactivity, abundant nanopores, large surface area, and easy synthesis have attracted great attention as favorable adsorbent materials. ACs incorporated with different metals such as Al, Ni, Mg, and Cu or mixed metals have been reported for fast and enhanced CO_2_ sorption.^[^ ^258^ ^]^ In 2018, Nowroozi et al. developed an AC/metal oxides (MOs) nanocomposite for efficient CO_2_ sorption.^[^ ^259^ ^]^ They prepared activated carbon by chemical activation of Persian ironwood biomass using H_3_PO_4_ as an activating agent as depicted in **Figure** **21**. Afterward, they prepared a MOs/AC nanostructured composite through mixed‐MO carbonization. By trying different amounts of activating reagents and loaded metal, the desired MOs were synthesized. It was demonstrated that the AC's textural characteristics were also enhanced greatly. Chemisorption and physisorption mechanisms simultaneously resulted in a higher capacity for CO_2_ uptake in comparison with unmodified ACs. The maximum adsorption capacity (6.78 mmol g^−1^) was achieved at 30  °C and 1 bar, which was 124.5% higher than unmodified ACs’ capacity (3.02 mmol g^−1^).

Recently Pu et al. prepared a high‐efficiency composite of nature‐derived AC/ MgO‐Al_2_O_3_ for dynamic CO_2_ adsorption. They studied the effect of different ratios of loaded MgO on CO_2_ capture. Additionally, they compared the adsorption capability of single‐metal (MgO) and bi‐metal (MgO‐Al_2_O_3_) composites. Bi‐metal MgO‐Al_2_O_3_/ACs composite showed the highest dynamic CO_2_ capture (4.50 mmol g^−1^).

It was concluded that composites of biomass‐derived ACs with MgO‐Al_2_O_3_ can be successfully applied to develop CO_2_ sorbent materials.^[^ ^260^ ^]^ Moreover, polymeric/AC nanocomposites have also been developed for efficient carbon capture. In 2020, Nisar et al. reported a nano‐composite of polysulfone and metal‐activated carbon. Wood sawdust‐derived carbon was activated by either Ni or Co salt. Polysulfone nano‐composite was then prepared by melt mixing technique. The obtained nano‐composite provided enhanced CO_2_ adsorption capacity and it was shown to be energy‐efficient, mechanically robust, and stable under different temperatures.^[^ ^261^ ^]^ In addition, Guo et al. disclosed the physical activation of nanoclay Laponite, resorcinol, and formaldehyde at 800 °C for one hour to create hierarchically organized porous carbon composites utilizing an interfacial assembly technique. High CO_2_/N_2_ selectivity (114.3) at 70 °C could be achieved by carefully adjusting the surface chemistry and pore network of porous carbons using this production method.^[^ ^262^ ^]^ Moreover, Xu et al. developed carbon based oxyhydroxide composites (pristine and ball‐milled biochar/Fe oxyhydroxide) for CO_2_ uptake at 25 °C.^[^ ^196^ ^]^ In general, it seems that although porous carbon composites have high gas sorption capability, they are not able to be used on an industry scale.

Basic metallic elements, such as iron, cobalt, sodium, magnesium, calcium, and their oxides, can influence the performance of materials. Several investigations have shown that highly connected organic/inorganic hybrids have remarkable electrochemical properties for metal doping. However, the significance of atomic metal doping in carbon capture has not been well examined, notably through experiments, although a few theoretical simulations imply that metal doping is beneficial.^[^ ^263^ ^]^ Owing to the interaction between metal and pyridinic nitrogen, where electrons are transferred from metal to pyridinic nitrogen, metal doping appears to cause a shift in the type of nitrogen from pyridinic to pyrrolic. This change in the structure may be beneficial for CO_2_ adsorption. Nevertheless, metal doping decreases S_BET_ owing to pore blockage caused by metal deposition on the material surface.^[^ ^264^ ^]^

**Figure 22:**
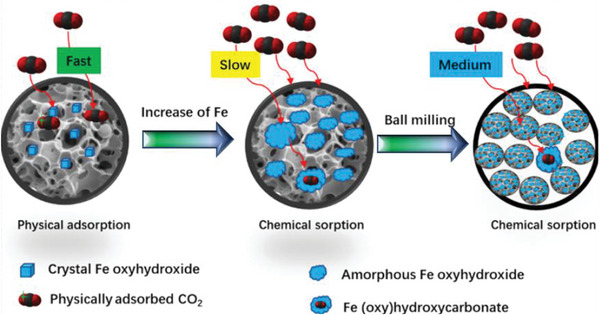
The role of Fe in reducing the kinetic rate of CO_2_ uptake, Reproduced with permission^[^ ^196^ ^]^ Copyright 2020 Elsevier.

Using metal oxides to impregnate porous carbons improves the CO_2_ uptake capability by increasing their basicity.^[^ ^265^ ^]^ Creamer et al. recently synthesized a combination of metal hydroxides and cottonwood biomass to produce metal oxyhydroxide‐biochar composites. It was proposed that metal oxyhydroxides could interact with acidic gases like CO_2_ as they are basic. Their findings verified the hypothesis, with a CO_2_ sorption capacity of 71 mg g^−1^ (3.16 mmol g^−1^) for this composite at 25 °C, compared to 58 mg g^−1^ (2.6 mmol g^−1^) for the unmodified sample under similar conditions.^[^ ^266^ ^]^ Similarly, Lahijani et al. systematically investigated the doping effect of a series of metal atoms, and they found that the inclusion of basic metal sites into the material skeleton improved the sorption of CO_2_ onto the metalized‐biochar in the order Mg > Al > Fe > Ni > Ca > unmodified‐biochar > Na. At 25 °C and 1 atm, Mg‐loaded biochar had a higher CO_2_ uptake (3.66 mmol g^−1^) than unmodified biochar (3.24 mmol g^−1^).^[^ ^267^ ^]^ Zubbri et al. discovered that incorporating magnesium into the skeleton boosted CO_2_ adsorptive capability. In this regard, when compared to pristine biochar (3.06 mmol g^−1^), metalized biochar showed a higher CO_2_ sorption rate (3.42 mmol g^−1^).^[^ ^268^ ^]^ According to Hosseini et al., a combination of the O‐groups and metal ions can produce metal complexes with negatively charged acidic groups. Consequently, negatively charged groups on the surfaces of porous carbon and Cu‐loaded carbon samples were surrounded by Zn^2+^ ions that were sorbed on the substrate surface, facilitating surface diffusion and ion reduction at more favorable locations.^[^ ^269^ ^]^ Moreover, as shown in **Figure** **22**, Xu et al. found that with a higher Fe content, porous carbon showed more CO_2_ adsorption, but the sorption kinetics became lower.^[^ ^196^ ^]^

#### 5.2.6 Temperature and Pressure

External parameters such as temperature and pressure can considerably alter the adsorption capacity of porous adsorbents. It is well acknowledged that the adsorption capacity soars when the temperature drops and the pressure rises, according to Gibbs free energy equation and Le Chatelier's principle. According to the Boltzmann equation, the interaction boost is caused by a rise in the kinetic energy of the gas‐solid molecules engaged in sorption, which enhances molecular interactions while decreasing efficient sorption levels.^[^ ^270^ ^]^ The vibrancy of the gas molecules (kinetic energy) intensifies as the temperature rises, and as a result of these movements, the collision of the molecules with each other is substantially enhanced, resulting in the phenomena of repulsion from the surface. In addition, the exothermic nature of the adsorption reaction leads to the prominent role of temperature in the adsorption rate. Therefore, the rate of adsorption reduces at higher temperatures. As the adsorption temperature increases, the equilibrium time (adsorption saturation point) decreases.

Furthermore, raising the pressure leads the unadsorbed molecules to exert pressure on the adsorbed molecules, causing the force to be transferred to them and their transport to the pores to be accelerated.^[^ ^271^ ^]^ Conversely, in microporous and ultra‐microporous predominated porous materials, the CO_2_ sorption on the surface proceeds via a pore‐filling mechanism instead of layer sorption under high pressures. As a consequence, after the micropores and ultra‐micropores are filled, increasing the pressure will not boost CO_2_ uptake considerably.^[^ ^1a,102244^ ^]^

### 5.3 Selectivity of CO_2_/N_2_

Selectivity is defined as the ratio of the adsorption capacity of CO_2_ to any other gas (e.g., CH_4_, O_2_, N_2_, and NO_x_).^[^ ^195a^ ^]^ The selectivity of CO_2_ over N_2_ is an important criterion for evaluating a solid sorbent because it represents the CO_2_ separation impact in real‐world applications (post‐combustion flue gases, chemical, environmental, and pharmaceutical industries).^[^ ^252^ ^]^ The ideal adsorbed solution theory (IAST) model is frequently used to estimate the binary gas mixture equilibrium using single‐component isotherms at 0 or 25 °C under 0–1 bar (10‐15 vol% CO_2_ and 85–90 vol% N_2_) to calculate the selectivity of CO_2_ over N_2_. Performing a linear fit to the adsorption isotherms (fitted with the Langmuir‐Freundlich equation) at low pressure and computing the ratios of the slopes provides ideal selectivity values.^[^ ^272^ ^]^ The selectivity (S_CO2/N2_) of the IAST is determined by the equation:

(19) $$S_{{\mathrm{CO}}_{2}/N_{2}}=\frac{q_{{\mathrm{CO}}_{2}}q_{N_{2}}}{p_{{\mathrm{CO}}_{2}}p_{N_{2}}}$$

where q_CO2_ and q_N2_ are the sorbed quantities of CO_2_ and N_2_, derived from the single component sorption isotherm and the CO_2_ (0.15 bar) and N_2_ (0.85 bar) partial pressures in the flue gas are denoted by p_CO2_ and p_N2_, respectively.^[^ ^4^ ^]^ Henry's method is another approach for estimating selectivity. A virial equation was used to fit the sorption data. The accompanying equations are used to calculate Henry's method‐based selectivity values:^[^ ^201^, ^273^ ^]^

(20) $$\begin{array}{ccc} \ln\left(P\right) & = & \ln\left(Va\right)+\frac{a_{0}+a_{1}*V+a_{2}*V^{2}+\cdot \cdot \cdot +a_{n}*V^{n}}{T}\\ & & +\;\left(b_{0}+b_{1}V\right) \end{array}$$

where V is the amount of gas sorbed (in mmol g^−1^), P is pressure, T is constant temperature (in K), and a_0_, a_1_, a_2_… and b_0_, and b1 are temperature independent empirical variables. The constant temperature (T) is used to compute Henry's constant (K_H_):

(21) $$K_{H}=\exp\left(-b_{0}\right)*\exp\left(\frac{-a_{0}}{T}\right)$$

The Henry's Law (S_ij_) for selectivity of CO_2_ over N_2_ (i over j) is computed from the following formula:^[^ ^274^ ^]^

(22) $$S_{ij}=K_{Hi}/K_{Hj}$$

**Figure 23:**
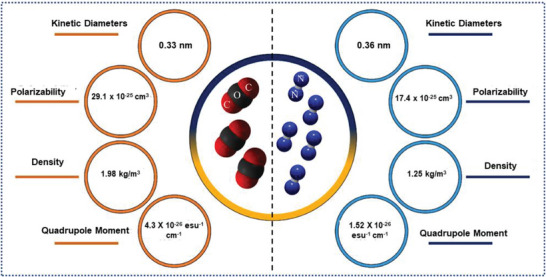
Overview of CO_2_ and N_2_ properties to better understand the gas separation process.

Based on the size/shape exclusion of some components of a gas mixture, the molecular sieving effect is one possible mechanism for adsorptive separation. Besides, the kinetic effect is caused by variations in the diffusion rates of distinct gas mixture components, and the thermodynamic equilibrium effect is caused by preferred adsorbate‐surface or adsorbate packing interactions.^[^ ^275^ ^]^ The intricate interactions between the sorbate and the sorbent, as well as competing sorption between the various species, give rise to selectivity. The higher developed micropore volume as an affecting parameter for sorption will adsorb other competing gases as well, hence a sorbent with a high sorption capacity does not always have a high selectivity. Likewise, an adsorbent with a lower CO_2_ sorption capability may not always have low selectivity because low porosity size may also reject competing components.^[^ ^32a^ ^]^ Moreover, increased selectivity for CO_2_ is dictated by the surface chemistry (doping elements such as N, P, S, and O).^[^ ^276^ ^]^ When it comes to CO_2_/N_2_ separation, the selectivity of CO_2_ over N_2_ progressively grows as pressure rises. Conversely, the selectivity diminishes steadily as N_2_ climbs (Mole fraction of N_2_).^[^ ^7^, ^277^ ^]^ According to the earlier study, CO_2_ sorption appears to be substantially greater than nitrogen sorption, which might be attributable to several variables. The N_2_ and CO_2_ molecules have kinetic diameters of 0.36 and 0.33 nm, respectively. These values are so near that separating them using a size exclusion or molecular sieving process is difficult. However, CO_2_ has a greater quadrupole moment (4.3 × 10^−26^ esu^−1^cm^−1^) and is more polar than N_2_ (1.52 × 10^−26^ esu^−1^cm^−1^), which results in a stronger van der Waals force between CO_2_ and adsorbent than N_2._^[^ ^192^, ^278^ ^]^ Furthermore, CO_2_ (29.1 × 10^−25^ cm^3^) has greater polarizability than N_2_ (17.4 × 10^−25^ cm^3^). Additionally, the CO_2_ molecules are acidic, but the N_2_ molecules are not. As a result, the potential to adsorb on porous surfaces rises, particularly in functionalized situations. CO_2_ molecules can be sorbed in the structure that includes charged species (electronegative), active sites, or functional groups that can interact with gas molecules. The N_2_, on the other hand, lacks extra coulombic attractive forces and is mostly sorbed by conventional dispersion forces.^[^ ^5^, ^279^ ^]^ A comparison of the properties of N_2_ and CO_2_ is shown in **Figure** **23**.

## 6 Simulation Methods

### 6.1 Molecular Simulation

Simulation is the process of creating a model of a real system and performing experiments on it to realize the system's behavior or assess alternative operating techniques. Among simulation methods, molecular simulations are one of the most effective modeling approaches for determining numerous microscopic aspects of complex and sophisticated systems without making any assumptions, such as absolute adsorption capacity, adsorption mechanisms at nanoscale, and surface chemistry, even when tests are unfeasible or unattainable.^[^ ^280^ ^]^ According to the degree of freedom, molecular simulation is separated into two categories: molecular dynamics (deterministic) and Monte Carlo (stochastic). To analyze the behavior of a phenomenon, the MC technique, which is used to research physical and economic systems, uses replicate simulations. In principle, MC refers to any statistical sampling approach that delivers approximate answers to quantitative issues. Conversely, molecular dynamics simulation is a computer‐assisted approach for investigating the microscopic aspects of a system. The Newton equation of motion is the equation solved in the simulation of molecular dynamics for the atoms in the system.

To date, MC is the most fundamental and adaptable tool for analyzing molecular interactions. Nonetheless, it suffers from a high computational cost, making it unsuitable for dealing with time and length scales in many applications. Applying boundary constraints and calculating long‐range Coulombic interactions are similarly difficult.^[^ ^281^ ^]^ At high pressure and low temperature, measuring the quantity of CO_2_ sorption by porous structures needs extremely complicated equipment. Furthermore, several experiments are required to determine the exact function of various parameters such as microporosities, mechanism of CO_2_ capture, and surface chemistry. Due to the presence of nitrogen in the stacks, the IAST law should be used to determine the selectivity of CO_2_ to N_2_. However, these approaches often need experimental isotherms for each of the system's pure components, as well as numerical fitting of the experimental data, which is error prone. The overall accuracy of these strategies is difficult to determine, and for some mixes, less than adequate agreement with experimental data has been reported. Given the issues mentioned, molecular simulation methodologies offer access to a robust theoretical framework based on statistical mechanics that is excellent for researching single/multi‐component adsorption in porous materials and other complicated nanoscale processes.^[^ ^282^ ^]^ To estimate the thermodynamic equilibrium parameters of the gas‐carbon pore system, MC simulations were performed inside the Grand Canonical (GCMC) ensemble. GCMC and MD simulations are frequently performed using the RASPA or Gaussian 09 and LAMMPS packages. Because of the defined arrangement of atoms relative to each other, simulating gas adsorption on porous structures with specified topologies such as graphite and graphene is a simple process. In contrast, due to the irregularity of the pores in porous carbons, such structures are extremely difficult to configure in order to simulate the sorption process. Several simulation studies have been conducted to understand the sorption of single component CO_2_ or multi‐component (CO_2_/CH_4_/N_2_/H_2_) on carbon‐based materials, such as graphene, carbon nanotube (CNT), and graphite. For instance, Kumar et al. simulated sorption of CO_2_/H_2_ with mixture ratios (10:90 and 20:80) using MD at 298 K, and they found that CNT was better than the slit pore to separate CO_2_ (selectivity 100–313) from mixtures at ambient temperature.^[^ ^283^ ^]^ Trinh et al. used classical MD to study the influence of pore width and surface charge in carbon mesoporous (2.5‐5 nm) on CO_2_/H_2_ sorption and selectivity at 300–400 K. They observed that selectivity was largely indifferent to pore width, and that metal contamination, simulated by localized charges inside an electro‐neutral pore surface, increased the sorption selectivity ratio for CO_2_ versus H_2_ while decreasing the diffusion selectivity for CO_2_ versus H_2_.^[^ ^284^ ^]^ Likewise, Aljaddani et al. used MD simulations at ambient temperature to model the separation of a CO_2_ and CH_4_ mixture on a graphite substrate covered by graphene nano‐ribbons.^[^ ^285^ ^]^ Furthermore, Yang et al. investigated the sorption and diffusion characteristics of CH_4_ and CO_2_ in CNTs with preadsorbed water at 300 K and pressures up to 40 bar using GCMC (adsorption isotherms) and MD (dynamic properties). They discovered that at low pressures, relatively tiny pores had high sorption of CH_4_ and CO_2_, and the presence of water promotes CO_2_ uptake in CNTs with large diameters.^[^ ^286^ ^]^

Biomass‐derived porous carbon has a turbostratic and amorphous‐crystalline intermediate structure. Therefore, due to the randomness and irregularity of the porosity, the simulation of this type of structure cannot be fully consistent with the synthesized sample. Based on these experimental findings, bulk porous carbon is defined as an aggregation of randomly arranged cross‐linked finite graphene sheets.^[^ ^287^ ^]^ In another work, Pikunic et al. used the reverse Monte Carlo (RMC) approach to build the porous carbon, generating a radial distribution function (RDF) equivalent to that obtained from experimental measurements.^[^ ^288^ ^]^ Shi predicted multiple porous carbons with varied quench rates using the quench molecular dynamics (QMD) approach with the reactive state summation (RSS) potential. His results showed that the quench rate has a considerable effect on the porous structure, including ring size distributions, PSD, and angle distribution.^[^ ^289^ ^]^

In addition to determining the porosity, other simulating challenges include adjusting the kind of bonds, location of functional groups, and heteroatom quantity, which reduces the percentage of similarity with the synthesized sample. The first step in commencing the simulation process is to create a framework that yields porous carbon. Biase and Sarkisov investigated the sorption of H_2_O and multicomponent mixtures using a developed model of a high S_BET_ porous carbon with pores not exceeding 1 nm (Maxsorb MSC‐30), based on a random packing of small fragments of a corannulene‐like carbon sheet functionalized with hydroxyl groups.^[^ ^290^ ^]^ Surface heterogeneity appears to alter adsorbate accumulation configuration by altering the pore surface's geometry and the surface's charge distribution. Surface functionalities govern sorption as pore width decreases; hence, surface functions play a more vital role in enhancing CO_2_ sorption capacity. Trinh et al. used classical MD simulation at 300 K and up to 40 bar to evaluate the influence of surface charged defects in carbon mesoporous on sorption selectivity for a CO_2_/CH_4_ mixture. They discovered that localized charged defects inside an electro‐neutral pore surface boost CO_2_/CH_4_ separation selectivity and solely interact with CO_2_ molecules. The results reveal that a charged defect of 0.45 electron/atom could yield a very high selectivity of approximately 25.^[^ ^291^ ^]^ Li's group synthesized heteroatom‐doped porous carbons from waste tobacco stem for CO_2_ sorption and utilized GCMC to investigate the role of functional groups and pore structure. They showed that the pore structure and functional groups contribute 62% and 38% of the CO_2_ uptake, respectively. N and O‐doped porous carbons have a stronger impact on CO_2_/N_2_ selectivity because N and O doping improves the electrostatic interaction of porous carbons with CO_2_ molecules.^[^ ^292^ ^]^ Additionally, the same team discovered that the carboxyl and hydroxyl groups on porous carbons are extremely susceptible to CO_2_ sorption. They calculated that these groups and pore structure contribute 37% and 63% of the CO_2_ uptake by GCMC, respectively. They revealed that the oxygen atoms in the COOH group have a negative charge and can provide a stronger electronegativity by gaining electrons from the direct‐connected H or C atoms, whereas the C and H atoms in the COOH group show high electro‐positivity by donating electrons, providing effective CO_2_ sorption sites.^[^ ^217^ ^]^

**Figure 24:**
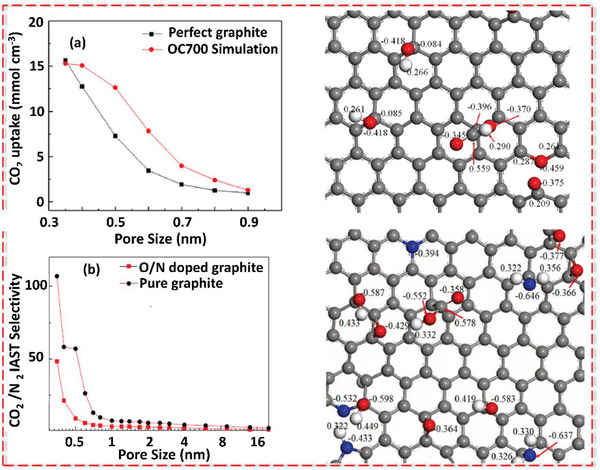
Overall comparison of the results of CO_2_ adsorption and selective separation obtained from simulation with the results obtained from the experimental condition in different porosity sizes. a) Reproduced with pemission.^[^ ^217^ ^]^ Copyright 2020, Elsevier. b) Reproduced with permission.^[^ ^292^ ^]^ Copyright 2021, Elsevier.

In addition to functional groups and pore size, simulation studies have demonstrated that metal doping can boost gas sorption. Using GCMC and DFT, Ma et al. demonstrated that alkali metals, such as Li, Na, and K, might dramatically boost CO_2_ uptake in carbon surfaces. The impacts of the significant electrostatic interaction and the high adsorption energy mostly contribute to the augmentation of CO_2_ sorption. According to the comparison research, the doping of alkali metals into carbon surfaces for CO_2_ capture achieves 8.43–12.46 mmol g^−1^, which is around 2.6–3.8 times that of the non‐doped one.^[^ ^293^ ^]^ In two separate articles, Ma et al. developed GCMC simulation to predict CO_2_ capture and CO_2_/N_2_ selectivity based on pore size onto biomass‐based porous carbons as shown in **Figure** **24** and compared their simulation result with experimental conditions. Moreover, they found that according to simulation, the contribution of pore structure and functional groups was 62% and 38%, respectively.^[^ ^217^, ^292^ ^]^

In another research, Wang et al. demonstrated that the interaction between sorbent and sorbate has a greater impact on sorption at low pressure, whereas the PSD has a greater impact at high pressure by GCMC. The results showed that pore diameters higher than 1 nm are not favorable for increasing CO_2_/CH_4_ selectivity, but ultra‐micropores with pore sizes smaller than 0.7 nm are beneficial to increase selectivity. Furthermore, modified samples with COOH groups displayed an amazing improvement in selectivity as well as a significant electrostatic contribution

at low pressure.^[^ ^294^ ^]^ According to Luo et al., N functional groups have an important role in CO_2_ capture at low pressure up to 0.16 bar; however, at high pressure under 0.16–1 bar, D_pore_ (0.7 nm) has a considerable impact on CO_2_ sorption capacity, which is consistent with earlier studies (D_pore_ smaller than 4 nm is responsible for sorption at high pressures).^[^ ^295^ ^]^

CO_2_ molecules are produced in different ways in the simulation framework, one of which is the TraPPE model and EPM2, which are the three‐site rigid model that accounts for the inherent quadrupole moment of CO_2_ using a partial charge at each site. The partial charges are q_(Carbon)_ = 0.70 and 0.65 e, q_(Oxygen)_ = −0.35 and −0.32 e (e = 1.6022  ×  10^−19^ C), respectively.^[^ ^296^ ^]^ A system can be made up of a matrix (such as amorphous silica, porous carbon, or polymers) and guest molecules (e.g., N_2_, O_2_, and CO_2_). The atoms in the system must interact with other particles in the system in appropriate short‐ and long‐range ways. Non‐bonded interactions include contributions from van der Waals, electrostatic, and other sources. For example, the Lennard‐Jones equation and a Coulombic component are frequently included in the functional form for non‐bonded interactions.^[^ ^297^ ^]^ Sorption in an adsorption system is governed by sorbate‐sorbent and sorbate‐sorbate interactions, with the van der Waals force and the Coulomb force playing essential roles. The van der Waals force as non‐bonded energies is defined using the Lennard‐Jones (LJ) potential model with 12–6 parameters.^[^ ^298^ ^]^

(23) $$E_{\left(r_{ij}\right)}=4\varepsilon_{ij}\left[{\left(\frac{\sigma_{ij}}{r}\right)}^{12}-{\left(\frac{\sigma_{ij}}{r}\right)}^{6}\right]+\frac{q_{i\;}q_{j}}{4\pi \varepsilon_{0}r}$$

where r_ij_ is the intersite distance between atoms i and j, ε_ij_ is the L‐J potential's well depth, and σ_ij_ is the L‐J size parameter. ε_0_ indicates dielectric permittivity of vacuum (8.8543 × 10^−12^ F/m), while qi and qj are the charges of particles i and j in units of e, respectively.^[^ ^280b^ ^]^

In order to build CO_2_, Transferable Potentials for Phase Equilibrium (TraPPE) is a series of non‐bonded molecular mechanics force fields. The term “transferable” implies that same force field properties are used across different molecules to designate a certain contact location. The most often utilized force field, the TraPPE‐UA, has the equation (Eq. 20) form as follows:

(24) $$\begin{array}{ccc} U\left(r^{N}\right) & = & \sum_{j=1}^{N-1}\sum_{i=j+1}^{N}\left[4\varepsilon_{ij}\left[{\left(\frac{\sigma_{ij}}{r}\right)}^{12}-{\left(\frac{\sigma_{ij}}{r}\right)}^{6}\right]+\frac{q_{i\;}q_{j}}{4\pi \varepsilon_{0}r}\right]\\ & & +\;\sum_{\mathrm{angles}}\frac{k_{a}{\left(\theta -\theta_{0}\right)}^{2}}{2}+U_{\mathrm{torsion}} \end{array}$$

where θ, θ_0_, and k_a_ are related to the current bond‐angle, equilibrium bond‐angle, and force factor, respectively. The Q_st_ at infinite dilution is estimated using GCMC's canonical ensemble (NVT) simulation. In the NVT, a single adsorbate molecule is exposed to three sorts of trial moves: translation, rotation, and regrowth. At infinite dilution, the Q_st_ is determined as follows:

(25) $$Q_{st}=RT-\left(U_{\mathrm{total}}^{0}-U_{\mathrm{intra}}^{0}\right)$$

where U^0^_total_ represents the total adsorption energy of a single molecule with adsorbent and U^0^_intra_ represents the intramolecular interaction of a single gas molecule in the bulk phase. V_free_ is the adsorbent's free volume accessible for sorption.^[^ ^299^ ^]^ The cases stated in the simulation of CO_2_ sorption show that the majority of the study has focused on GCMC. There is relatively little research done to simulate gas sorption by MD on biomass‐derived porous carbon. Khosrowshahi et al. reported that N‐pyridinic had a higher ability to adsorb CO_2_ than N‐graphitic.

**Figure 25:**
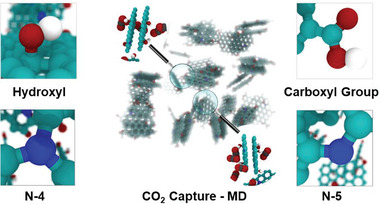
Investigation of effective parameters on CO_2_ uptake by molecular dynamics simulation.

Furthermore, they reported that the simultaneous presence of these two forms of N had a larger influence on CO_2_ sorption than the presence of each separately in the structure as shown in **Figure** **25**. It was also discovered that adding carboxyl groups to the carbon matrix increases CO_2_ uptake by roughly 10%.^[^ ^143^ ^]^ **Table** **5** describes the simulations (MC‐MD) performed on various porous carbon.

**Table 5:** Summary of simulations performed on biomass‐derived porous carbons.

Sample	Sorbate	Simulation	Result	Key tips	Reference
Maxsorb MSC‐30 with OH (CRNL(OH)_2_)	pre‐ and post‐combustion/H_2_O/CO_2_/N_2_/O_2_/H_2_S	GCMC/MuSiC simulation package	S_BET_ = 3236 m^2^.g^−1^ C/O = 7.5 V = 1.28 cm^3^.g^−1^	Favorable locations for CO_2_ coming from the Lennard‐Jones interactions with the extended surfaces of the fragments.	[290]
Porous carbon from waste tobacco stem	CO_2_ capture and CO_2_/N_2_ selectivity	GCMC	CO_2_ adsorption at 25 °C under 1 bar = 2.6 mmol g^−1^ for pure graphite and 4.2 mmol g^−1^ for N‐O doped	The doping generate high positive (0.322∼0.578) and negative charges (−3.58∼‐ 0.646), which can provide high electrostatic interaction,	[292]
Porous carbon from waste tobacco stem	CO_2_ capture	GCMC	CO_2_ adsorption at 25 °C under 1 bar = 2.9 mmol g^−1^ for pure graphite and 4.8 mmol g^−1^ for O doped	pore sizes ranging from 0.35 to 20 nm/ O═C═O bond angle was 180°	[217]
N‐doped porous carbon from Eucalyptus bark	CO_2_ adsorption	GCMC	CO_2_ adsorption density at 25 °C = 8.1 mmol.cm^3^ for 0.6 nm pore size at 100 kPa and 11.9 mmol.cm^3^ for 1 nm pore size at 300 kPa	graphite slit pore model	[295a]
Porous carbon from carbonaceous precursors	CO_2_ adsorption	GCMC	CO_2_ adsorption at 0 °C under 1 bar = 6 mmol g^−1^ CO_2_ adsorption at 25 °C under 1 bar = 4.1 mmol g^−1^	C═O bond length was 1.16 Å / the bond angle O═C═O was 180̊/ 15 000 moves for equilibration/ 25 000 moves for production	[300]
Porous carbon from Date Seeds	CO_2_ Swing Adsorption	GCMC/LAMMPS and Materials Studio software	S_BET_ = 692 m^2^.g^−1^ C/O = 50 V = 0.29 cm^3^.g^−1^	Random packing of graphene‐like sheets + carbonyl groups / TraPPE force field for CO_2_	[301]
Porous carbon from celery	CO_2_ adsorption	MD/LAMMPS	CO_2_ adsorption at 0 °C under 10 bar = 7.95 mmol g^−1^ for optimal sample (Simultaneous presence of carboxyl‐hydroxyl groups and graphite‐pyridinic N)	The average absolute relative error percentage (AARE %) for simulation of the optimal sample is 16%	[143]

### 6.2 Density Functional Theory (DFT)

**Figure 26:**
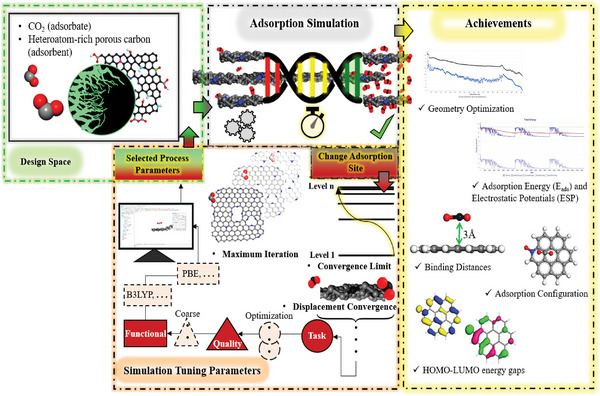
The summary of DFT calculation conducted on porous carbons.

DFT calculation is the most well‐known tool in the main branches of chemistry and material science along with followed challenges and opportunities.^[^ ^302^ ^]^ Although easy accessibility is the key characteristic of DFT, the functional and approximations, as the introduction of usage, make DFT more complicated. It should be emphasized that the DFT is widely applied by solid‐state physics researchers in comparison with the computational chemist community. This soft‐computing route describes geometries and further binding energy prediction of molecules which are regarded as the challenges of DFT. The detailed explanation of chemical reactions, such as the weak interaction of the molecules and the transition state, are crucial factors. In this context, the adsorption mechanisms can be efficiently evaluated using DFT theoretical calculations, which are powered to figure out the multi‐dynamic statistical molecular interactions between the adsorbents and adsorbate. This soft procedure can be employed to elucidate the mechanism of interactions of chemical characteristics on porous carbon surfaces and CO_2_ molecules. Predominantly, DFT calculation is considered the computer‐assisted procedure that helps to affirm experimental CO_2_ adsorption results.^[^ ^303^ ^]^ Though DFT can be easily applied, tuning the multi‐computational/chemical parameters plays a key role in the validation of the calculation. As illustrated in **Figure** **26**, all steps of DFT calculation are summarized by selecting the parameters adapted to the purposes of the subject.

As previously mentioned, synthesizing porous carbon is accomplished based on the various precursors and novel multi‐routes (see Sections 3 and 4), which can synergistically modify the porous carbon physiochemical characteristics (as mentioned in Sections 5.2.2 and 5.2.3). Heteroatom features in porous carbon prominently improve the porous carbon gas storage capability. The CO_2_ molecule as adsorbate and the aimed M‐rich porous carbon (M═N, O, P, etc.) as adsorbent are considered in design space.^[^ ^304^ ^]^ Thus, most applications of DFT focused on the heteroatoms on the surface of porous carbon, which are described as the active adsorption sites. For example, Wu et al. acquired the pristine and N‐doped porous carbon for CO_2_ molecules adsorption. DFT has distinguished the pyridine‐N group as the most effective functionality for CO_2_ adsorption.^[^ ^130^ ^]^ Mostly, the confirmation of the interaction between porous carbon surface and CO_2_ molecules by DFT calculations can be applied based on the binding energy.^[^ ^305^ ^]^ As shown in another alternative research, Wang et al. investigated DFT efficiency on the dual N/O‐doped porous carbon, which contains the oxygen functionalities such as carboxyl (‐COOH), hydroxyl (‐OH), and carbonyl (C═O) and three nitrogen functional groups (pyridinic‐, graphitic‐ and pyrrolic‐nitrogen). The adsorption energy (E_ads_) was achieved in the range of –15.38 to –29.34 kJ.mol^−1^ for all N/O functionalities sites.^[^ ^57a^ ^]^ The adsorption energies calculated through Gaussian code and followed DFT calculations dealt with various exchange‐correlation functions. The equation for *E_ads_* of single gas molecules is formulated based on DFT total energies as follow:

(26) $$E_{\mathrm{ads}}=E_{\mathrm{sub}}+E_{\mathrm{gas}}-E_{\mathrm{sub}+\mathrm{gas}}$$

E_sub_, E_gas,_ and E_sub+gas_ are the total energies of the substrate, CO_2_ molecule, and gas absorbed systems, respectively. The positive adsorption energy (E_ads_ > 0) demonstrates an ideal interaction between the gas molecule and substrate.

DFT calculation can be applied to obtain a deep insight into the CO_2_ adsorption sites on porous carbon. So‐called DFT can be a useful guide to exhibiting intermolecular interactions and electrostatic potentials (ESP). ESP promotes identifying the region of positive and negative potential with the probability of attractive interactions occurrence. Wang et al. obtained the DFT calculation to deliver insight into the CO_2_ capture on N‐C sites (graphitic‐N) to demonstrate ESP.^[^ ^192a^ ^]^ The electron density difference is obtained from the following equation:

(27) $$\Delta \rho =\rho_{\left(AB\right)}-\left(\rho_{\left(A\right)}+\rho_{\left(B\right)}\right)$$

Δρ demonstrates the electron density difference, ρ_(*A*)_, ρ_(*B*)_ and ρ_(*AB*)_, represent the electron densities of the sorbent, the adsorbate, and the adsorption system, respectively. **Table** **6** comprehensively presents the DFT calculation performance carried out on various porous carbon and gaseous adsorbate.

**Table 6:** The summary of DFT calculation conducted on porous carbons.

Adsorbent	Sorbate	Simulation	Simulation Description	The binding site of adsorption	Result	Reference
Nitrogen‐modified porous carbon from sugarcane	CO_2_ and N_2_ adsorption	DFT at B3LYP/6‐31G	DFT is accomplished to optimize the geometries of porous carbon interaction, adsorption energy, and tendencies of CO_2_ and adsorbents.	C═C C‐N	E_ads_ for CO_2_ obtained −49 to ‐52 Kcal mol^−1^ with binding distances of 3.2 and 3.7 Å.	[306]
N‐doped and ‐undoped porous carbons from glucose	CO_2_ uptake	DFT	Hydrogen‐bonding interaction of CO_2_ with functionalized carbon surface.	‐C═O ‐C‐O ‐COOH ‐OH Pyridinic‐N Pyrrolic‐N Graphitic‐N	The CO_2_ adsorption capacity is acquired at 5.01 mmol g^−1^ with a binding distance range of 2‐2.6 Å.	[24c]
Porous carbon	CO_2_ and N_2_ adsorption	DFT‐ PBE	The inherent difference in electronic structures, geometries, and the adsorption energies exhibit the preference for CO_2_ uptake.	C‐C (Three binding sites: hexagonal center, above of carbon atom and C‐C bond center.	The microporous edges of graphitic porous carbon offer the optimal CO_2_ adsorption within (0.43 mmol⋅g‐1) by induced dipole interaction.	[307]
Algae‐based N‐doped porous carbon	CO_2_ capture	DFT‐B3LYP	DFT calculation aimed to deliver deep insight into the CO_2_ adsorption on N‐C sites and demonstrate intermolecular interactions, and electrostatic potentials (ESP).	Graphitic‐N	The E_ads_ achieved −13.6 and −6.9 kJ mol^−1^ for N‐doped and free carbon framework.	[192a]
Nitrogen and oxygen (N/O) dual doped porous carbon (potato starch‐based)	CO_2_ capture	B3LYP/6‐311++G(d, p)	The prominent role of heteroatom‐doped porous carbon (Pyridine) in CO_2_ physisorption.	Single O‐doped and dual N/O co‐doped carbon surfaces	E_ads_ resulted in −6.6, −14 to −27.1 and −14.3 to −22.6kl mol^−1^ for porous carbon, single O and N doped, respectively. Also, for N/O co‐doped porous carbon obtained −22 to29 kJ mol^−1^.	[130]
N/O co‐doped porous carbon derived from biomass	Low‐pressure CO_2_ capture	B3LYP/6‐311++G(d, p)	Evaluate the E_ads_ of CO_2_ on carbon surfaces and explore the molecular‐level mechanism of CO_2_ capture.	N/O functional groups sites (Amine, Pyridine, Pyrrole, Carboxyl, etc.)	E_ads_ for simple porous carbon and pyridine group −5.4 and 21.4 kJ mol^−1^. O groups exhibited lower |E_ads_| compared to N groups (9.7 to 17 and 16 to 21.4, respectively).	[239a]
N/O co‐doped porous carbon	CO_2_ adsorption	DFT‐MO/6‐31G (d, p)	N‐rich porous carbon surface and CO_2_ were calculated to gain the CO_2_ adsorption patterns, positions, and energies.	(i and ii). Central plane and edge of the pure carbon. (iii, iv, v, vi, vii). The pyrrolic‐N doped, carboxide (−C═O), ether groups (−C−O−C), hydroxyl (–OH), and carboxyl groups (−COOH) co‐doped porous carbon surface respectively.	E_ads_ for all seven adsorption sites ranging between −15.38 to −29.34 kJ mol^−1^.	[57a]
Porous carbon	CO uptake	DFT‐PBE	DFT calculations elucidated that the presence of tiny and well‐dispersed metal template clusters induces superior CO adsorption efficiency	Center of porous carbon	Electrostatic potential evaluated and E_ads_ achieved in −2 to ‐35 kcal mol^−1^.	[308]
Porous carbon	CO_2_ adsorption	DFT at B3LYP/6‐31G(d, p)	Evaluating the adsorption energies	Center and edge of porous carbon	It revealed that E_ads_ for un‐/ and modified ACs were −50.74 and −65.21 kJ mol^−1^, respectively	[309]
Porous carbon (casein)	CO_2_/N_2_ selectivity	DFT at B3LYP/6‐31G(d, p)	Determine the functional groups possess lower binding energies which donate a prominent role to design novel material structures and optimization of the adsorption performances.	Center and edge of porous carbon	The Pyridinic‐N and ‐OH/‐NH_2_ species make a supreme contribution to the ultra‐high CO2 uptake.	[310]
Heteroatom‐rich porous carbon	CO_2_ adsorption	DFT‐(GGA‐BLYP)	Observe the capacity of pure and ‐C═O, ‐OK, and ‐COOK group‐functionalized porous carbon surfaces.	Above porous carbon surface	The result exhibited that the ‐C═O, ‐OK, and –COOK functional groups perform higher CO_2_ adsorption heats (−6.5,−22.13, and −24.58 kJ mol^−1^) than the pure porous carbon (−1.93 kJ mol^−1^).	[311]
Asphaltene‐based porous carbon	CO_2_, H_2_S, N_2,_ and CH_4_ uptake	DFT‐M062X	Observing the critical role of nitrogen (and sulfur) defects in enhancing the adsorption capacity of the CO_2_ molecules.	Above porous carbon surface	E_ads_ and HOMO‐LUMO energy gaps obtained −33.64 kJ mol^−1^ and 2 eV, respectively.	[312]
Coal‐derived porous carbon	CO_2_ and CH_4_ adsorption	DFT‐PBE	Various 6‐ring aromatic clusters carbon models were used to simulate surfaces of coal‐based porous carbon.	Above porous carbon surface	Adsorption of CO_2_ onto carbon was stable and the adsorption energy was −18.16 kJ mol^−1^.	[313]
Carbon nanosheet	CO_2_ adsorption	DFT‐PBE	Survey the adsorption and desorption energies of multiple CO_2_ molecules on carbon nanosheet.	Above porous carbon surface	‐0.2 to −4.122 eV in with various charge states of nanosheet.	[314]
Functionalized‐graphite surfaces	CO_2_ adsorption	DFT‐PBE	Evaluate the adsorptive ability of M‐doped (M═N, P, S, and O) graphite surfaces for CO_2_ molecules.	Above porous carbon surface	the adsorption energy achieved −14.10 to 34.42 kJ mol^−1^.	[315]
Waste‐based porous carbon	CO_2_ uptake	DFT	CO_2_ uptake on nitrogen‐containing functional groups improves multi‐Lewis acid‐base, electrostatic, and hydrogen‐bonding interactions.	Three sites of above, edge, and hydrogen‐bonding interactions.	E_ads_ for pure graphite = 2.03, 3.59 ad 11.81 kJ mol^−1^, Pyridinic‐N = 3.83, 13.83 and 15.37 kJ mol^−1^, Pyrrolic‐N = 9.2, 9.61 and 13.24 kJ mol^−1^ and Graphitic‐N = 5.21, 7.81 and 19.61 kJ mol^−1^ in three sites for four N‐functional groups, respectively.	[316]
N‐functional rich porous carbon	CO_2,_ N_2_ capture	DFT	Optimization geometry of CO_2_ adsorption and E_ads_ for ideal CO_2_ adsorption in N‐functionalized surfaces.	Adsorption sites are considered from the edge and above porous carbon surfaces.	E_ads_ for pristine porous carbon is ∼ −0.1 eV. The highest E_ads_ performed (−0.224 to −0.218 eV) for pyridone and pyridine groups.	[317]
Porous carbon	CO_2,_ N_2_ capture	DFT	Performed to calculate the adsorption status and heat of CO_2_ and N_2_ molecules within microporous carbon with a pore‐diameters range of 0.5 nm to 2.0 nm.	Center of micropore	‐18.9 to 29.6 kJ mol^−1^ in the range of 1 to 2 nm pore and 89.7 for 0.5 nm pore.	[6]

### 6.3 Machine Learning Overview

Machine learning (ML) has developed into a crucial technique for effectively evaluating massive volumes of data in a range of industries in recent years. ML techniques are becoming a crucial tool for studying these “big data” issues as high‐fidelity data sets are becoming more and more accessible for applications in various fields.^[^ ^318^ ^]^ Big data science is based on the premise that if you have a lot of data, you might be able to find statistically meaningful correlations that are linked to certain attributes or occurrences. In 1959, Arthur Samuel was one of the first to use the phrase “machine learning” to describe the methods he created to train a computer to play checkers game.^[^ ^319^ ^]^ Developing novel porous carbons for CO_2_ capture is now being intensively studied. To analyze all precursor materials, the number of conceivable techniques for the synthesis of porous carbons is simply too great. For a long time, adsorbent synthesis has only relied on experimental expertise. Once part of these scientific findings was generalized in the form of conceptual approaches, significant progress was quickly accomplished. Instead of doing time‐consuming and challenging laboratory trials, a mathematical prediction model is advised more straightforwardly. As a result, adsorption is a complicated process that requires thorough theoretical description. Using big data to teach computer ideas might be an intriguing way to investigate some of these issues.^[^ ^1^, ^320^ ^]^ The ML approach from the synthesis of porous carbons and CO_2_ process adsorption data to prediction and analysis, regardless of the learning method or purpose, could be separated into the following design pathway as follows:

- Understanding the adsorption phenomenon and the adsorption process is important. If we want to capture CO_2_ in porous carbons, for example, the key performance parameter is the adsorption capacity, which can be calculated directly from experimental adsorption isotherms at a particular temperature and pressure.^[^ ^1a^ ^]^ In wider words, knowing the phenomenon aids us in guiding data collection and processing. The issue description is essential because it influences the procedures for model evaluation, selection, and explanation.
- To learn from, ML requires data. It is imperative to ensure that we have adequate training data. More suitably, the data needs to be trustworthy and sufficiently span the design area we want to investigate. After we have collected a data set, we will move on to data selection. Training data is frequently chosen randomly from a vast collection of training points. However, this is not always the ideal option because the resources are not always evenly dispersed.^[^ ^321^ ^]^
- The next part of the processing is the selection of algorithms (supervised,^[^ ^322^ ^]^ unsupervised,^[^ ^323^ ^]^ and reinforcement learning^[^ ^324^ ^]^), which are divided into three categories with fuzzy borders. In this review, we will just review supervised learning. Supervised learning is the most extensively used approach and the subject of this review. Here, one may find qualities that characterize a material as well as the labels that go with them for prediction parameters. The computation of characteristics, which can subsequently be input into a model to create a prediction, is a popular use case.

**Figure 27:**
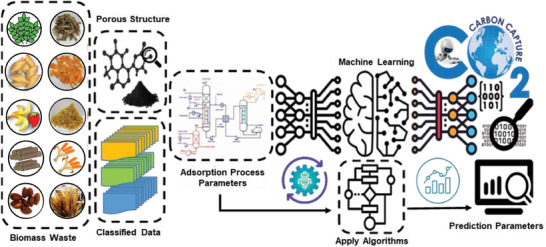
Schema of the supervised ML algorithm for CO_2_ capture using natural porous carbon adsorbents.

Overview of the supervised ML algorithm for collecting data from natural precursors, big data on natural porous carbon adsorbents and the CO_2_ adsorption process, along with specifying the network parameters and obtaining the prediction matrix is shown in **Figure** **27**. ML is a branch of artificial intelligence (AI) whose objective is to create algorithms that can learn from data on their own. In order to make informed decisions, an artificially intelligent agent must be able to detect things in its environment and forecast the behavior of its environment. As a result, ML approaches tend to focus on prediction rather than estimate.^[^ ^318b^ ^]^ In particular, how can we utilize experimental data to estimate CO_2_ adsorption from various adsorbents?

#### 6.3.1 Machine Learning IN THE Adsorption Process and Synthesis

In the case of process adsorption, ML approaches have been used to investigate a number of choices to determine the most cost‐effective process scheme, including selecting a porous carbon as an adsorbent and constructing the requirements of the process. We emphasize the necessity for the development of effective ML‐based approaches for quick screening, as well as the exploration of these choices. For adsorption process operations, we also highlight attractive future prospects for ML in CO_2_ capture processes. Overall, we present a summary of ML in CO_2_ capture that should be useful for research scientists on the computational frontiers of this field who are interested in using this advanced strategy.

Today the use of processing technologies to extract big data from experimental data is being investigated. Material design that meets target property criteria, as well as synthesis stages to manufacture target materials, looks to be within reach, either through learning methodologies or sophisticated generative algorithms. The computational and physical lab infrastructures used to produce materials data are likewise being transformed by ML principles.^[^ ^325^ ^]^ For example, to enhance catalyst performance, researchers used ML to forecast viable electro‐catalysts. Their findings have shown that adopting an intermediate‐binding‐optimization and reaction electrolyte optimization technique for multi‐carbon synthesis through CO_2_ electro reduction might lead to multi‐metal catalysis that outperforms single‐component catalysts.^[^ ^326^ ^]^ Selecting a solid adsorbent and constructing a process are the first steps in developing an adsorption‐based process after identifying the source and sink.^[^ ^327^ ^]^ To construct structural property linkages inside datasets and promote the identification of novel materials with the required capabilities, ML algorithms must be developed.^[^ ^328^ ^]^ The development of such material ML algorithms is especially helpful because they can be used to forecast the efficiency of any arbitrary or hypothetical adsorbent as long as the implemented computational adsorption model can adequately describe the equilibrium adsorption isotherms of CO_2_ for that material.^[^ ^327^ ^]^ TSA, PSA, and Temperature‐vacuum swing adsorption (TVSA) are all examples of cyclic and gas‐solid adsorption technologies for CO_2_ separation. All of these processes may be created by mixing various cycle combinations. A broad range of adsorption processes could be constructed in a PSA process using a mix of cycles such as adsorption, regeneration, and so on. Based on the modeling, solutions for a system of partial differential equations (PDEs) regulating mass, momentum, and heat‐transfer phenomena are required to model such a cyclic process. Several recent research has used ML to address this challenge. For example, Subraveti et al. developed substitution‐aided optimization to forecasting the cycle S.S., lowering the cost of solving systems of PDEs. On a complicated eight fixed‐bed PSA process, the model's precision and dependability were shown, which bodes well for the future use of machine learning for process modeling and optimization.^[^ ^329^ ^]^ The adsorption process design could be applied by ML, within TSA, and PSA. The methods provided by ML science can be helpful for these optimizations. Sun et al., for example, constructed a DNN model called SorbNet that can predict entire isotherm, mixing, and equilibrium features based on simulation data. They were able to create a continuous isotherm function using this method, which was then utilized to improve a chemical process.^[^ ^330^ ^]^ Researchers have focused on the fabrication and synthesis of biomass‐based carbon adsorbents using precursor type parameters, pyrolysis temperature, and synthesis conditions.^[^ ^1a^ ^]^ Predicting synthesis along with predicting the operating conditions of carbon‐based adsorbents at different temperatures and pressures is one of the applications of ML.^[^ ^331^ ^]^ Much research aimed at designing improved adsorbent materials for CO_2_‐capture systems employed simple indicators based on material attributes such as efficiency, selectivity, and heat of adsorption.^[^ ^332^ ^]^ Even though these measurements permitted for a preliminary evaluation, this has been well understood that they do not represent our end objective, which is a cost savings of the CO_2_ adsorption process; as a result, so many current researches that integrate process simulation and optimization for measuring performance have been published.^[^ ^34b,210327333^ ^]^ To predict CO_2_ adsorption capacity and study the effects of pore structure, chemical properties, functional group, and adsorption conditions on CO_2_ adsorption performance, 1594 CO_2_ adsorption datasets were used by Ma et al. in their analysis. At 0.0–0.15 bar, the N_2_ groups of porous carbon have the biggest impact on CO_2_ capture, while at 0.15–1 bar, ultra‐micropores have the biggest impact.^[^ ^334^ ^]^ ML models, including separation techniques based on the membrane, adsorption, absorption, chemical looping, and storage, were used by Gupta and Li to analyze several CO_2_ collections, storage, transportation, and utilization processes. They labeled hybrid processes as such when a variety of CO_2_ capture and utilization approaches could be used in synergy.^[^ ^335^ ^]^ To track the critical operational parameters for the CO_2_ collection process, Wang et al. proposed the ML model utilizing the dataset from soft sensors.^[^ ^336^ ^]^ Using dynamic operational data from a simulator, a substitute intelligent model for this monitoring strategy was discovered. According to simulation results on a large‐scale monoethanolamide‐based CO_2_ capture process model, the proposed monitoring method could accurately predict clean gas CO_2_ concentration and lean or rich solvent loading over a wide operational range, despite the presence of noises and monitoring faults. **Table** **7** contains detailed information and an overview of previous research studies on evaluating the ML approach for CO_2_ capture versus porous carbon synthesis.

**Table 7:** Summary of CO_2_ capture and porous carbon synthesis simulation work using ML approach.

Reference	Adsorbent specification	Algorithm	R^2^	Remark
[34b]	CO_2_ adsorption datasets are 6244 from 155 different porous carbon materials.	Random Forest (RF)	R^2^ > 0.9 For Test	According to the RF algorithm, the volumes of mesopore and micropore had a significant influence at low pressure (0.1 bar); at 1 bar and 0 °C, the R^2^ value between ultra‐micropore volume and CO_2_ uptake quantity was up to 0.715.
[33]	CO_2_ adsorption as a factor of textural and compositional properties was studied using 527 datasets which used from the porous carbons from bio‐waste.	Gradient Boosting Decision Trees (GBDT)	R^2^ > 0.98	Biomass waste‐derived porous carbons‐based CO_2_ adsorption, variable content highlighted the communication of adsorption parameters, textural qualities, and compositional features in the order of precedence, successfully directing the synthesis of porous carbons for CO_2_ adsorption applications.
[338]	For the CH_4_ intake, 1774 datasets have been were gathered from 40 recently published articles, while 1020 datasets for the CO_2_ capture have been were referred to earlier work.	Multi‐Layer Perceptron (MLP)	R^2^ > 0.99	The data was used to train machine learning models, which used specific surface area, micropore volume, mesopore volume, temperature, and pressure as input variables and gas uptake as an output for predictions. By training the MLP model with data taken from PC literature for CO_2_ uptake, it was also extended to investigate CO_2_/CH_4_ selectivity.
[339]	1948 datasets from engine lubricant spectral analysis have been were used.	MLP model (algorithm: trainbr, trainlm)	R^2^ > 99.38	The effect of N/O functional groups of activated carbon material was modeled by ANN
[340]	1138 dataset CO_2_ adsorption have been collected, input parameters: (S_BET_, V_meso_, V_micro_, T, and P)	Deep Neural Network (DNN)	R^2^ > 0.96	Model for screening porous carbons and predicting CO_2_ and N_2_ adsorption, and also CO_2_/N_2_ selectivity at 298 K. CO_2_ and N_2_ adsorption considered at 0.15 bar and 0.85 bar, respectively.
[34a]	1000 dataset, and the input signals were S_BET_, V_micro_, and V_meso_, as well as the adsorption process (pressure and temperature) and the output was CO_2_ adsorption.	Deep Neural Network (DNN)	R^2^ > 0.99	CO_2_ adsorption conditions, mesoporous carbons at pyrolysis temperatures of 450, 600, and 8508 C were selected as the expected samples (25 C and 1 bar).
[341]	940 datasets, input parameters: at N_2_ adsorption (77K) porosity of porous carbons; and output parameters: CO_2_ adsorption at ambient temperature and pressure.	Convolutional Neural Network (CNN)	R^2^ > 0.99	The N_2_ isotherm at 77 K was utilized as direct input (providing shape features for porosity) to train convolutional ANN to predict gas separation using porous carbons (with CO_2_/N_2_ as a dataset).
[263]	CO_2_ adsorption dataset have been applied at 25 C and under 1 bar	MLP model (algorithm: trainlm)	R^2^ > 0.99	The highly porous carbon, with a S_BET_ 1630 (m^2^. g^−1^) was synthesized
[1a]	Several porous carbon syntheses experiments from different biomass at different temperature, from 35 publications have been used as dataset (421 dataset)	ANN such as MLP and RBF models (algorithm: trainlm, trainscg, trainbr, Gaussian)	R^2^ > 0.99	The input and output parameters were Type of precursors, activators, pyrolysis temperatures, pour volumes, adsorption pressure and temperature, and output parameters are BET and CO_2_ adsorption capacity.
[342]	Activation temperature, surface area, pore volume, pore diameter, I_D_/I_G_, current density and the specific capacity are input and output parameters of porous carbon.	Decision tree regression (DT) Linear regression (LR), Support vector regression (SVR),and MLP	R^2^ > 0.9868	To anticipate particular capacitances, four machine learning methods were employed to create quantifiable correlations between synthesis parameters, structural and electrochemical properties of materials.
[343]	1288 dataset, Framework density, average pore size, surface area, N content, average absolute value of charge and the range of charge are input parameters and gas adsorption/selectivity are output parameters.	RF, SVM, extreme Gradient Boosting (XGBoost), and DNN	R^2^ > 0.99	The N_2_ concentration has less of an impact on capacity and selectivity than structural characteristics, according to this report's modeling, but nitrogen doping may boost CO_2_ adsorption and separation vs N_2_ and CH_4_ selectivity in Nano pores with pore sizes near to gas molecules.
[344]	190 dataset of CO_2_ adsorption density on the porous carbon	Multi‐Layer feed‐forward ANN (MLFNN)	R^2^ > 0.99	Temperature and CO_2_ partial pressure were input parameters vs CO_2_ adsorption as a function of thermodynamic conditions was output parameter. Levenberg‐Marquardt back‐propagation and a Bayesian regularization algorithms were used.

#### 6.3.2 Machine Learning Outlook

ML techniques for process design and optimization are only getting started, and more work is needed to construct models consistent with the physics of heat transfer rate and mass transfer to establish a physics‐informed ML method for adsorption. Furthermore, ML methods and architectures that combine adsorption processing parameters would provide for assessing a large variety of processes for specific porous materials. Adsorption‐based processes may be rationally designed with more scientific information in the form of adsorbent characteristics and adsorption process phenomena.^[^ ^337^ ^]^ Techniques for carefully assessing a variety of algorithms and bias means of assessing error in ML projections and how certain errors continue to spread through the entire development process, and algorithms for based multi‐objective searches are just a few of the thrilling directions that really should be established.

## 7 Summary and Future Prospective

The role of CO_2_ emissions from fossil fuel combustion in driving global warming and climate change is widely recognized, leading to extensive research endeavors focused on developing low‐cost and high‐efficiency sorbents for post‐combustion CO_2_ uptake. Natural products‐derived porous carbon has garnered significant attention as a solid sorbent for gas storage due to its highly developed porosity, low cost, high tolerance, and ecologically benign character. This review provides a comprehensive summary of the latest advancements in biomass‐derived porous carbon for CO_2_ sorption, covering various aspects ranging from natural precursor selection through adsorption modeling. The following aspects are thoroughly explored in this review:

- The first stage in porous carbon synthesis involves selecting a suitable precursor based on its intrinsic composition. This review classifies the different types of natural resources along with the traditional and advanced activation/pyrolysis methods employed. Recently, researchers have studied the advanced techniques that enable the production of porous carbons with tunable porosity, addressing the limitations of conventional techniques. Furthermore, a comprehensive examination of the synthesized material encompasses the characterization of its components and careful analysis of all aspects. By delving into these investigations, a thorough understanding of the final synthesized material can be attained.
- The adsorptive capability of the pristine porous carbon is found to be moderate. As a result, in order to enhance the adsorption rate, modifications to the structure and surfaces are necessary. By thoroughly examining these elements, a deeper understanding of the mechanisms at play can be made and effective strategies for optimizing the adsorption performance of porous carbon materials can be identified.
- The analysis of adsorption data demonstrates conflicting effects of several parameters, making it challenging to ascertain definitively which variable plays the most crucial role in sorption. Moreover, accurate determination of the adsorption quantity under specific conditions necessitates the utilization of sophisticated equipment, which is of utmost importance. To address these challenges, modern approaches such as simulation methods (MD, MC, and DFT) and machine learning techniques will be indispensable in resolving these complexities and providing valuable insights. These advanced methodologies offer promising avenues to overcome the uncertainties associated with parameter effects and enable a more comprehensive understanding of adsorption processes. The reliance on extensive experimental investigations to elucidate the role of surface functionality can be reduced by using various simulation methods and machine learning techniques. These approaches enable the measurement of adsorption types and quantities in conditions that would otherwise be unfeasible. By analyzing the resulting isotherms, it becomes possible to approximate the nature of solid‐gas interactions. As a result, these advanced computational methods offer a valuable means to reduce the need for a large number of experimental experiments in understanding surface functionality.

Despite the challenges involved in utilizing porous carbon materials as adsorbents for CO_2_ capture, we firmly believe that with a deeper understanding of carbon chemistry, advanced porous materials will emerge as a crucial solution to the CO_2_ problem. Furthermore, by employing natural precursors, we can reduce the additional costs associated with the incineration of environmental wastes. As a result, biomass‐derived porous carbons in various forms are poised to play a significant role in contemporary living, facilitating the provision of sufficient energy, seamless living, and a safe environment.

Although porous carbons are promising for CO_2_ capture, their adsorption capacity is often compromised at real flue gas conditions. Their CO_2_/N_2_ selectivity is another cause of concern. These limitations, however, can be addressed through modification with various amine‐based materials. Even though significant amount of literature is available which demonstrates the potential of porous carbons for CO_2_ capture, lot more needs to be done to take the present day state‐of‐the‐art technology to its fruition. Along that goal, immense concerted efforts are needed. For example, testing needs to be carried out in a real operational environment to achieve the technology readiness level (TRL). One of the critical challenges with porous carbons to enhance their practical application is to control and narrow down the pore size distribution which would lead to their high and selective CO_2_ adsorption at real flue gas conditions. Addressing this challenge could open potential for the development of prototypes which can later go on to achieve the higher TRLSs. Despite challenges, research on porous carbons for CO_2_ capture remains an active and ever‐evolving pursuit. A wealth of literature focuses on novel material design, pore size tuning, surface functionalization, hybridization, kinetics, thermodynamics, regeneration, recycling, and scale‐up. However, for large‐scale practical realization and adoption as a viable technology, thorough techno‐economic assessments are essential.

## Conflict of Interest

The authors declare no conflict of interest.

## References

[1] a) H. Mashhadimoslem , M. Vafaeinia , M. Safarzadeh , A. Ghaemi , F. Fathalian , A. Maleki , Ind. Eng. Chem. Res. 2021, 60, 13950;

[2] G. Singh , R. Bahadur , J. M. Lee , I. Y. Kim , A. M. Ruban , J. M. Davidraj , D. Semit , A. Karakoti , H. Ala'a , A. Vinu , Chem. Eng. J. 2021, 406, 126787.

[3] W. Zhang , Y. Bao , A. Bao , J. Environ. Chem. Eng. 2020, 8, 103732.

[4] E. Jang , S. W. Choi , S.‐M. Hong , S. Shin , K. B. Lee , Appl. Surf. Sci. 2018, 429, 62.

[5] D. Saha , M. J. Kienbaum , Micropor. Mesopor. Mater. 2019, 287, 29.

[6] S.‐C. Qi , Y. Liu , A.‐Z. Peng , D.‐M. Xue , X. Liu , X.‐Q. Liu , L.‐B. Sun , Chem. Eng. J. 2019, 361, 945.

[7] B. Yuan , X. Wu , Y. Chen , J. Huang , H. Luo , S. Deng , Environ. Sci. Technol. 2013, 47, 5474. 23688273 10.1021/es4000643

[8] a) S. Wang , X. Liu , Appl. Energy 2017, 200, 204;

[9] X. Zhang , I. Elsayed , X. Song , R. Shmulsky , Sci. Total Environ. 2020, 748, 142465. 33113689 10.1016/j.scitotenv.2020.142465

[10] D. Li , J. Zhou , Z. Zhang , L. Li , Y. Tian , Y. Lu , Y. Qiao , J. Li , L. Wen , Carbon 2017, 114, 496.

[11] K. S. Lakhi , G. Singh , S. Kim , A. V. Baskar , S. Joseph , J.‐H. Yang , H. Ilbeygi , S. J. Ruban , V. T. Vu , A. Vinu , Micropor. Mesopor. Mater. 2018, 267, 134.

[12] Y. Zhou , P. Tan , Z. He , C. Zhang , Q. Fang , G. Chen , Fuel 2022, 311, 122507.

[13] Q.‐J. Wu , J. Liang , Y.‐B. Huang , R. Cao , Accts. Chem. Res. 2022, 55, 2978. 10.1021/acs.accounts.2c00326 36153952

[14] S.‐L. Hou , J. Dong , X.‐Y. Zhao , X.‐S. Li , F.‐Y. Ren , J. Zhao , B. Zhao , Angew. Chem., Int. Ed. 2023, 62, e202305213. 10.1002/anie.202305213 37170958

[15] I. Sullivan , A. Goryachev , I. A. Digdaya , X. Li , H. A. Atwater , D. A. Vermaas , C. Xiang , Nat. Catal. 2021, 4, 952.

[16] S. Fang , M. Rahaman , J. Bharti , E. Reisner , M. Robert , G. A. Ozin , Y. H. Hu , Nat. Rev. Methods Primers 2023, 3, 61.

[17] a) S. Zhu , B. Zhao , H. Zhang , Y. Su , J. Environ. Manage. 2023, 328, 117020; 36527800 10.1016/j.jenvman.2022.117020

[18] K. S. Lakhi , W. S. Cha , S. Joseph , B. J. Wood , S. S. Aldeyab , G. Lawrence , J.‐H. Choy , A. Vinu , Catal. Today 2015, 243, 209.

[19] Y. Guo , C. Tan , J. Sun , W. Li , J. Zhang , C. Zhao , Chem. Eng. J. 2020, 381, 122736.

[20] a) C. Chen , Y. Yu , C. He , L. Wang , H. Huang , R. Albilali , J. Cheng , Z. Hao , Appl. Surf. Sci. 2018, 439, 113;

[21] a) K. Ramadass , C. I. Sathish , A. Johns , S. J. Ruban , G. Singh , K. S. Lakhi , A. M. Almajid , T. Belperio , A. Vinu , J. Nanosci. Nanotechnol. 2019, 19, 7892; 31196305 10.1166/jnn.2019.16751

[22] a) K. S. Lakhi , D. H. Park , K. Al‐Bahily , W. Cha , B. Viswanathan , J. H. Choy , A. Vinu , Chem. Soc. Rev. 2017, 46, 72; 27809326 10.1039/c6cs00532b

[23] a) D. H. Park , K. S. Lakhi , K. Ramadass , M. K. Kim , S. N. Talapaneni , S. Joseph , U. Ravon , K. Al‐Bahily , A. Vinu , Phys. Chem. Glasses: Eur. J. Glass Sci. Technol., Part B 2017, 23, 10753; 10.1002/chem.201702566 28677823

[24] a) G. Singh , K. Ramadass , J. M. Lee , I. S. Ismail , M. Singh , V. Bansal , J.‐H. Yang , A. Vinu , Micropor. Mesopor. Mater. 2019, 287, 1;

[25] a) G. Singh , S. Tiburcius , S. M. Ruban , D. Shanbhag , C. I. Sathish , K. Ramadass , A. Vinu , Emergent. Mater. 2019, 2, 337;

[26] D. Li , J. Yang , Y. Zhao , H. Yuan , Y. Chen , J. Clean. Prod. 2022, 130283.

[27] J. Li , C. Shi , A. Bao , J. Environ. Chem. Eng. 2021, 9, 105250.

[28] X. Zhang , I. Elsayed , R. O. Nayanathara , X. Song , R. Shmulsky , E. B. Hassan , J. Environ. Chem. Eng. 2022, 10, 107460.

[29] a) G. Singh , J. Lee , R. Bahadur , A. Karakoti , J. Yi , A. Vinu , Chem. Eng. J. 2022, 433, 134464;

[30] J. J. Manyà , B. González , M. Azuara , G. Arner , Chem. Eng. J. 2018, 345, 631.

[31] a) X. Zhang , S. Zhang , H. Yang , Y. Feng , Y. Chen , X. Wang , H. Chen , Chem. Eng. J. 2014, 257, 20;

[32] a) C. Zhang , W. Song , Q. Ma , L. Xie , X. Zhang , H. Guo , Energ. Fuel 2016, 30, 4181;

[33] X. Yuan , M. Suvarna , S. Low , P. D. Dissanayake , K. B. Lee , J. Li , X. Wang , Y. S. Ok , Environ. Sci. Technol. 2021, 55, 11925. 34291911 10.1021/acs.est.1c01849

[34] a) Z. Zhang , J. A. Schott , M. Liu , H. Chen , X. Lu , B. G. Sumpter , J. Fu , S. Dai , Angew. Chem. 2019, 131, 265; 10.1002/anie.201812363 30511416

[35] a) X. Shao , Z. Feng , R. Xue , C. Ma , W. Wang , X. Peng , D. Cao , AIChE J. 2011, 57, 3042;

[36] V. K. Singh , E. A. Kumar , Appl. Therm. Eng. 2016, 97, 77.

[37] M. A. A. Ahamed , M. S. A. Perera , S. K. Matthai , P. G. Ranjith , L. Dong‐yin , J. Pet. Sci. Eng. 2019, 180, 901.

[38] X. Li , Y. Liu , J. Hao , W. Wang , Mater. 2018, 11, 1782.

[39] N. A. Sagar , A. Khar , Vikas , A. Tarafdar , S. Pareek , J. Food Qual. 2021, 2021, 7178618.

[40] A. M. Shoaib , R. A. El‐Adly , M. H. M. Hassanean , A. Youssry , A. A. Bhran , Egypt. J. Pet. 2018, 27, 1305.

[41] N. Dedovic , S. Igic , T. Janic , S. Matic‐Kekic , O. Ponjican , M. Tomic , L. Savin , Energies 2012, 5, 1470.

[42] S. C. Dey , M. Al‐Amin , T. U. Rashid , M. Z. Sultan , M. Ashaduzzaman , M. Sarker , S. M. Shamsuddin , Int. J. Latest Res. Eng. Technol. 2016, 2, 52.

[43] V. Placet , A. Day , J. Beaugrand , J. Mater. Sci. 2017, 52, 5759.

[44] N. Arena , J. Lee , R. Clift , J. Clean. Prod. 2016, 125, 68.

[45] E. Alvarado‐Gómez , J. I. Tapia , A. Encinas , Sustain. Mater. Techno. 2021, 28, e00273.

[46] R. Kumari , J. Mohanta , B. Dey , S. Dey , Sep. Sci. Technol. 2020, 55, 3047.

[47] G. Singh , K. S. Lakhi , K. Ramadass , S. Kim , D. Stockdale , A. Vinu , Micropor. Mesopor. Mater. 2018, 271, 23.

[48] a) R. Chakraborty , K. Vilya , M. Pradhan , A. K. Nayak , J. Mater. Chem. A 2022, 10, 6965;

[49] a) H. B. M. Emrooz , M. Maleki , A. Rahmani , J. Taiwan Inst. Chem. Eng. 2018, 91, 281;

[50] a) M. Vafaeinia , M. S. Khosrowshahi , H. Mashhadimoslem , H. B. M. Emrooz , A. Ghaemi , RSC Adv. 2022, 12, 546; 10.1039/d1ra08407k PMC8694228 35424508

[51] M. Olivares‐Marín , S. Garcia , C. Pevida , M. Wong , M. Maroto‐Valer , J. Environ. Manage. 2011, 92, 2810. 21763061 10.1016/j.jenvman.2011.06.031

[52] T. Liang , C. Chen , X. Li , J. Zhang , Langmuir 2016, 32, 8042. 27455183 10.1021/acs.langmuir.6b01953

[53] S. Balou , S. E. Babak , A. Priye , ACS Appl. Mater. Interfaces 2020, 12, 42711. 32845602 10.1021/acsami.0c10218

[54] a) X.‐q. Zhang , W.‐c. Li , A.‐h. Lu , New Carbon Mater. 2015, 30, 481;

[55] C. Li , Y. Wang , N. Xiao , H. Li , Y. Ji , Z. Guo , C. Liu , J. Qiu , Carbon 2019, 151, 46.

[56] Z. Zhang , Z. P. Cano , D. Luo , H. Dou , A. Yu , Z. Chen , J. Mater. Chem. A 2019, 7, 20985.

[57] a) Y. Wang , X. Hu , J. Hao , R. Ma , Q. Guo , H. Gao , H. Bai , Ind. Eng. Chem. Res. 2019, 58, 13390;

[58] E. Jang , S. W. Choi , K. B. Lee , Fuel 2019, 248, 85.

[59] G. Singh , K. S. Lakhi , S. Sil , S. V. Bhosale , I. Kim , K. Albahily , A. Vinu , Carbon 2019, 148, 164.

[60] L. Rao , R. Ma , S. Liu , L. Wang , Z. Wu , J. Yang , X. Hu , Chem. Eng. J. 2019, 362, 794.

[61] G. Singh , J. Lee , A. Karakoti , R. Bahadur , J. Yi , D. Zhao , K. AlBahily , A. Vinu , Chem. Soc. Rev. 2020, 49, 4360. 32458938 10.1039/d0cs00075b

[62] a) J. Sherwood , Bioresour. Technol. 2020, 300, 122755; 31956060 10.1016/j.biortech.2020.122755

[63] R. Wu , A. Bao , J. CO2 Util. 2023, 68, 102377.

[64] a) K. Yogalakshmi , P. Sivashanmugam , S. Kavitha , Y. Kannah , S. Varjani , S. AdishKumar , G. Kumar , Chemosphere 2022, 286, 131824; 34388872 10.1016/j.chemosphere.2021.131824

[65] S. V. Vassilev , D. Baxter , L. K. Andersen , C. G. Vassileva , Fuel 2010, 89, 913.

[66] Z. Hameed , M. Aslam , Z. Khan , K. Maqsood , A. Atabani , M. Ghauri , M. S. Khurram , M. Rehan , A.‐S. Nizami , Renewable Sustainable Energy Rev. 2021, 136, 110375.

[67] E. Kontturi , S. Spirk , Front. Chem. 2019, 7, 488. 31380342 10.3389/fchem.2019.00488 PMC6652239

[68] a) L.‐Z. Huang , M.‐G. Ma , X.‐X. Ji , S.‐E. Choi , C. Si , Front. Bioeng. Biotechnol. 2021, 9, 440; 10.3389/fbioe.2021.690773 PMC8258147 34239863

[69] P. González‐García , Renewable Sustainable Energy Rev. 2018, 82, 1393.

[70] H. Yang , R. Yan , H. Chen , D. H. Lee , C. Zheng , Fuel 2007, 86, 1781.

[71] A. Demirbas , Energy Sources, Part A 2008, 30, 1114.

[72] a) X. Y. Chen , C. Chen , Z. J. Zhang , D. H. Xie , J. Mater. Chem. A 2013, 1, 7379;

[73] a) M. Olivares‐Marín , M. M. Maroto‐Valer , Greenh. Gases: Sci. Technol. 2012, 2, 20;

[74] a) G. Singh , A. M. Ruban , X. Geng , A. Vinu , Chem. Eng. J. 2022, 139045;

[75] a) J. Pastor‐Villegas , J. Pastor‐Valle , J. M. Rodríguez , M. G. García , J. Anal. Appl. Pyrolysis 2006, 76, 103;

[76] G. Singh , I. Y. Kim , K. S. Lakhi , S. Joseph , P. Srivastava , R. Naidu , A. Vinu , J. Mater. Chem. A 2017, 5, 21196.

[77] a) J. Xiao , Y. Wang , T. C. Zhang , S. Yuan , Appl. Surf. Sci. 2021, 562, 150128;

[78] L. Wang , Q. Zhu , J. Zhao , Y. Guan , J. Liu , Z. An , B. Xu , Micropor. Mesopor. Mater. 2019, 279, 439.

[79] a) J. Yang , J. Xie , X. Zhou , Y. Zou , J. Tang , S. Wang , F. Chen , L. Wang , J. Phys. Chem. C 2014, 118, 1800;

[80] H. Shang , Y. Lu , F. Zhao , C. Chao , B. Zhang , H. Zhang , RSC Adv. 2015, 5, 75728.

[81] a) X.‐L. Zhou , H. Zhang , L.‐M. Shao , F. Lü , P.‐J. He , Waste Biomass Valorization. 2021, 12, 1699;

[82] A. E. Ogungbenro , D. V. Quang , K. A. Al‐Ali , L. F. Vega , M. R. Abu‐Zahra , J. Environ. Chem. Eng. 2018, 6, 4245.

[83] a) X. Ma , B. Liu , M. Che , Q. Wu , R. Chen , C. Su , X. Xu , Z. Zeng , L. Li , Sep. Purif. Technol. 2021, 269, 118690;

[84] F. Rodriguez‐Reinoso , M. Molina‐Sabio , Carbon 1992, 30, 1111.

[85] a) B. Campbell , R. Ionescu , Z. Favors , C. S. Ozkan , M. Ozkan , Sci. Rep. 2015, 5, 1; 10.1038/srep14575 PMC4586494 26415917

[86] a) J. Yin , W. Zhang , N. A. Alhebshi , N. Salah , H. N. Alshareef , Small Methods 2020, 4, 1900853;

[87] G. Nazir , A. Rehman , S.‐J. Park , J. Environ. Manage. 2021, 299, 113661. 34481373 10.1016/j.jenvman.2021.113661

[88] Y. Sun , H. Zhang , N. Yuan , Y. Ge , Y. Dai , Z. Yang , L. Lu , J. Hazard. Mater. 2021, 413, 125282. 33582468 10.1016/j.jhazmat.2021.125282

[89] J. Lee , J. Kim , T. Hyeon , Adv. Mater. 2006, 18, 2073.

[90] E. Hao , W. Liu , S. Liu , Y. Zhang , H. Wang , S. Chen , F. Cheng , S. Zhao , H. Yang , J. Mater. Chem. A 2017, 5, 2204.

[91] C. Quan , Y. Zhou , C. Wu , G. Xu , D. Feng , Y. Zhang , N. Gao , J. Anal. Appl. Pyrolysis 2023, 105874.

[92] X. Xing , W. Jiang , S. Li , X. Zhang , W. Wang , Waste Manag. 2019, 89, 64. 31079760 10.1016/j.wasman.2019.04.002

[93] a) J. Wang , S. Chen , J.‐y. Xu , L.‐c. Liu , J.‐c. Zhou , J.‐j. Cai , New Carbon Mater. 2021, 36, 1081;

[94] G. Singh , K. S. Lakhi , K. Ramadass , C. Sathish , A. Vinu , ACS Sustainable Chem. Eng. 2019, 7, 7412.

[95] J. Deng , T. Xiong , F. Xu , M. Li , C. Han , Y. Gong , H. Wang , Y. Wang , Green Chem. 2015, 17, 4053.

[96] M. Xia , W. Chen , J. Wu , Y. Chen , H. Yang , X. Chen , D. Zhu , H. Chen , Fuel 2021, 291, 120185.

[97] a) C. Zhang , X. Ren , L. Kou , X. Zhang , R. Wang , L. Xie , C. Fan , J. Environ. Chem. Eng. 2021, 9, 106605;

[98] Z. Geng , Q. Xiao , H. Lv , B. Li , H. Wu , Y. Lu , C. Zhang , Sci. Rep. 2016, 6, 1. 27488268 10.1038/srep30049 PMC4973261

[99] a) M. G. Singh , K. S. Lakhi , D. H. Park , P. Srivastava , R. Naidu , A. Vinu , ChemNanoMat 2018, 4, 281;

[100] a) Z. Xie , X. Shang , J. Yan , T. Hussain , P. Nie , J. Liu , Electrochim. Acta 2018, 290, 666;

[101] a) C.‐H. Ooi , W.‐K. Cheah , Y.‐L. Sim , S.‐Y. Pung , F.‐Y. Yeoh , J. Environ. Manage. 2017, 197, 199; 28384613 10.1016/j.jenvman.2017.03.083

[102] Y. Boyjoo , Y. Cheng , H. Zhong , H. Tian , J. Pan , V. K. Pareek , J.‐F. Lamonier , M. Jaroniec , J. Liu , Carbon 2017, 116, 490.

[103] S.‐H. Liu , Y.‐Y. Huang , J. Clean. Prod. 2018, 175, 354.

[104] M. Sevilla , A. B. Fuertes , Environ. Sci. 2011, 4, 1765.

[105] L. Cibien , M. Parot , P. N. Fotsing , P. Gaveau , E. D. Woumfo , J. Vieillard , A. Napoli , N. Brun , Green Chem. 2020, 22, 5423.

[106] a) K. S. Song , S. N. Talapaneni , T. Ashirov , A. Coskun , ACS Appl. Mater. Interfaces 2021, 13, 26102; 34038084 10.1021/acsami.1c06326

[107] P. Wang , G. Zhang , W. Chen , Q. Chen , H. Jiao , L. Liu , X. Wang , X. Deng , ACS Omega 2020, 5, 23460. 32954199 10.1021/acsomega.0c03497 PMC7496027

[108] a) P. Strubel , H. Althues , S. Kaskel , Carbon 2016, 107, 705;

[109] S. Yu , N. Sun , L. Hu , L. Wang , Q. Zhu , Y. Guan , B. Xu , J. Power Sources 2018, 405, 132.

[110] A. Wang , K. Sun , R. Xu , Y. Sun , J. Jiang , J. Clean. Prod. 2021, 283, 125385.

[111] K. Sun , C.‐y. Leng , J.‐c. Jiang , Q. Bu , G.‐f. Lin , X.‐c. Lu , G.‐z. Zhu , New Carbon Mater. 2017, 32, 451.

[112] C. Xia , S. Q. Shi , Green Chem. 2016, 18, 2063.

[113] H. B. M. Emrooz , M. S. H. Naghavi , S. Mohammadi , S. M. Mousavi‐Khoshdel , J. Energy Storage 2022, 56, 105989.

[114] H. M. Mobin Safarzadeh Khosrowshahi , H. B. M. Emrooz , A. Ghaemi , M. S. Hosseini , Diam. Relat. Mater. 2022, 127, 109204.

[115] H. Mashhadimoslem , M. Safarzadeh , A. Ghaemi , H. B. M. Emrooz , M. Barzegar , RSC Adv. 2021, 11, 36125. 35492770 10.1039/d1ra06781h PMC9043437

[116] H. Xu , H. Li , L. Xie , D. Zhao , B. Kong , Adv. Mater. Interfaces 2022, 9, 2101998.

[117] Y.‐y. Feng , Y.‐q. Chen , Z. Wang , J. Wei , New Carbon Mater. 2022, 37, 196.

[118] a) S. Chen , W. Xing , J. Duan , X. Hu , S. Z. Qiao , J. Mater. Chem. A 2013, 1, 2941;

[119] B. Xue , J. Xu , R. Xiao , ACS Sustainable Chem. Eng. 2021, 9, 15925.

[120] F. Xu , Y. Chen , M. Tang , H. Wang , J. Deng , Y. Wang , ACS Sustainable Chem. Eng. 2016, 4, 4473.

[121] D. Xin‐Hui , C. Srinivasakannan , P. Jin‐Hui , Z. Li‐Bo , Z. Zheng‐Yong , Biomass Bioenergy 2011, 35, 3920.

[122] J. Guo , A. C. Lua , Carbon 2000, 38, 1985.

[123] W. Ao , J. Fu , X. Mao , Q. Kang , C. Ran , Y. Liu , H. Zhang , Z. Gao , J. Li , G. Liu , Renewable Sustainable Energy Rev. 2018, 92, 958.

[124] a) K. Yang , J. Peng , C. Srinivasakannan , L. Zhang , H. Xia , X. Duan , Bioresour. Technol. 2010, 101, 6163; 20303745 10.1016/j.biortech.2010.03.001

[125] M. J. Ahmed , J. Environ. Chem. Eng. 2016, 4, 89.

[126] F. Zhang , T. Zhou , Y. Liu , J. Leng , Sci. Rep. 2015, 5, 1. 10.1038/srep11152 PMC4459203 26053586

[127] a) N. A. Kumar , H. Nolan , N. McEvoy , E. Rezvani , R. L. Doyle , M. E. Lyons , G. S. Duesberg , J. Mater. Chem. A 2013, 1, 4431;

[128] G.‐Q. Wu , X. Zhang , H. Hui , J. Yan , Q.‐S. Zhang , J.‐L. Wan , Y. Dai , Chem. Eng. J. 2012, 185, 201.

[129] B. Zhang , P. Xu , Y. Qiu , Q. Yu , J. Ma , H. Wu , G. Luo , M. Xu , H. Yao , Chem. Eng. J. 2015, 263, 1.

[130] D. Wu , Y. Yang , J. Liu , Y. Zheng , Energ. Fuel 2020, 34, 6077.

[131] S. Iijima , Nature 1991, 354, 56.

[132] F. Jiang , Y. Yao , B. Natarajan , C. Yang , T. Gao , H. Xie , Y. Wang , L. Xu , Y. Chen , J. Gilman , L. Cui , L. Hu , Carbon 2019, 144, 241.

[133] Y. Li , Y. Chen , A. Nie , A. Lu , R. J. Jacob , T. Gao , J. Song , J. Dai , J. Wan , G. Pastel , M. R. Zachariah , R. S. Yassar , L. Hu , Adv. Energy Mater. 2017, 7, 1601783.

[134] a) H. M. Emrooz , A. Rahmani , Mater. Sci. Semicond Process 2017, 72, 15;

[135] X. Ma , N. Xiao , J. Xiao , X. Song , H. Guo , Y. Wang , S. Zhao , Y. Zhong , J. Qiu , Carbon 2021, 179, 33.

[136] E. Köseoğlu , C. Akmil‐Başar , Adv. Powder Technol. 2015, 26, 811.

[137] K. S. Ukanwa , K. Patchigolla , R. Sakrabani , E. Anthony , S. Mandavgane , Sustainability 2019, 11, 6204.

[138] M. Vinayagam , R. S. Babu , A. Sivasamy , A. L. F. de Barros , Biomass Bioenergy 2020, 143, 105838.

[139] a) H. Huo , Y. Ma , X. Wang , ChemistrySelect 2021, 6, 1814;

[140] D. M. Alonso , S. G. Wettstein , J. A. Dumesic , Chem. Soc. Rev. 2012, 41, 8075. 22872312 10.1039/c2cs35188a

[141] G. S. Szymański , Z. Karpiński , S. Biniak , A. Świa̧tkowski , Carbon 2002, 40, 2627.

[142] a) J. Niu , R. Shao , M. Liu , J. Liang , Z. Zhang , M. Dou , Y. Huang , F. Wang , Energy Storage Mater. 2018, 12, 145;

[143] M. S. Khosrowshahi , M. A. Abdol , H. Mashhadimoslem , E. Khakpour , H. B. M. Emrooz , S. Sadeghzadeh , A. Ghaemi , Sci. Rep. 2022, 12, 8917. 35618757 10.1038/s41598-022-12596-5 PMC9135713

[144] Y. Liu , X. Liu , W. Dong , L. Zhang , Q. Kong , W. Wang , Sci. Rep. 2017, 7, 12437. 28963547 10.1038/s41598-017-12805-6 PMC5622173

[145] a) Z. Bacsik , N. Ahlsten , A. Ziadi , G. Zhao , A. E. Garcia‐Bennett , B. Martín‐Matute , N. Hedin , Langmuir 2011, 27, 11118; 21774480 10.1021/la202033p PMC3164231

[146] a) M. Badihehaghdam , S. M. Mousavi Khoie , F. Khast , M. Safarzadeh Khosrowshahi , Met. Mater. Int. 2021, 1;

[147] D. Gang , Z. U. Ahmad , Q. Lian , L. Yao , M. E. Zappi , Chem. Eng. J. 2021, 403, 126286.

[148] C. Goel , H. Bhunia , P. K. Bajpai , J. Environ. Chem. Eng. 2016, 4, 346.

[149] H. M. Coromina , D. A. Walsh , R. Mokaya , J. Mater. Chem. A 2016, 4, 280.

[150] M. Cox , R. Mokaya , Sustain. Energ. Fuels 2017, 1, 1414.

[151] a) R. A. Fiuza‐Jr , R. C. Andrade , H. M. C. Andrade , J. Environ. Chem. Eng. 2016, 4, 4229;

[152] a) M. A. A. Al‐Maadeed , D. Ponnamma , M. A. Carignano , Polymer Science and Innovative Applications: Materials, Techniques, and Future Developments, Elsevier, 2020;

[153] P. Ruz , S. Banerjee , M. Pandey , V. Sudarsan , P. Sastry , R. Kshirsagar , Solid State Sci. 2016, 62, 105.

[154] A. Wang , Z. Zheng , R. Li , D. Hu , Y. Lu , H. Luo , K. Yan , Green Energy Environ. 2019, 4, 414.

[155] a) R. K. Selvan , P. Zhu , C. Yan , J. Zhu , M. Dirican , A. Shanmugavani , Y. S. Lee , X. Zhang , J. Colloid Interface Sci. 2018, 513, 231; 29153717 10.1016/j.jcis.2017.11.016

[156] L. G. Cançado , A. Jorio , E. M. Ferreira , F. Stavale , C. A. Achete , R. B. Capaz , M. V. d. O. Moutinho , A. Lombardo , T. Kulmala , A. C. Ferrari , Nano Lett. 2011, 11, 3190. 21696186 10.1021/nl201432g

[157] F. E. C. Othman , N. Yusof , S. Samitsu , N. Abdullah , M. F. Hamid , K. Nagai , M. N. Z. Abidin , M. A. Azali , A. F. Ismail , J. Jaafar , J. CO2 Util. 2021, 45, 101434.

[158] Z. Liu , W. Li , P. Z. Moghadam , S. Li , Sustain. Energ. Fuels 2021, 5, 1075.

[159] S. Fu , Q. Fang , A. Li , Z. Li , J. Han , X. Dang , W. Han , Energy Sci. Eng. 2021, 9, 80.

[160] M. Rajasekaran , K. G. Ayappa , Phys. Chem. Chem. Phys. 2022, 24, 14909. 35674363 10.1039/d1cp03962h

[161] B. Abebe , H. A. Murthy , E. Amare , J. Encapsulation Adsorpt. Sci. 2018, 8, 225.

[162] E. M. Mistar , T. Alfatah , M. D. Supardan , J. Mater Res. Technol. 2020, 9, 6278.

[163] P. Ozpinar , C. Dogan , H. Demiral , U. Morali , S. Erol , C. Samdan , D. Yildiz , I. Demiral , Renew. Energ. 2022, 189, 535.

[164] a) C. Jiang , G. A. Yakaboylu , T. Yumak , J. W. Zondlo , E. M. Sabolsky , J. Wang , Renew. Energ. 2020, 155, 38;

[165] a) S. Allen , G. Mckay , J. F. Porter , J. Colloid Interface Sci. 2004, 280, 322; 15533404 10.1016/j.jcis.2004.08.078

[166] a) M. Ghiaci , A. Abbaspur , R. Kia , F. Seyedeyn‐Azad , Sep. Purif. Technol. 2004, 40, 217;

[167] a) E. Bulut , M. Özacar , İ. A. Şengil , Micropor. Mesopor. Mater. 2008, 115, 234;

[168] a) A. A. Alhwaige , Novel Biobased Chitosan/Polybenzoxazine Cross‐Linked Polymers and Advanced Carbon Aerogels for CO2 Adsorption, Case Western Reserve University, 2014;

[169] G. Yang , S. Song , J. Li , Z. Tang , J. Ye , J. Yang , J. Mater. Sci. Technol. 2019, 35, 875.

[170] S. He , G. Chen , H. Xiao , G. Shi , C. Ruan , Y. Ma , H. Dai , B. Yuan , X. Chen , X. Yang , J. Colloid Interface Sci. 2021, 582, 90. 32814226 10.1016/j.jcis.2020.08.021

[171] M.‐J. Kim , S. W. Choi , H. Kim , S. Mun , K. B. Lee , Chem. Eng. J. 2020, 397, 125404.

[172] G. K. Parshetti , S. Chowdhury , R. Balasubramanian , Fuel 2015, 148, 246.

[173] L. Shao , Y. Sang , N. Liu , J. Liu , P. Zhan , J. Huang , J. Chen , ACS Omega 2020, 5, 17450. 32715230 10.1021/acsomega.0c01918 PMC7377076

[174] K. Y. Foo , B. H. Hameed , Chem. Eng. J. 2010, 156, 2.

[175] M. Dubinin , V. Astakhov , Bull. Acad. Sci. USSR, Div. Chem. Sci. (Engl. Transl.) 1971, 20, 8.

[176] M. Dubinin , Dokl. Akad. Nauk SSSR 1947, 327.

[177] H. Freundlich , J. Phys. Chem. 1906, 57, 1100.

[178] G. Halsey , J. Chem. Phys. 1948, 16, 931.

[179] N. Ayawei , A. N. Ebelegi , D. Wankasi , J. Chem. 2017, 2017.

[180] D. Jovanović , Kolloid‐Zeitschrift und Zeitschrift für Polymere 1969, 235, 1214.

[181] I. Langmuir , J. Am. Chem. Soc. 1916, 38, 2221.

[182] W. McMillan , E. Teller , J. Phys. Chem. 1951, 55, 17. 10.1021/j150484a003 14814627

[183] K. Vijayaraghavan , T. Padmesh , K. Palanivelu , M. Velan , J. Hazard. Mater. 2006, 133, 304. 16297540 10.1016/j.jhazmat.2005.10.016

[184] R. Sips , J. Chem. Phys. 1948, 16, 490.

[185] M. Tempkin , V. Pyzhev , Acta Phys. Chim. USSR 1940, 12, 327.

[186] J. Tóth , Adv. Colloid Interface Sci. 1995, 55, 1.

[187] a) J. Liu , H. Li , H. Zhang , Q. Liu , R. Li , B. Li , J. Wang , J. Solid State Chem. 2018, 257, 64;

[188] a) J. Tey , M. Careem , M. Yarmo , A. Arof , Ionics 2016, 22, 1209;

[189] B. Sun , Y. Yuan , H. Li , X. Li , C. Zhang , F. Guo , X. Liu , K. Wang , X. Zhao , Chem. Eng. J. 2019, 371, 55.

[190] C. Wang , X. Wang , H. Lu , H. Li , X. Zhao , Carbon 2018, 140, 139.

[191] A. Gopalakrishnan , T. D. Raju , S. Badhulika , Carbon 2020, 168, 209.

[192] a) H. Wang , H. Wang , G. Liu , Q. Yan , Sci. Total Environ. 2021, 771, 145424; 33548725 10.1016/j.scitotenv.2021.145424

[193] S. Wang , Y.‐R. Lee , Y. Won , H. Kim , S.‐E. Jeong , B. W. Hwang , A. R. Cho , J.‐Y. Kim , Y. C. Park , H. Nam , Chem. Eng. J. 2022, 135378.

[194] a) G. Singh , I. S. Ismail , C. Bilen , D. Shanbhag , C. Sathish , K. Ramadass , A. Vinu , Appl. Energy 2019, 255, 113831;

[195] a) A. A. Abd , S. Z. Naji , A. S. Hashim , M. R. Othman , J. Environ. Chem. Eng. 2020, 8, 104142;

[196] X. Xu , Z. Xu , B. Gao , L. Zhao , Y. Zheng , J. Huang , D. C. Tsang , Y. S. Ok , X. Cao , Chem. Eng. J. 2020, 384, 123289.

[197] R. S. Liu , X. D. Shi , C. T. Wang , Y. Z. Gao , S. Xu , G. P. Hao , S. Chen , A. H. Lu , ChemSusChem 2021, 14, 1428. 33403787 10.1002/cssc.202002677

[198] a) Y. Wang , J. Wang , C. Ma , W. Qiao , L. Ling , J. Colloid Interface Sci. 2019, 534, 72; 30216834 10.1016/j.jcis.2018.08.063

[199] C. Pevida , F. Rubiera , Energies 2023, 16, 667.

[200] H. Marsh , B. Rand , J. Colloid Interface Sci. 1970, 33, 101.

[201] G. Nazir , A. Rehman , S.‐J. Park , J. CO2 Util. 2021, 51, 101641.

[202] L. Li , J. Yang , J. Li , Y. Chen , J. Li , Micropor. Mesopor. Mater. 2014, 198, 236.

[203] L. Lei , Y. Cheng , C. Chen , M. Kosari , Z. Jiang , C. He , J. Colloid Interface Sci. 2022, 612, 132. 34992014 10.1016/j.jcis.2021.12.163

[204] a) H. Zhou , C. Rayer , A. R. Antonangelo , N. Hawkins , M. Carta , ACS Appl. Mater. Interfaces 2022, 18, 20997; 10.1021/acsami.2c02604 PMC9100501 35471026

[205] S. G. Subraveti , K. N. Pai , A. K. Rajagopalan , N. S. Wilkins , A. Rajendran , A. Jayaraman , G. Alptekin , Appl. Energy 2019, 254, 113624.

[206] Y. Lian , S. Deng , S. Li , Z. Guo , L. Zhao , X. Yuan , Int. J. Greenh. Gas Control. 2019, 85, 187.

[207] D. Xu , P. Xiao , J. Zhang , G. Li , G. Xiao , P. A. Webley , Y. Zhai , Chem. Eng. J. 2013, 230, 64.

[208] C. A. Grande , A. E. Rodrigues , Int. J. Greenh. Gas Control. 2008, 2, 194.

[209] C. Shen , Z. Liu , P. Li , J. Yu , Ind. Eng. Chem. Res. 2012, 51, 5011.

[210] A. K. Rajagopalan , A. M. Avila , A. Rajendran , Int. J. Greenh. Gas Control. 2016, 46, 76.

[211] a) J. Srenscek‐Nazzal , U. Narkiewicz , A. W. Morawski , R. J. Wróbel , B. Michalkiewicz , J. Chem. Eng. Data 2015, 60, 3148;

[212] F. Raganati , F. Miccio , P. Ammendola , Energ. Fuel 2021, 35, 12845.

[213] B. Chen , Z. Yang , G. Ma , D. Kong , W. Xiong , J. Wang , Y. Zhu , Y. Xia , Micropor. Mesopor. Mater. 2018, 257, 1.

[214] M. B. Ahmed , M. A. H. Johir , J. L. Zhou , H. H. Ngo , L. D. Nghiem , C. Richardson , M. A. Moni , M. R. Bryant , J. Clean. Prod. 2019, 225, 405.

[215] N. A. Zubbri , A. R. Mohamed , P. Lahijani , M. Mohammadi , J. Environ. Chem. Eng. 2021, 9, 105074.

[216] F. Shen , Y. Wang , L. Li , K. Zhang , R. L. Smith , X. Qi , Chem. Eng. Commun. 2018, 205, 423.

[217] X. Ma , Y. Yang , Q. Wu , B. Liu , D. Li , R. Chen , C. Wang , H. Li , Z. Zeng , L. Li , Fuel 2020, 282, 118727.

[218] F. Yang , J. Wang , L. Liu , P. Zhang , W. Yu , Q. Deng , Z. Zeng , S. Deng , ACS Sustainable Chem. Eng. 2018, 6, 15550.

[219] A. Alabadi , S. Razzaque , Y. Yang , S. Chen , B. Tan , Chem. Eng. J. 2015, 281, 606.

[220] Q. Li , T. Lu , L. Wang , R. Pang , J. Shao , L. Liu , X. Hu , Sep. Purif. Technol. 2021, 275, 119204.

[221] L. Yue , L. Rao , L. Wang , L. An , C. Hou , C. Ma , H. DaCosta , X. Hu , Energ. Fuel 2018, 32, 6955.

[222] Z. Tian , Y. Qiu , J. Zhou , X. Zhao , J. Cai , Mater. Lett. 2016, 180, 162.

[223] H. Cui , J. Xu , J. Shi , N. Yan , Y. Liu , Energy 2019, 187, 115936.

[224] Y. Zhang , Z. Wei , X. Liu , F. Liu , Z. Yan , S. Zhou , J. Wang , S. Deng , RSC Adv. 2022, 12, 8592. 35424789 10.1039/d2ra00139j PMC8985111

[225] S. Ding , Y. Liu , Fuel 2020, 260, 116382.

[226] J. Serafin , U. Narkiewicz , A. W. Morawski , R. J. Wróbel , B. Michalkiewicz , J. CO2 Util. 2017, 18, 73.

[227] N. A. Rashidi , S. Yusup , J. Clean. Prod. 2017, 168, 474.

[228] T. Chen , S. Deng , B. Wang , J. Huang , Y. Wang , G. Yu , RSC Adv. 2015, 5, 48323.

[229] B. Chang , W. Shi , H. Yin , S. Zhang , B. Yang , Chem. Eng. J. 2019, 358, 1507.

[230] a) S. Jung , J.‐R. Lee , Y. Won , D.‐H. Lee , Y. C. Park , Y.‐S. Bae , H. Kim , J. CO2 Util. 2021, 51, 101659;

[231] S. Liu , J. Ma , S. Sang , T. Wang , Y. Du , H. Fang , Fuel 2018, 223, 32.

[232] M. Vorokhta , J. Morávková , M. Dopita , A. Zhigunov , M. Šlouf , R. Pilař , P. Sazama , Adsorption 2021, 27, 1221.

[233] E. Maruccia , M. Lourenço , T. Priamushko , M. Bartoli , S. Bocchini , F. Pirri , G. Saracco , F. Kleitz , C. Gerbaldi , Mater. Today Sustain. 2022, 17, 100089.

[234] E. García‐Díez , A. Castro‐Muñiz , J. I. Paredes , M. M. Maroto‐Valer , F. Suárez‐García , S. García , Micropor. Mesopor. Mater. 2021, 318, 110986.

[235] a) M. Sevilla , A. B. Fuertes , J. Colloid Interface Sci. 2012, 366, 147; 21999954 10.1016/j.jcis.2011.09.038

[236] J. Serafin , B. Dziejarski , O. F. C. Junior , J. Sreńscek‐Nazzal , Carbon 2023, 201, 633.

[237] H. Wei , S. Deng , B. Hu , Z. Chen , B. Wang , J. Huang , G. Yu , ChemSusChem 2012, 5, 2354. 23132775 10.1002/cssc.201200570

[238] A. Vinu , Adv. Funct. Mater. 2008, 18, 816.

[239] a) D. Wu , J. Liu , Y. Yang , Y. Zheng , Ind. Eng. Chem. Res. 2020, 59, 14055;

[240] Z. Zhao , C. Ma , F. Chen , G. Xu , R. Pang , X. Qian , J. Shao , X. Hu , Biomass Bioenergy 2021, 145, 105969.

[241] a) J. Zhang , R. Singh , P. A. Webley , Micropor. Mesopor. Mater. 2008, 111, 478;

[242] W. Xing , C. Liu , Z. Zhou , L. Zhang , J. Zhou , S. Zhuo , Z. Yan , H. Gao , G. Wang , S. Z. Qiao , Environ. Sci. 2012, 5, 7323.

[243] M. Zhu , W. Cai , F. Verpoort , J. Zhou , Chem. Eng. Res. Des. 2019, 146, 130.

[244] M. Sevilla , J. B. Parra , A. B. Fuertes , ACS Appl. Mater. Interfaces 2013, 5, 6360. 23789916 10.1021/am401423b

[245] X. Song , L. a. Wang , J. Gong , X. Zhan , Y. Zeng , Langmuir 2020, 36, 3862. 32109066 10.1021/acs.langmuir.9b03475

[246] M. Montes‐Morán , D. Suárez , J. Menéndez , E. Fuente , Carbon 2004, 42, 1219.

[247] G. Yin , Z. Liu , Q. Liu , W. Wu , Chem. Eng. J. 2013, 230, 133.

[248] a) S. Hao , J. Wen , X. Yu , W. Chu , Appl. Surf. Sci. 2013, 264, 433;

[249] a) R. R. Da Silva , J. Torres , Y. Kopelevich , Phys. Rev. Lett. 2001, 87, 147001; 11580670 10.1103/PhysRevLett.87.147001

[250] B. Petrovic , M. Gorbounov , S. M. Soltani , Carbon Capture Sci. Technol. 2022, 100045.

[251] H. Seema , K. C. Kemp , N. H. Le , S.‐W. Park , V. Chandra , J. W. Lee , K. S. Kim , Carbon 2014, 66, 320.

[252] P. Yang , L. Rao , W. Zhu , L. Wang , R. Ma , F. Chen , G. Lin , X. Hu , Ind. Eng. Chem. Res. 2020, 59, 6194.

[253] a) M. Lee , S. Hong , D. Kim , E. Kim , K. Lim , J. C. Jung , H. Richter , J.‐H. Moon , N. Choi , J. Nam , ACS Appl. Mater. Interfaces 2019, 11, 3946; 30614677 10.1021/acsami.8b18854

[254] S. V. Vassilev , C. G. Vassileva , V. S. Vassilev , Fuel 2015, 158, 330.

[255] B. M. Matsagar , R.‐X. Yang , S. Dutta , Y. S. Ok , K. C.‐W. Wu , J. Mater. Chem. A 2021, 9, 3703.

[256] W. Chen , X. Wang , Z. Hashisho , M. Feizbakhshan , P. Shariaty , S. Niknaddaf , X. Zhou , Micropor. Mesopor. Mater. 2019, 280, 57.

[257] H. B. M. Emrooz , M. Maleki , M. Shokouhimehr , J. Taiwan Inst. Chem. Eng. 2019, 102, 99.

[258] a) L. Lyu , K.‐d. Seong , D. Ko , J. Choi , C. Lee , T. Hwang , Y. Cho , X. Jin , W. Zhang , H. Pang , Mater. Chem. Front. 2019, 3, 2543;

[259] M. Nowrouzi , H. Younesi , N. Bahramifar , Fuel 2018, 223, 99.

[260] Q. Pu , Y. Wang , X. Wang , Z. Shao , S. Wen , J. Wang , P. Ning , S. Lu , L. Huang , Q. Wang , J. CO2 Util. 2021, 54, 101756.

[261] M. Nisar , P. S. Thue , M. B. Maghous , J. Geshev , E. C. Lima , S. Einloft , RSC Adv. 2020, 10, 34595. 35514388 10.1039/d0ra06805e PMC9056794

[262] L.‐P. Guo , W.‐C. Li , B. Qiu , Z.‐X. Ren , J. Du , A.‐H. Lu , J. Mater. Chem. A 2019, 7, 5402.

[263] B. Zhu , K. Qiu , C. Shang , Z. Guo , J. Mater. Chem. A 2015, 3, 5212.

[264] K. Malini , D. Selvakumar , N. Kumar , J. CO2 Util. 2023, 67, 102318.

[265] A.‐M. Pierre‐Louis , D. B. Hausner , N. Bhandari , W. Li , J. Kim , J. D. Kubicki , D. Strongin , J. Colloid Interface Sci. 2013, 400, 1. 23561821 10.1016/j.jcis.2013.01.047

[266] A. E. Creamer , B. Gao , S. Wang , Chem. Eng. J. 2016, 283, 826.

[267] P. Lahijani , M. Mohammadi , A. R. Mohamed , J. CO2 Util. 2018, 26, 281.

[268] N. A. Zubbri , A. R. Mohamed , N. Kamiuchi , M. Mohammadi , Environ. Sci. Pollut. Res. 2020, 27, 11809. 10.1007/s11356-020-07734-3 31975005

[269] S. Hosseini , I. Bayesti , E. Marahel , F. E. Babadi , L. C. Abdullah , T. S. Choong , J. Taiwan Inst. Chem. Eng. 2015, 52, 109.

[270] a) F. Raganati , M. Alfe , V. Gargiulo , R. Chirone , P. Ammendola , Chem. Eng. Res. Des. 2018, 134, 540;

[271] a) J. M. Tascón , Novel Carbon Adsorbents, Elsevier, 2012;

[272] S. M. Mahurin , J. Górka , K. M. Nelson , R. T. Mayes , S. Dai , Carbon 2014, 67, 457.

[273] J. H. Lee , H. J. Lee , S. Y. Lim , B. G. Kim , J. W. Choi , J. Am. Chem. Soc. 2015, 137, 7210. 26000786 10.1021/jacs.5b03579

[274] a) E. Yang , H.‐Y. Li , F. Wang , H. Yang , J. Zhang , CrystEngComm 2013, 15, 658;

[275] D. M. D'Alessandro , B. Smit , J. R. Long , Angew. Chem., Int. Ed. 2010, 49, 6058. 10.1002/anie.201000431 20652916

[276] a) S. Ullah , M. A. Bustam , A. G. Al‐Sehemi , M. A. Assiri , F. A. A. Kareem , A. Mukhtar , M. Ayoub , G. Gonfa , Micropor. Mesopor. Mater. 2020, 296, 110002;

[277] Y. Zhang , L. Liu , P. Zhang , J. Wang , M. Xu , Q. Deng , Z. Zeng , S. Deng , Chem. Eng. J. 2019, 355, 309.

[278] Y.‐S. Bae , O. K. Farha , J. T. Hupp , R. Q. Snurr , J. Mater. Chem. 2009, 19, 2131.

[279] G. Nazir , A. Rehman , S.‐J. Park , J. CO2 Util. 2020, 42, 101326.

[280] a) J. Zhang , M. Clennell , D. Dewhurst , K. Liu , Fuel 2014, 122, 186;

[281] J. W. Lee , R. H. Nilson , J. A. Templeton , S. K. Griffiths , A. Kung , B. M. Wong , J. Chem. Theory Comput. 2012, 8, 2012. 23316120 10.1021/ct3001156 PMC3542913

[282] J. C. Palmer , J. D. Moore , T. J. Roussel , J. K. Brennan , K. E. Gubbins , Phys. Chem. Chem. Phys. 2011, 13, 3985. 21234499 10.1039/c0cp02281k

[283] K. V. Kumar , F. Rodríguez‐Reinoso , RSC Adv. 2012, 2, 9671.

[284] T. T. Trinh , T. J. Vlugt , M.‐B. Hägg , D. Bedeaux , S. Kjelstrup , Energy Procedia 2015, 64, 150.

[285] H. Aljaddani , S. M. Gatica , SN Appl. Sci. 2020, 2, 1.

[286] Y. Yang , A. K. Narayanan Nair , S. Sun , J. Phys. Chem. C 2020, 124, 16478. 10.1021/acs.jpcb.9b11840 31995385

[287] P.‐Y. Yang , S.‐P. Ju , S.‐M. Huang , Comp. Mater. Sci. 2018, 143, 43.

[288] J. Pikunic , R.‐M. Pellenq , K. Thomson , J.‐N. Rouzaud , P. Levitz , K. Gubbins , Elsevier, 2001, 132, 647.

[289] Y. Shi , J. Chem. Phys. 2008, 128, 234707. 18570519 10.1063/1.2943645

[290] E. Di Biase , L. Sarkisov , Carbon 2015, 94, 27.

[291] T. T. Trinh , K.‐Q. Tran , Q.‐V. Bach , D. Q. Trinh , Energy Procedia 2016, 86, 144.

[292] X. Ma , C. Su , B. Liu , Q. Wu , K. Zhou , Z. Zeng , L. Li , Sep. Purif. Technol. 2021, 259, 118065.

[293] X. Ma , L. Li , R. Chen , C. Wang , K. Zhou , H. Li , Fuel 2019, 236, 942.

[294] S. Wang , L. Lu , D. Wu , X. Lu , W. Cao , T. Yang , Y. Zhu , J. Chem. Eng. Data 2016, 61, 4139.

[295] a) J. Luo , B. Liu , R. Shi , Y. Guo , Q. Feng , Z. Liu , L. Li , K. Norinaga , Micropor. Mesopor. Mater. 2021, 327, 111404;

[296] a) Y. Liu , J. Wilcox , Environ. Sci. Technol. 2013, 47, 95; 22747244 10.1021/es3012029

[297] G. Kupgan , L. J. Abbott , K. E. Hart , C. M. Colina , Chem. Rev. 2018, 118, 5488. 29812911 10.1021/acs.chemrev.7b00691

[298] a) W. Song , J. Yao , J. Ma , A. Li , Y. Li , H. Sun , L. Zhang , Fuel 2018, 215, 196;

[299] R. Babarao , S. Dai , D.‐e. Jiang , J. Phys. Chem. C 2012, 116, 7106.

[300] Z. Zhang , D. Luo , G. Lui , G. Li , G. Jiang , Z. P. Cano , Y.‐P. Deng , X. Du , S. Yin , Y. Chen , Carbon 2019, 143, 531.

[301] D. Bahamon , A. E. Ogungbenro , M. Khaleel , M. R. Abu‐Zahra , L. F. Vega , Ind. Eng. Chem. Res. 2020, 59, 7161.

[302] a) Z. Liu , G. Zhao , X. Zhang , L. Gao , J. Chen , W. Sun , G. Zhou , G. Lu , Chin. J. Chem. Eng. 2021, 37, 46;

[303] a) G. Mazzola , S. Yunoki , S. Sorella , Nat. Commun. 2014, 5, 1; 10.1038/ncomms4487 PMC3973041 24647280

[304] T. Wang , S. Tian , G. Li , L. Zhang , M. Sheng , W. Ren , Renewable Sustainable Energy Rev. 2021, 149, 111391.

[305] a) D. Jiang , H. Li , S. Wang , X. Cheng , P. Bartocci , F. Fantozzi , Fuel 2023, 332, 125948;

[306] S. O. Adio , S. A. Ganiyu , M. Usman , I. Abdulazeez , K. Alhooshani , Chem. Eng. J. 2020, 382, 122964.

[307] Y. Oh , V.‐D. Le , U. N. Maiti , J. O. Hwang , W. J. Park , J. Lim , K. E. Lee , Y.‐S. Bae , Y.‐H. Kim , S. O. Kim , ACS Nano 2015, 9, 9148. 26267150 10.1021/acsnano.5b03400

[308] H. Yun , Y. J. Kim , S. B. Kim , H. J. Yoon , S. K. Kwak , K. B. Lee , J. Hazard. Mater. 2022, 426, 127816. 34865899 10.1016/j.jhazmat.2021.127816

[309] S. Rattanaphan , T. Rungrotmongkol , P. Kongsune , Renew. Energ. 2020, 145, 622.

[310] M. Wang , X. Fan , L. Zhang , J. Liu , B. Wang , R. Cheng , M. Li , J. Tian , J. Shi , Nanoscale 2017, 9, 17593. 29114692 10.1039/c7nr05977a

[311] H. Chen , Y. J. Zhang , P. Y. He , L. C. Liu , J. Clean. Prod. 2021, 325, 129271.

[312] N. H. M. H. Tehrani , M. S. Alivand , D. M. Maklavany , A. Rashidi , M. Samipoorgiri , A. Seif , Z. Yousefian , Chem. Eng. J. 2019, 358, 1126.

[313] H. Xu , W. Chu , X. Huang , W. Sun , C. Jiang , Z. Liu , Appl. Surf. Sci. 2016, 375, 196.

[314] X. Li , T. Guo , L. Zhu , C. Ling , Q. Xue , W. Xing , Chem. Eng. J. 2018, 338, 92.

[315] H. Chen , Y. Guo , Y. Du , X. Xu , C. Su , Z. Zeng , L. Li , Chem. Eng. J. 2021, 415, 128824.

[316] X. Ma , L. Li , R. Chen , C. Wang , H. Li , H. Li , Chem. Asian J. 2018, 13, 2069.

[317] G. Lim , K. B. Lee , H. C. Ham , J. Phys. Chem. C 2016, 120, 8087.

[318] a) K. T. Butler , D. W. Davies , H. Cartwright , O. Isayev , A. Walsh , Nature 2018, 559, 547; 30046072 10.1038/s41586-018-0337-2

[319] a) A. L. Samuel , IBM J. Res. Dev. 1967, 11, 601;

[320] M. Hutson , Science 2019, 365, 416. 31371586 10.1126/science.365.6452.416

[321] K. M. Jablonka , D. Ongari , S. M. Moosavi , B. Smit , Chem. Rev. 2020, 120, 8066. 32520531 10.1021/acs.chemrev.0c00004 PMC7453404

[322] M. Riedmiller , Comput. Stand. Interfaces 1994, 16, 265.

[323] M. E. Celebi , K. Aydin , Unsupervised Learning Algorithms, Springer, 2016.

[324] L. P. Kaelbling , M. L. Littman , A. W. Moore , J. Artif. Intell. Res. 1996, 4, 237.

[325] R. Batra , L. Song , R. Ramprasad , Nat. Rev. Mater. 2021, 6, 655.

[326] M. Zhong , K. Tran , Y. Min , C. Wang , Z. Wang , C.‐T. Dinh , P. De Luna , Z. Yu , A. S. Rasouli , P. Brodersen , Nature 2020, 581, 178. 32405017 10.1038/s41586-020-2242-8

[327] A. H. Farmahini , S. Krishnamurthy , D. Friedrich , S. Brandani , L. Sarkisov , Chem. Rev. 2021, 121, 10666. 34374527 10.1021/acs.chemrev.0c01266 PMC8431366

[328] X. Yuan , J. Wang , S. Deng , M. Suvarna , X. Wang , W. Zhang , S. T. Hamilton , A. Alahmed , A. Jamal , A.‐H. A. Park , Renewable Sustainable Energy Rev. 2022, 162, 112413.

[329] S. G. Subraveti , Z. Li , V. Prasad , A. Rajendran , Ind. Eng. Chem. Res. 2019, 58, 20412.

[330] a) Y. Sun , R. F. DeJaco , J. I. Siepmann , Chem. Sci. 2019, 10, 4377; 31057764 10.1039/c8sc05340e PMC6482883

[331] S. Kolbadinejad , H. Mashhadimoslem , A. Ghaemi , M. Bastos‐Neto , Chem. Eng. Processing‐Process Intensif. 2022, 170, 108662.

[332] a) Y. S. Bae , R. Q. Snurr , Angew. Chem., Int. Ed. 2011, 50, 11586; 10.1002/anie.201101891 22021216

[333] N. Susarla , R. Haghpanah , I. Karimi , S. Farooq , A. Rajendran , L. S. C. Tan , J. S. T. Lim , Chem. Eng. Res. Des. 2015, 102, 354.

[334] X. Ma , W. Xu , R. Su , L. Shao , Z. Zeng , L. Li , H. Wang , Sep. Purif. Technol. 2023, 306, 122521.

[335] S. Gupta , L. Li , JOM 2022, 74, 414.

[336] Q. Wang , C. Zheng , X. Wu , M. Wang , Fuel 2022, 321, 124071.

[337] M. Rahimi , S. M. Moosavi , B. Smit , T. A. Hatton , Cell Rep. Phys. Sci. 2021, 2, 100396.

[338] C. Zhang , D. Li , Y. Xie , D. Stalla , P. Hua , D. T. Nguyen , M. Xin , J. Lin , Fuel 2021, 290, 120080.

[339] M. Rahimi , M. H. Abbaspour‐Fard , A. Rohani , J. Power Sources 2022, 521, 230968.

[340] S. Wang , Z. Zhang , S. Dai , D.‐e. Jiang , ACS Mater. Lett. 2019, 1, 558.

[341] S. Wang , Y. Li , S. Dai , D. e. Jiang , Angew. Chem. 2020, 132, 19813. 10.1002/anie.202005931 32485029

[342] W. K. P. Wickramaarachchi , M. Minakshi , X. Gao , R. Dabare , K. W. Wong , Chem. Eng. J. Adv. 2021, 8, 100158.

[343] B. Li , S. Wang , Z. Tian , G. Yao , H. Li , L. Chen , Adv. Theory Simul. 2022, 5, 2100378.

[344] A. Rostami , M. A. Anbaz , H. R. E. Gahrooei , M. Arabloo , A. Bahadori , Egypt. J. Pet. 2018, 27, 65.

